# A Unitary Association-based conodont biozonation of the Smithian–Spathian boundary (Early Triassic) and associated biotic crisis from South China

**DOI:** 10.1186/s13358-022-00259-x

**Published:** 2022-11-22

**Authors:** Marc Leu, Hugo Bucher, Torsten Vennemann, Borhan Bagherpour, Cheng Ji, Morgane Brosse, Nicolas Goudemand

**Affiliations:** 1 Paleontological Institute and Museum, University of Zurich, Karl-Schmid-Strasse 4, 8006 Zurich, Switzerland; 2 Institute of Earth Surface Dynamics, University of Lausanne, Géopolis, 1015 Lausanne, Switzerland; 3 Department of Earth Sciences, Faculty of Sciences, Shiraz University, Shiraz, Iran; 4 State Key Laboratory of Palaeobiology and Stratigraphy, Nanjing Institute of Geology and Palaeontology and Center for Excellence in Life and Paleoenvironment, Chinese Academy of Sciences, 39 East Beijing Road, Nanjing, 210008 China; 5 Eawag, Überlandstrasse 133, 8600 Dübendorf, Switzerland; 6 Institut de Génomique Fonctionnelle de Lyon, CNRS UMR 5242, Univ. Lyon, ENS de Lyon, 46 allée d’Italie, 69364 Lyon Cedex 07, France

**Keywords:** Smithian–Spathian boundary, Unitary associations, Conodonts, South China

## Abstract

The Smithian–Spathian boundary (SSB) crisis played a prominent role in resetting the evolution and diversity of the nekton (ammonoids and conodonts) during the Early Triassic recovery. The late Smithian nektonic crisis culminated at the SSB, ca. 2.7 Myr after the Permian–Triassic boundary mass extinction. An accurate and high-resolution biochronological frame is needed for establishing patterns of extinction and re-diversification of this crisis. Here, we propose a new biochronological frame for conodonts that is based on the Unitary Associations Method (UAM). In this new time frame, the SSB can thus be placed between the climax of the extinction and the onset of the re-diversification. Based on the study of new and rich conodont collections obtained from five sections (of which four are newly described here) in the Nanpanjiang Basin, South China, we have performed a thorough taxonomical revision and described one new genus and 21 new species. Additionally, we have critically reassessed the published conodont data from 16 other sections from South China, and we have used this new, standardized dataset to construct the most accurate, highly resolved, and laterally reproducible biozonation of the Smithian to early Spathian interval for South China. The resulting 11 Unitary Association Zones (UAZ) are intercalibrated with lithological and chemostratigraphical (δ^13^C_carb_) markers, as well as with ammonoid zones, thus providing a firm basis for an evolutionary meaningful and laterally consistent definition of the SSB. Our UAZ_8,_ which is characterized by the occurrence of *Icriospathodus* ex gr. *crassatus*, *Triassospathodus symmetricus* and *Novispathodus brevissimus*, is marked by a new evolutionary radiation of both conodonts and ammonoids and is within a positive peak in the carbon isotope record. Consequently, we propose to place the SSB within the separation interval intercalated between UAZ_7_ and UAZ_8_ thus leaving some flexibility for future refinement and updating.

**ZooBank LSID:** urn:lsid:zoobank.org:pub:12326FE6-79DD-43A8-9421-26C3883FBE2E

**Supplementary Information:**

The online version contains supplementary material available at 10.1186/s13358-022-00259-x.

## Introduction

Ca. 2.7 Myr after the Permian–Triassic boundary mass extinction (PTBME), at the Smithian–Spathian boundary (SSB), nektopelagic organisms such as ammonoids and conodonts suffered their severest loss of diversity within the Triassic (Brayard & Bucher, 2015; Orchard, 2007; Stanley, 2009; Zhang et al., 2019). The SSB crisis has long been overlooked and considered as part of a protracted Early Triassic environmental stress that would have prohibited complete recovery of the biosphere until the Middle Triassic (e.g. Alroy, 2008). It has now become clear that episodic abiotic environmental stresses were still of high amplitude during the Early Triassic and that this time interval witnessed at least three biotic crises: at the Griesbachian–Dienerian boundary (GDB), at the Dienerian–Smithian boundary (or Induan–Olenekian boundary, IOB) and at the SSB (e.g. Wei et al., 2015). Furthermore, the recovery dynamics of benthonic organisms (Friesenbichler et al., 2019; Hautmann et al., 2015; Hofmann et al., 2014) contrast with those of nektonic ones (Brayard & Bucher, 2015; Brayard et al., 2009a, 2009b; Brühwiler et al., 2010; Ware et al., 2015), the latter being clearly affected only by the SSB crisis. This crisis is associated with major, global environmental changes, whose role is still debated: the palynological record suggests a transition from a humid (spore-dominated) climate during the middle Smithian to a drier (conifer-dominated) climate during the late Smithian and the Spathian (Hochuli et al., 2016; Schneebeli-Hermann et al., 2012); the stable oxygen isotopic composition of conodont elements (Romano et al., 2013; Sun et al., 2012) and the geographical repartition of segminiplanate forms of conodonts (Leu et al., 2019) reveal a major (7 to 8 °C) cooling of Tethyan waters that initiated during the late Smithian *Glyptophiceras–Xenoceltites* ammonoid zone (Goudemand et al., 2019). Moreover, the late Smithian is marked by a global and conspicuous positive excursion of the stable carbon isotope record (Galfetti et al., 2007; Payne et al., 2004; Wei et al., 2015).

Because of their high evolutionary rates, ammonoids and conodonts are ideal clades for biochronology, especially during the Early Triassic. During the Smithian and Spathian both clades experienced unusually high evolutionary rates and were abundant and widespread (Brayard & Bucher, 2015; Orchard, 2007). Thanks to their tiny size and their phosphatic composition, conodont elements have a good preservation potential and abundant collections can be recovered from nearly any marine sedimentary rock of that age, allowing for worldwide correlations and unsurpassed spatial and temporal resolution.

**Fig. 1:**
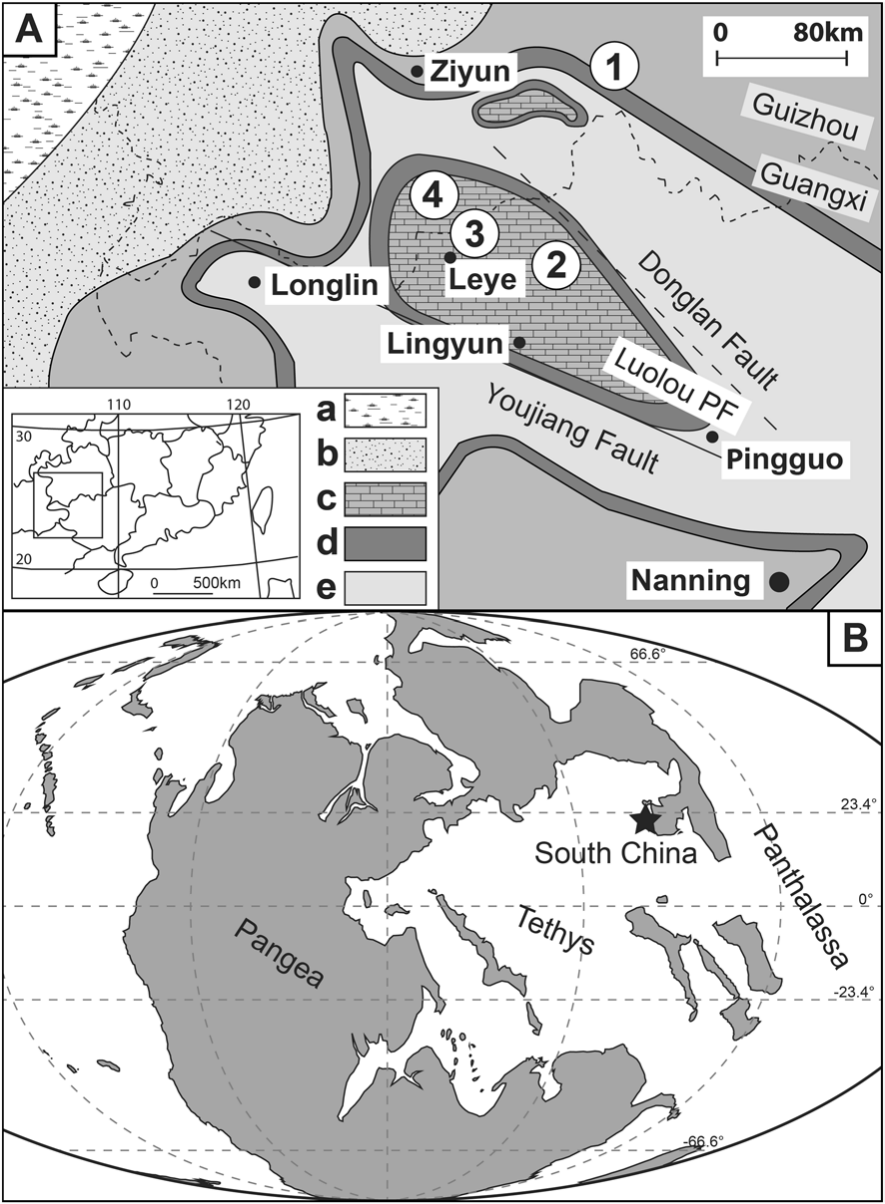
**A** Locations of the studied sections in the Nanpanjiang Basin (Luolou platform) (modified from Bagherpour et al., 2017). a—flood alluvial facies, b—shallow water siliclastic deposit, c—carbonate platform, d—slope, e—basin. Sections: 1—Qiakong, 2—Laren, 3—Shanggang, 4—Lilong, 5—Youping cascade. **B** Simplified palaeogeographical map of the Early Triassic (modified after the PANALESIS plate tectonic model of Vérard, 2019) South China indicated with a star

Although SSB conodonts are known from many localities around the globe, including British Columbia (Orchard & Zonneveld, 2009), the Canadian Arctic (Orchard, 2008), Spitzbergen (Nakrem et al., 2008), Siberia (Dagis, 1984), Western USA (Maekawa & Jenks, 2021; Solien, 1979) Europe (Chen et al., 2016; Kolar-Jurkovšek et al., 2017, 2021), Japan (Maekawa et al., 2018), Oman (Chen et al., 2019), and the Northern Indian Margin (NIM, including southern Tibet) (Brühwiler et al., 2009; Goel, 1977; Lyu et al., 2021; Matsuda, 1981, 1982, 1983, 1984; Romano et al., 2013; Sun et al., 2021; Sweet, 1970; Tian et al., 1983), the South China Block is certainly the most studied and one of the best documented areas for that interval (e.g. Zhang et al., 2019). Most sections are found in the Nanpanjiang Basin in the south western part of the block, and in northern part of the block (Yangtse Platform), as well. During the Early Triassic, the South China Block occupied an equatorial position in the eastern Tethys (Fig. 1).

Classic sections from the Yangtze platform include Meishan (e.g. Jiang et al., 2007), North Pingdingshan (Zhao et al., 2007) and West Pingdingshan (Liang et al., 2011). Other conodont-rich sections from the northern part of the South China Block include Huangzhishan (Chen et al., 2008), Chaotian (Ji et al., 2011), Shangsi (Jiang et al., 2011), Daxiakou (Zhao et al., 2013) Jianshi (Lyu et al., 2019), Ganxi (Lyu et al., 2019), South Majiashan (Zhao et al., 2007), Qingshan (Liu et al., 2019; Qiu et al., 2019), Longtan (Liu et al., 2019) and Yiwagou (Li et al., 2019).

The Nanpanjiang basin, presently situated at the southern margin of the South China Block, includes several isolated platforms and extends as far south as the “An Chau” basin in northern Vietnam (Enos, 2006; Feng et al., 2015; Galfetti et al., 2008; Komatsu et al., 2016), where intercalated volcanic rocks become abundant. In the Nanpanjiang basin, Early Triassic marine record includes abundant benthos, ammonoids and conodonts (e.g. Brayard & Bucher, 2008; Foster et al., 2019; Hautmann et al., 2011; Wu et al., 2019b). Detailed Early Triassic conodont biostratigraphical analyses have been performed in a number of sections including Guandao (Lehrmann et al., 2015), Gaimao (Yang et al., 2012), Dawen (Chen et al., 2009), Dajiang (Jiang et al., 2014), Mingtang (Liang et al., 2016), Jiarong (Chen et al., 2015), Sidazhai (Liang, 2017), Bianyang (Yan et al., 2013) and Qingyan (Ji et al., 2011) in southern Guizhou, and Zuodeng (Tong et al., 2007, revised after Yang et al., 1984), Motianling (Wu et al., 2019a), and Wuzhuan (Brosse et al., 2015) in northwestern Guangxi.

However, to date, most biochronological studies used continuous interval zones. These are prone to diachronism, largely because the time of the first occurrences (FO) of index species may vary across different sections. Moreover, the fossil record is intrinsically discrete and incomplete, which leads to frequent contradictions in the observed relative sequences of FOs. These contradictions may originate from local ecological controls, sampling effort, selective preservation, disjunctive reworking, condensation, and taxonomic inconsistencies. In our view, the best method to detect and consistently solve these contradictions are discrete maximal association zones such as those constructed by means of the Unitary Association (UA) method (e.g. Brosse et al., 2016; Guex, 1991; Monnet & Bucher, 2002). For the SSB, only a few recent studies have been based on this method: Wu et al. (2019b) constructed a biozonation for South China that includes 26 sections; Chen et al. (2019) constructed a Tethyan biozonation using four SSB sections, two from Oman, one from Slovenia and the well-documented Jiarong section from South China. Yet, these two studies led to strikingly different sets of characteristic species and they diverged in their respective positioning of the SSB.

Based on the study of new and rich conodont collections from five sections (of which four are newly described here) from the Nanpanjiang basin, South China, we perform here a thorough taxonomical revision and describe a series of new taxa. By critically reassessing the published conodont data from 16 other sections from South China, and by applying the UA method to our taxonomically updated dataset, we constructed a new conodont-based biozonation of the Smithian to Early Spathian interval for South China with no internal contradictions and which is consistent with the global carbon isotope signal and the ammonoid zonation. This new UA-based zonation is, in our view, more accurate than the previously available schemes.

## Geological setting

During the Late Permian to Early Triassic, the Nanpanjiang basin evolved as a pull-apart basin in a back-arc context (Bagherpour et al., 2017; Duan et al., 2020). The basin is presently exposed across Guangxi, southern Guizhou and eastern Yunnan, as well as northern Vietnam. This basin (also called Guizhou–Guangxi–Hunan Basin, see Feng et al., 2015) was an epicontinental sea situated in the equatorial realm between the eastern Tethys (Yangtze Platform) and the western Panthalassa Ocean (Enos, 2006). The five newly described sections are located in the present-day Guangxi and Guizhou provinces (see Fig. 1).

**Fig. 2:**
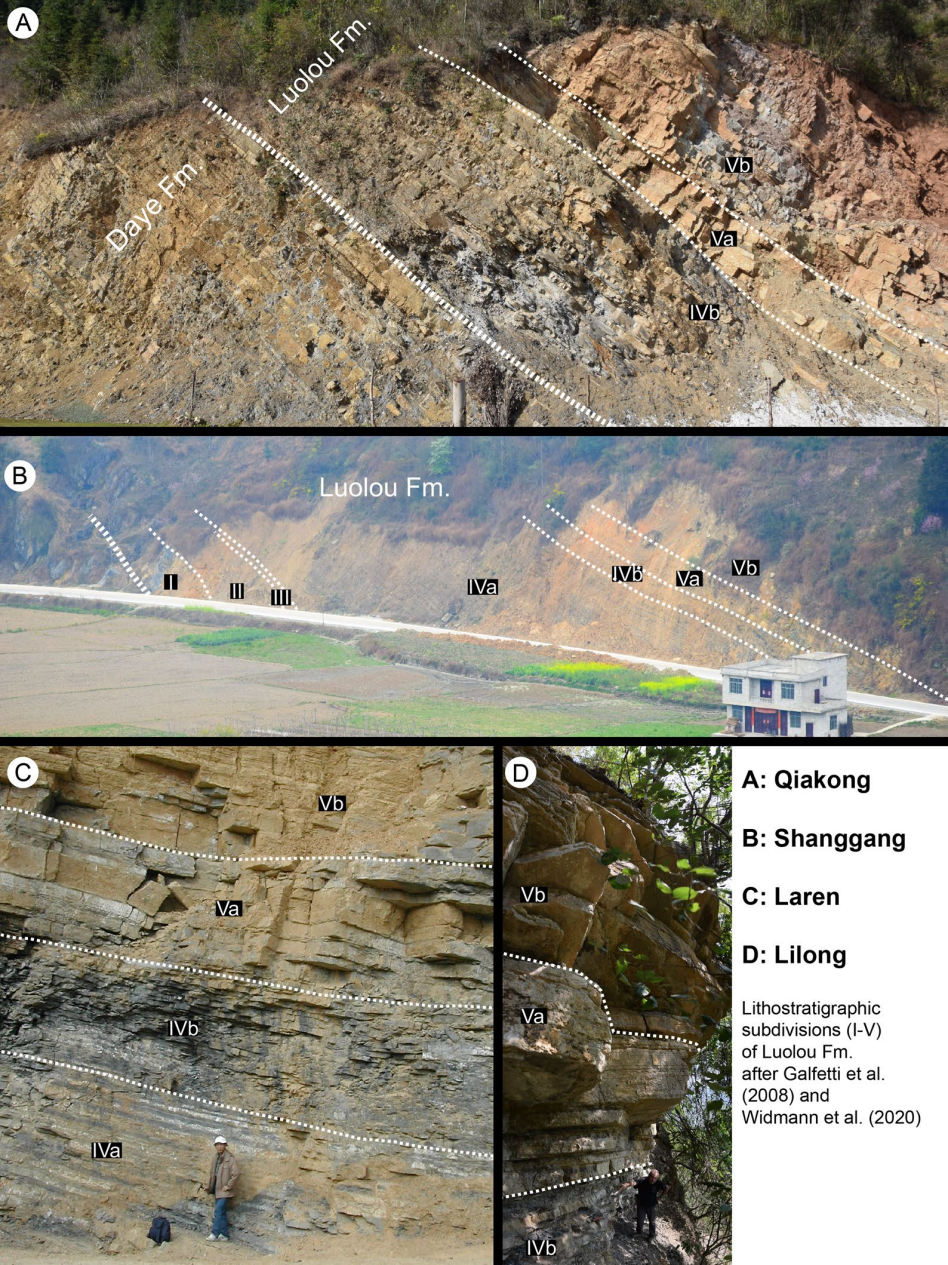
Pictures from four of the five studied sections in the Nanpanjiang basin. **A** Qiakong, **B** Shanggang, **C** Laren (Kuang Guodun for scale), **D** Lilong (Hugo Bucher for scale)

The Lilong (24° 54′ 41.40ʺ N 106° 32′ 11.00ʺ E), Shanggang (24° 48′ 44.40ʺ N 106° 32′ 31.90ʺ E), Laren (24° 36′ 25.30ʺ N 106° 52′ 40.70ʺ E), Banhaipo (24° 37′ 58.80ʺ N 106° 51′ 43.40ʺ E) and Youping cascade sections (24° 55′ 26.12ʺ N, 106° 31′ 42.63ʺ E) are located in the northern part of Guangxi province. These sections all belong to the Luolou Formation, an Early Triassic outer platform setting (Galfetti et al., 2008) whose paleogeographic distribution defines the largest know platform in the north-central part of the Nanpanjiang Basin (Bagherpour et al., 2017). The Luolou Formation is an ammonoid- and conodont-rich sedimentary succession (see Fig. 2), whose subdivision into units (equivalent to members) has been established by Galfetti et al. (2008). The Smithian and Spathian units comprise, in ascending order: (1) a light-grey, bioturbated, thin-bedded, ammonoid-rich “*Flemingites*”-limestone (unit III, Early Smithian); (2) mixed thin-bedded, dark, finely laminated limestone alternating with dark silty shales (unit IVa, middle Smithian); (3) black shales intercalated with thin, dark limestone beds (unit IVb, mostly late Smithian); (4) a prominent, cliff-forming, light-grey, medium to thick-bedded, nodular limestone (unit V, Spathian).

The Qiakong section (25° 51′ 26.38ʺ N 107° 18′ 32.09ʺ E) is situated in the southern part of the Guizhou province, within the Pingtang syncline (Bagherpour et al., 2020), which belongs to the north-eastern edge of the Nanpanjiang basin. Within this syncline, the Griesbachian to middle Smithian succession belongs to the Daye Formation consisting essentially of basinal deposits (black shales of Griesbachian age) grading upwards into thick slope deposits (thin-bedded limestone with traction patterns) of Smithian age. Here the Daye Formation is overlain by an expanded version of the Luolou Formation, but whose age ranges only from the late Smithian to the late Spathian (see Fig. 2).

## Material and methods

We collected 180 conodont samples, 3 to 8 kg each. Furthermore, fist-sized samples were also taken for C-isotope analysis from the same beds as the conodont samples. In order to build a taxonomically consistent data set before the analysis by means of Unitary Associations, we also reassessed published conodont data from 16 additional SSB sections that were included.

### Conodont sampling and preparation

The samples were collected from the limestone beds across Smithian and Spathian strata of the Luolou Formation. The spacing between samples varies from 10 cm to 2 m. Highest resolution sampling was achieved near the Smithian–Spathian transition. We collected 49 conodont samples from Qiakong, 57 from Laren, 38 from Shanggang, 21 from Lilong, and 15 from Youping cascade, respectively. The samples were dissolved with a ~ 10% buffered acetic acid solution following the procedure after Jeppsson et al. (1999). The residues were concentrated via heavy liquid separation using sodium polytungstate (Jeppsson & Anehus, 1999) and sieved with a 0.075-mm mesh. The heavy fraction was handpicked under a binocular microscope and selected conodont elements were illustrated using a scattered electron microscope (SEM) (JEOL JSM-6010).

### Carbon isotopes

In Early Triassic studies, it has become a frequent and useful approach to analyse carbon isotopes along with biochronology. The combination of these two methods provides a further test for the consistency of the results, and we follow this usage here. With the exception of the carbon isotope carbon record from Youping Cascade which is here newly documented, all other carbon isotope records illustrated here were already made available by Widmann et al. (2020). Therefore, carbon isotope records are here only shown graphically for the purpose of intercalibration and coherence with the new biochronological frame. For the carbonate carbon isotope measurements, heterogeneous samples were carefully cleaned and cut into slabs in order to recover unaltered micrite. The most homogenous part was drilled on a sawed surface with a diamond-tipped drill to produce a fine powder. The isotopic composition of the carbonates was measured with a GasBench II connected to a Finnigan MST DeltaPlus XL mass spectrometer, using a HE-carrier gas system, after the method from Spötl and Vennemann (2003). Reproducibility of replicate analysis was better than ± 0.1% for standards and ± 0.15% for sedimentary samples. All reported isotopic results are in standard δ notation (VPDB).

### Biochronology

**Fig. 3:**
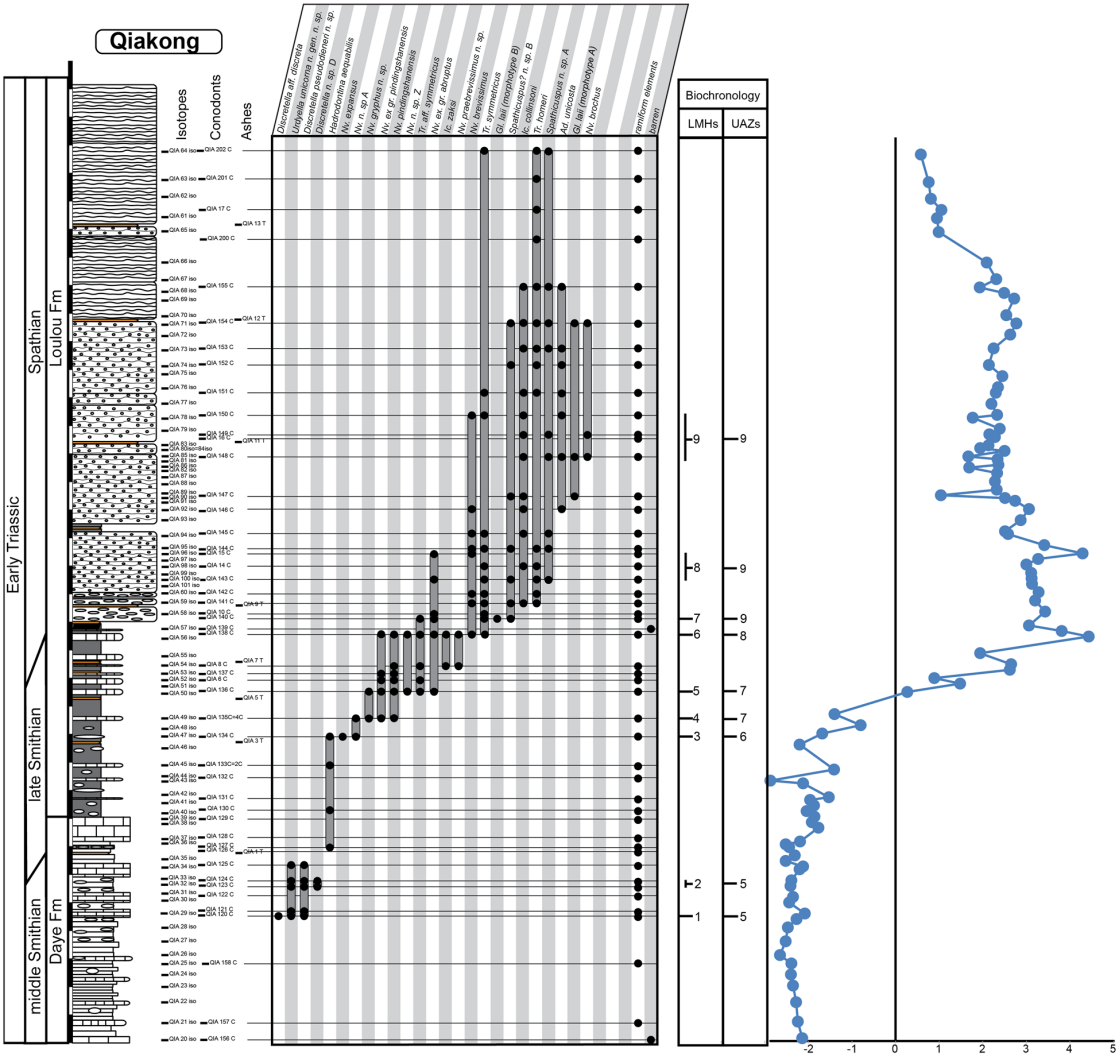
Qiakong. Detailed stratigraphic log of the studied interval of the Qiakong section showing the distribution of conodont taxa and the δ^13^C_carb_ record throughout the middle Smithian part of the Daye Formation and the late Smithian to Spathian Luolou Formation. The LMHs and UAZs are indicated

**Fig. 4:**
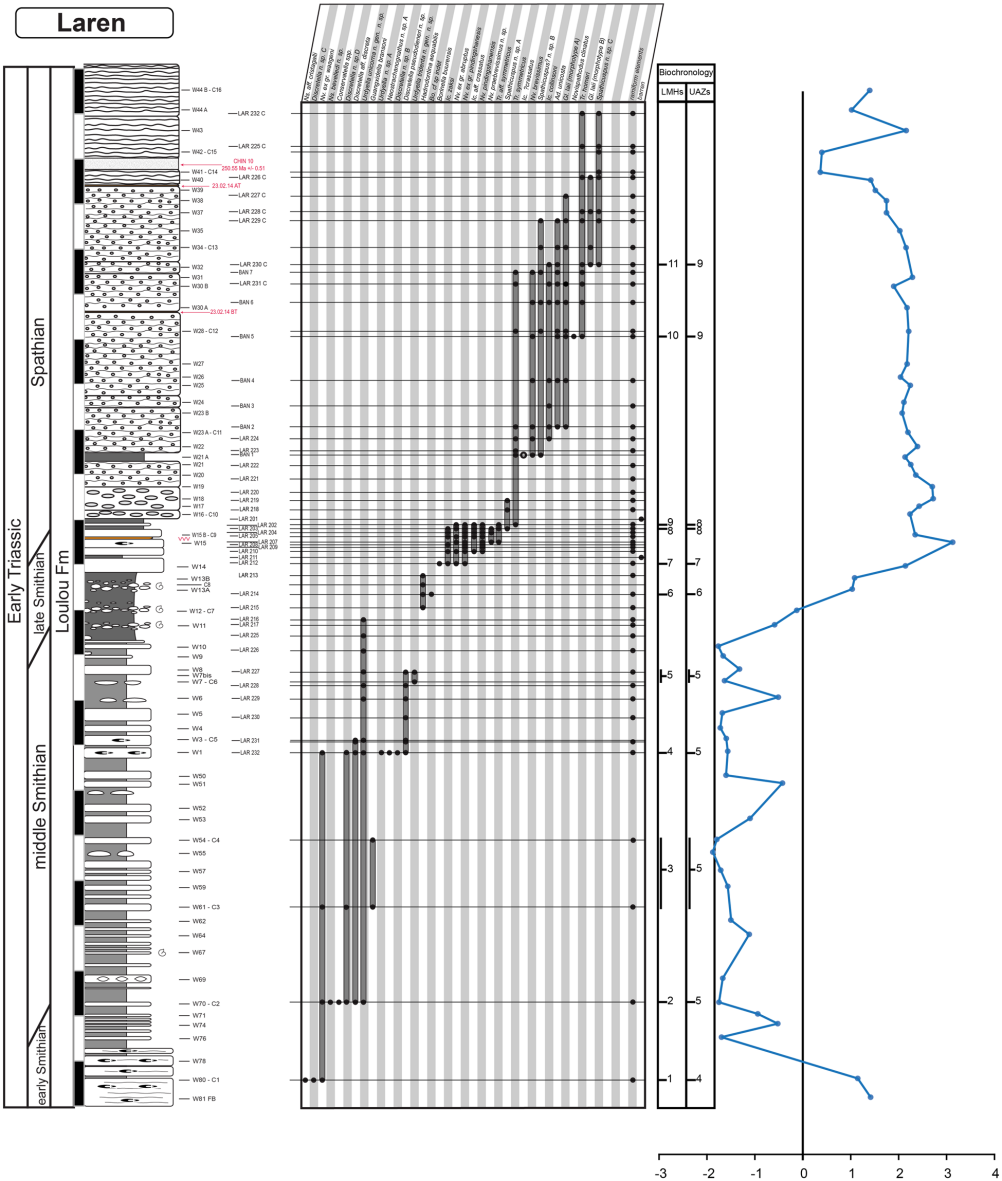
Laren. Detailed stratigraphic log of the studied interval of the Laren section showing the distribution of conodont taxa and the δ^13^C_carb_ record throughout the Smithian and Spathian part of the Luolou Formation. The LMHs and UAZs are indicated

**Fig. 5:**
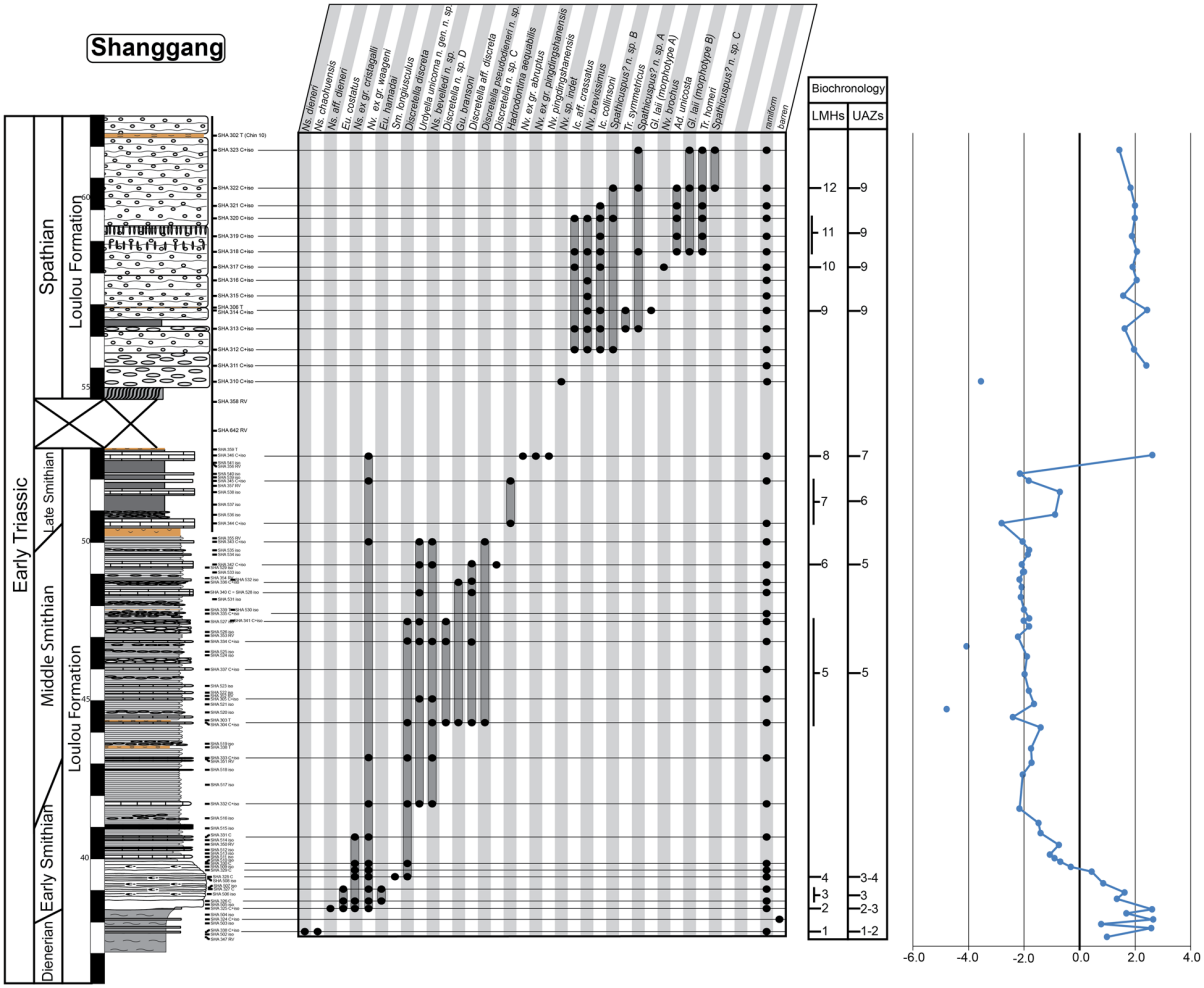
Shanggang. Detailed stratigraphic log of the studied interval of the Shanggang section showing the distribution of conodont taxa and the δ^13^C_carb_ record throughout the Smithian and Spathian part of the Luolou Formation. The LMHs and UAZ are indicated. Note the gap because of low angle faulting in the upper part of the black shales

**Fig. 6:**
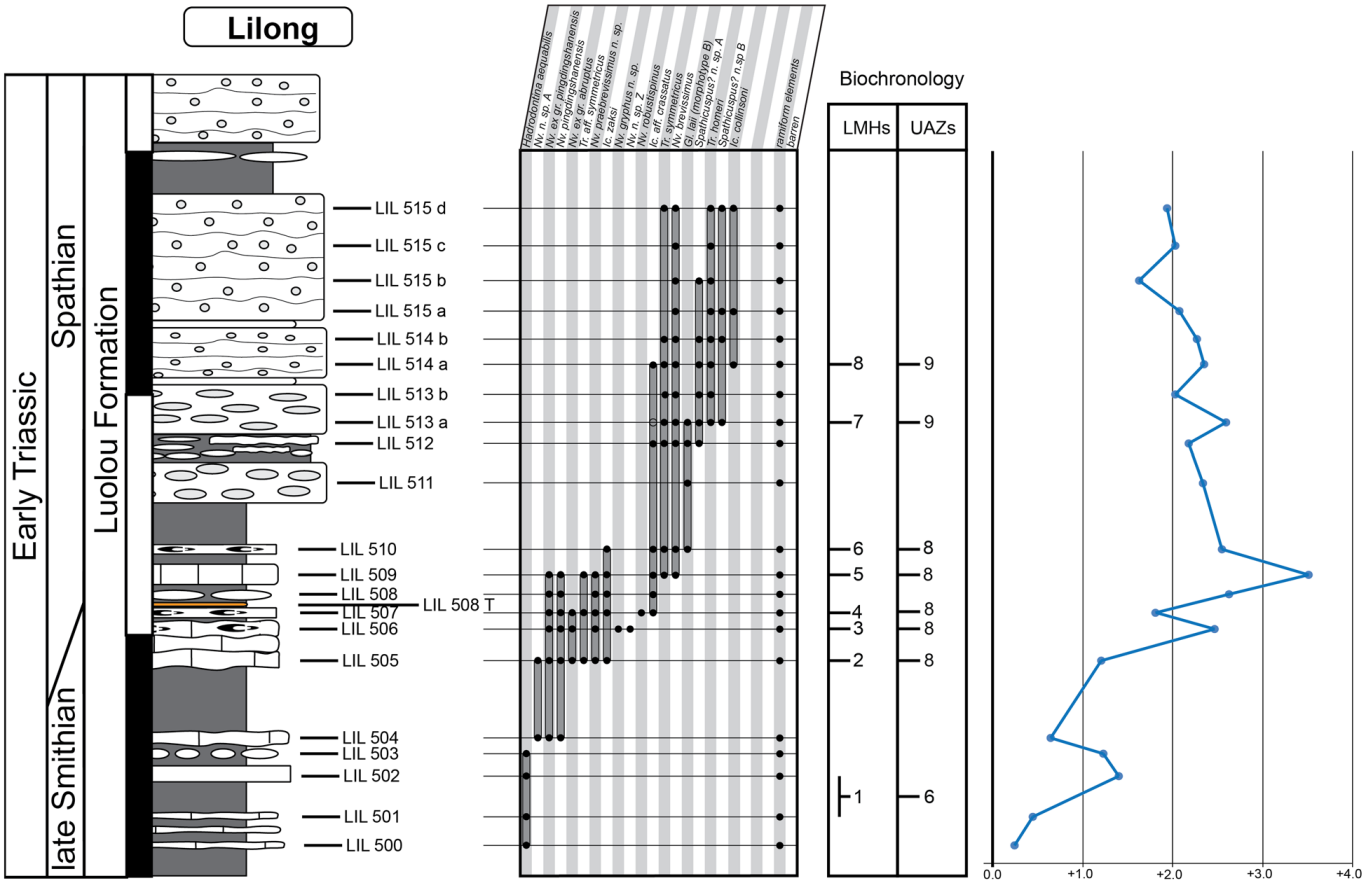
Lilong. Detailed stratigraphic log of the studied interval of the Lilong section showing the distribution of conodont taxa and the δ^13^C_carb_ record throughout the late Smithian and Early Spathian part of the Luolou Formation. The LMHs and UAZs are indicated

**Fig. 7:**
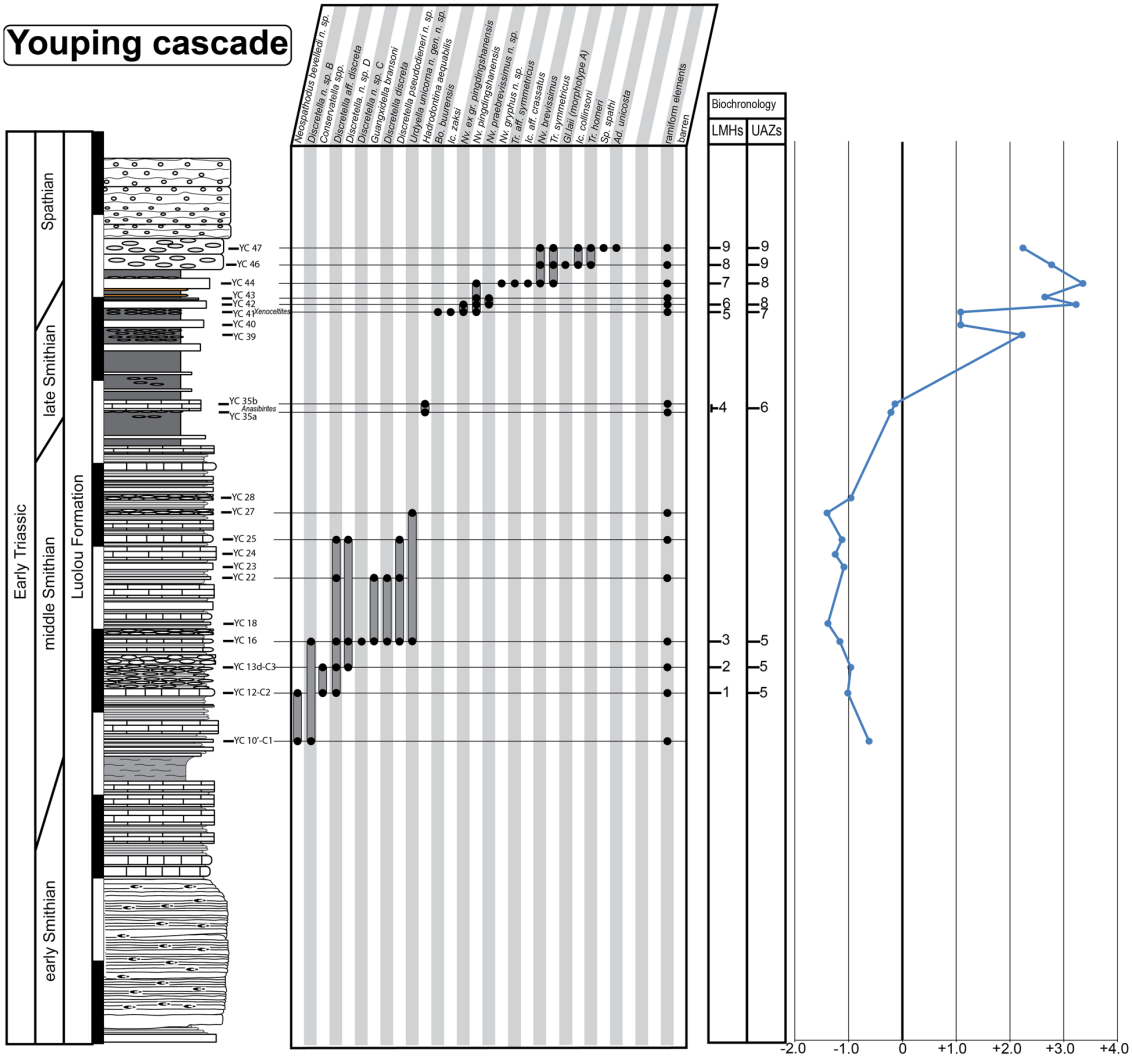
Youping Cascade. Detailed stratigraphic log of the studied interval of the Youping Cascade section showing the distribution of conodont taxa and the δ^13^C_carb_ record throughout the late Smithian and Early Spathian part of the Luolou Formation. The LMHs and UAZs are indicated

For the construction of the biochronological scheme, specimens left in open nomenclature were excluded from the dataset. In addition to the new data presented here, we have included the published data from 16 well-sampled and well-documented sections, in particular those for which key taxa had been illustrated. These sections include Jiarong (Chen et al., 2015, 2019), Motianling (Wu et al., 2019a), Mingtang (Liang et al., 2016), Guandao (Lehrmann et al., 2015), Bianyang (Yan et al., 2013), Qingyan (Ji et al., 2011), Sidazhai (Liang, 2017), Ganheqiao (Liang, 2017) for the Nanpanjiang basin, and Daxiakou (Zhao et al., 2013), Ganxi (Lyu et al., 2019), Yiwagou (Li et al., 2019) Jurong, Qingshan and Longtan (Liu et al., 2019), West Pingdingshan (Liang et al., 2011; Zhao, 2005; Zhao et al., 2007, 2008), North Pingdingshan (Zhao et al., 2007, 2008) for the Yangtze Platform (see Additional file 1). The raw data of 12 of these SSB sections were obtained from the synthesis papers of Wu et al. (2019b) and Chen et al. (2019). We have used the original descriptions and illustrations to assess whether and where our newly defined taxa may have occurred in these sections and for taxonomic consistency in general. Their occurrence tables were completed and revised accordingly (see Figs. 3, 4, 5, 6, 7, “Systematic palaeontology” section and Additional files 2 and 3). For the published sections that span more than the Smithian and Spathian, we have taken into consideration the interval bound by the first occurrence (FO) of *Neospathodus dieneri* to the last occurrence (LO) of *Chiosella timorensis*.

We have used the Unitary Association method (Brosse et al., 2016; Guex, 1991; Monnet & Bucher, 2002) as implemented within the software PAST (Hammer et al., 2001) in its 2013 version (Version 2.17c, year 2013), which unlike the most recent versions enables to detect and analyse Z_4_-type contradictions. The taxa found only in one section were temporarily removed from the analysis via the “null endemic taxa” option.

The first run with the full initial data set (see Additional file 2) led to the detection of 453 contradictions, of which 31 cycles between cliques and a large number of Z4 cycles (313), suggesting a rather low internal consistency of the initial data.

If we exclude the Sidazhai and Ganheqiao sections the number of contradictions drops to 164, the number of Z_4_ cycles to 100 and cycles between cliques to 0: hence about two-thirds of all contradictions are generated by these two noisy sections. A critical view of the source data (Liang, 2017) reveals that these faunas include a mixture of Smithian taxa with Spathian ones. This mixing likely originates from the occurrence of limestone breccias (in Sidazhai) and possibly from poorly resolved taxonomy. Therefore, we excluded these two sections from the subsequent analyses.

One of the main advantages of the UA method is that not only it detects automatically biostratigraphic contradictions, but it allows establishing the respective contributions of the different taxa involved. These contradictions may result from misidentifications, condensation, reworking, insufficient sampling effort, or from sparse and too poorly constrained biostratigraphic relations (i.e. low-quality record). We have iteratively modified the data, e.g. by reassessing some identifications and revising the corresponding occurrences, by expanding the local range of some taxa when their co-occurrence with other taxa has been documented elsewhere, or in the worst case by excluding some taxa if they were involved in too many contradictions. The latter case usually involves rare and/or long ranging taxa, thereby implying under-constrained biostratigraphic relationships. This selective process is applied iteratively until a minimum amount of contradictions is reached (Guex, 1991). The details of this procedure can be found in Additional file 3.

In the present case, all contradictions and cycles between cliques could be eliminated and only 5 minor contradictions (Z_4_ cycles) did remain, in which *Neospathodus spitiensis* and *Pachycladina peculiaris* are the most frequently involved taxa. Because the stratigraphic range of these two species does not overlap with the SSB, we have not tried to solve these minor contradictions.

**Fig. 8:**
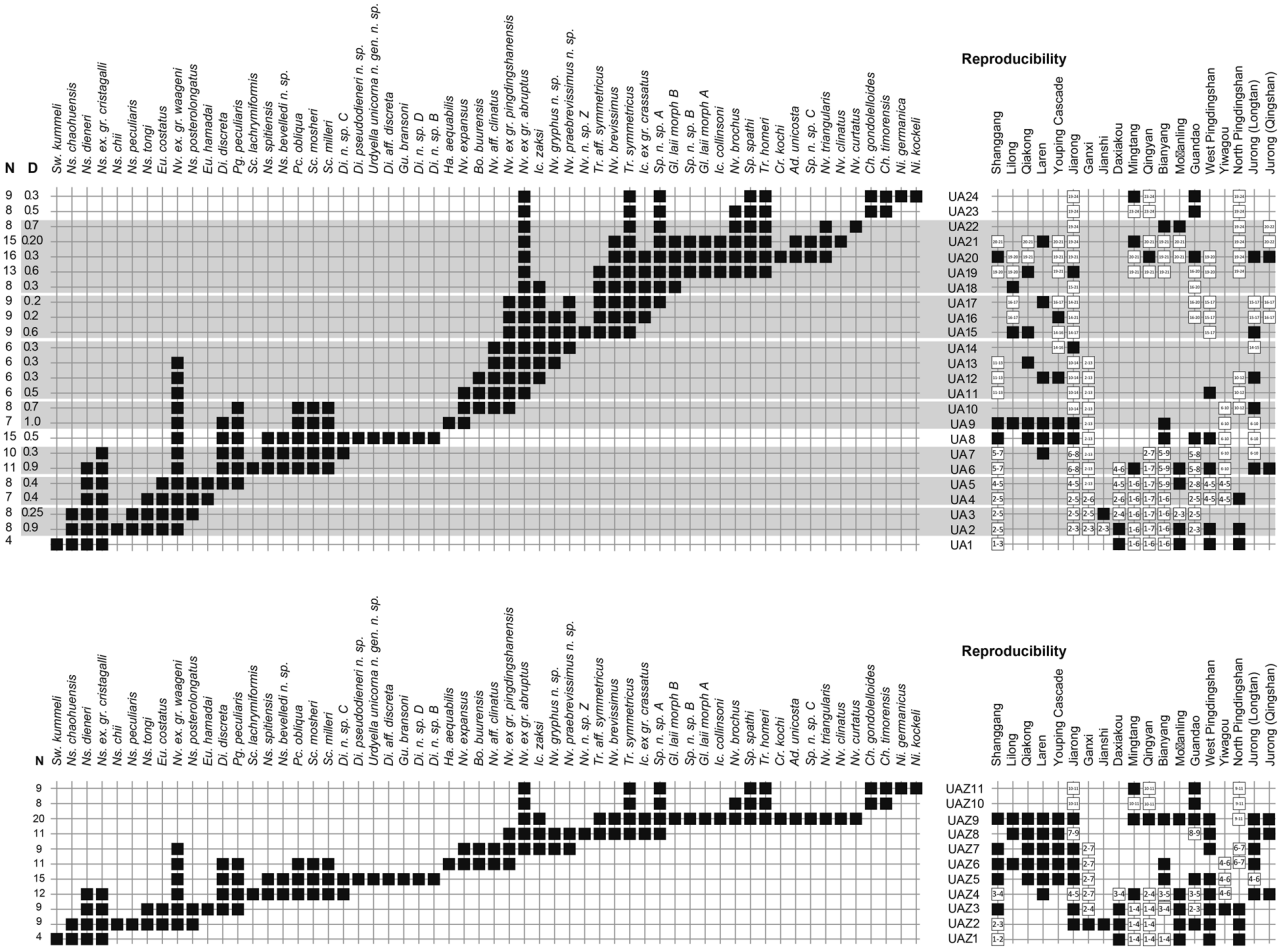
Sequences of Unitary Association (UAs), Unitary Associations Zones (UAZs), lateral reproducibility and dissimilarity index (D) resulting from the biochronological analyses of the 19 sections in South China from the final run. Note the grey shades in the upper figure with rather poor lateral reproducibility and/or poor dissimilarity index of the UA_2_, UA_3_, UA_4_, UA_5_, UA_6_, UA_7_, UA_9_, UA_10_, UA_11_, UA_12_, UA_13_, UA_14_, UA_15_, UA_16_, UA_17_, UA_18_, UA_19_, UA_20_, UA_21_ and UA_22_. This 20 UAs were merged into a final total of 11 UAZs (lower figure)

This last run produced a total of 24 preliminary Unitary Associations (UAs). Because only UAs that are laterally reproducible, that is, UAs that occur in several sections, are actually useful for correlation purposes, we followed the propositions of PAST (see the reproducibility matrix in Fig. 8) and merged the following UAs: UA_2–3_, UA_4–5_, UA_6–7_ UA_9–10_, UA_11–14,_ UA_15–17_ and UA_18–22_. This merging step results in fewer UAs but the resulting Unitary Association Zones (UAZ) have a higher lateral reproducibility (in other words their spatial scope is enhanced) and hence, are more robust and more useful than the preliminary UAs.

## Results

### Carbon isotope record

**Fig. 9:**
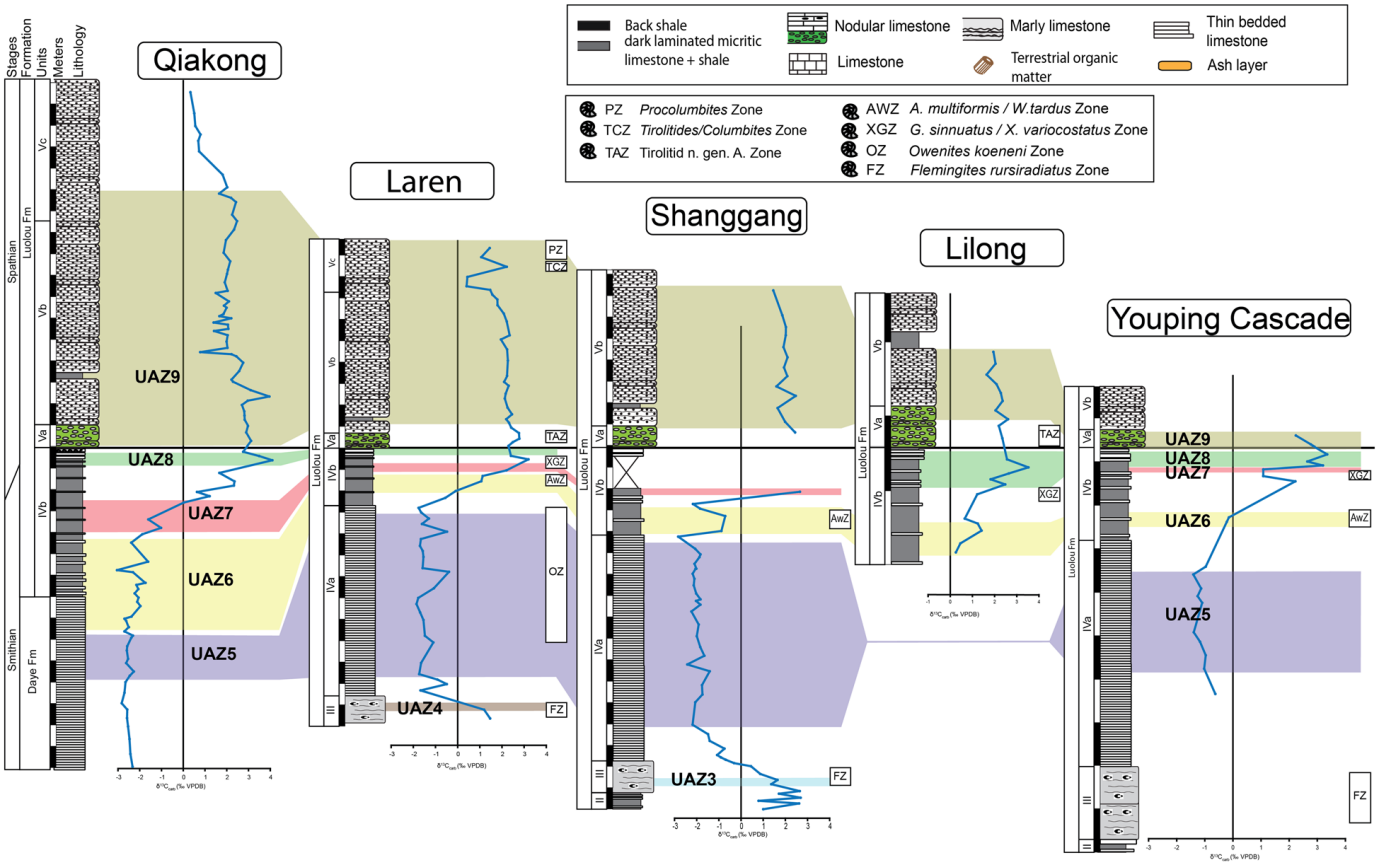
Position of the UAZs in the studied sections Qiakong, Laren, Shanggang, Lilong and Youping Cascade. The lithological units are simplified and represented from unit III to unit Vc. Note the ammonoid zones recorded from Laren, Shanggang, Lilong and Youping Cascade

The stable carbon isotope data from all the studied sections, including the new data from Youping Cascade (see Figs. 7, 9 and Additional file 4), display consistent trends: a negative excursion of the δ^13^C_carb_ in the *Flemingites* beds, a negative plateau around − 2‰ in the *Owenites* beds, a prominent positive excursion in the late Smithian interval peaking at the top of unit IVb within the black shales, and a negative excursion at or closely following the abrupt lithological change from black shales to nodular limestones (from unit IVb to unit Va). The greatest variability (up to 3–4‰) of the δ^13^C_carb_ values occurred during the middle and late Smithian, where the deeper water section (Qiakong) shows the most negative values (Fig. 12). This variability noticeably decreases to ca. 2‰ in the Spathian. Meyer et al. (2011) interpreted such difference as a vertical depth gradient, thus suggesting a weakening of this gradient across the SSB.

### Conodonts

The CAI (Conodont Alteration Index) in Qiakong is ~ 3 (brownish colour). In all other studied sections, the CAI was around 5 (black colour). All sampled sections yielded abundant conodont material. Only 5 out of 172 samples were barren, namely samples SHA 324C (Shanggang), QIA 156 C, QIA 139 C (both Qiakong), LAR 211 and LAR 201 (both Laren). Abundance of conodont elements is the highest in units III and IVb. In all sections, the segminate elements were recovered in much higher abundance than segminiplanate elements. Segminiplanate forms are completely absent from the *Flemingites* beds (early Smithian) and the *Owenites* beds (middle Smithian).

In total, we have recovered elements belonging to 46 conodont species assigned to 16 genera. 40 of these species are illustrated and discussed in the “Systematic palaeontology” section. In particular, we describe 21 new species: *Neospathodus bevelledi* n. sp., *Novispathodus gryphus* n. sp., *Novispathodus* n. sp. Z, *Novispathodus praebrevissimus* n. sp., *Discretella pseudodieneri* n. sp., *Urdyella unicorna* n. gen. n. sp., *Urdyella tridenta* n. gen. n. sp., *Gladigondolella laii* (morphotype A and B), *Novispathodus* n. sp. A, *Discretella*? n. sp. B, *Discretella*? n. sp. C, *Discretella*? n. sp. D, *Spathicuspus* n. sp. A, *Spathicuspus*? n. sp. B, *Spathicuspus*? n. sp. C, *Urdyella* n. sp. A, *Neostrachanognathus* n. sp. A, *Triassospathodus* aff. *symmetricus*, *Icriospathodus* aff. crassatus and *Discretella* aff. *discreta*. The new forms for which only a small number of specimens (< 10) were recovered and/or the preservation was considered inadequate, were left in open nomenclature. Finally, we revised the diagnosis of two species: *Icriospathodus zaksi* and *Hadrodontina aequabilis*.

### Description of the biozonation

The final biozonation is based on 58 conodont taxa from 19 sections and it comprises 11 Unitary Association Zones (UAZ) (Fig. 8). We propose (see below) to place the SSB within the separation interval intercalated between UAZ_7_ and UAZ_8_. Five sections record both the late Smithian UAZ_7_ and the earliest Spathian UAZ_8_: Qiakong, Laren, Youping Cascade, West Pingdingshan and Longtan (Jurong) (see Figs. 3, 4, 5, 6, 7, 8, 9).

#### UAZ_1_

Content: *Sw. kummeli*, *Ns. dieneri*, *Ns. chaohuensis* and *Ns*. ex. gr. *cristagalli*.

Age: Dienerian.

Geographical distribution: Daxiakou, Motianling, West- and North Pingdingshan.

Characteristic species: *Sw. kummeli*.

This UAZ occurs in the *Ns. dieneri* interval zone of Motianling, and Daxiakou and in the *Ns. kummeli* interval zone of North- and West Pingdingshan.

#### UAZ_2_

Content: *Ns. dieneri*, *Ns. chaohuensis*, *Ns*. ex. gr. *cristagalli*, *Ns. chii*, *Ns. tongi*, *Ns. peculiaris*, *Ns. posterolongatus*, *Nv*. ex gr. *waageni* and *Eu. costatus.*

Age: early Smithian.

Geographical distribution: Jiarong, Ganxi, Jianshi, Daxiakou, Motianling, Guandao, West- and North Pingdingshan.

Characteristic species: *Ns. chii* and *Ns. peculiaris*.

Characteristic pairs of species: *Ns. chaohuensis* with either *Ns. tongi*, *Eu. costatus*, *Ns. posterolongatus* or *Nv*. ex. gr. *waageni*.

UAZ_2_ overlaps with the *Flemingites* beds in Daxiakou, West- and North Pingdingshan (Zhao et al., 2007, 2013).

#### UAZ_3_

Content: *Ns. dieneri*, *Ns*. ex. gr. *cristagalli*, *Ns. tongi*, *Ns. posterolongatus*, *Nv*. ex gr. *waageni*, *Eu. costatus, Eu. hamadai, Di. discreta* and *Pg. peculiaris.*

Age: early Smithian.

Geographical distribution: Shanggang, Jiarong, Daxiakou, Motianling, Yiwagou, West- and North Pingdingshan.

Characteristic species: *Eu. hamadai*.

Characteristic pairs of species: *Ns. tongi* or *Eu. costatus* with either *Di. discreta* or *Pg. peculiaris*.

In Shanggang, UAZ_3_ overlaps with the beginning of the negative carbon isotope excursion (CIE). In deeper water sections such as North- and West Pingdingshan, UAZ_3_ falls within the ill-defined “*Flemingites–Euflemingites* Zone”, which is confusingly reported to include middle Smithian genera (Tong & Zakharov, 2004).

#### UAZ_4_

Content: *Ns. dieneri*, *Ns*. ex. gr. *cristagalli*, *Nv*. ex gr. *waageni, Di. discreta, Pg. peculiaris, Sc. lachrymiformis, Ns. spitiensis, Ns. bevelledi, Pc. obliqua, Sc. mosheri, Sc. milleri, Di. n. sp. C.*

Age: Early to middle Smithian.

Geographical distribution: Laren, Mingtang, Motianling, Yiwagou and West Pingdingshan.

Characteristic species: *Sc. lachrymiformis*. So far, this species has been documented only from the northern marginal basin of the Yangtze platform (e.g. in Yiwagou) and is apparently missing in the Nanpanjiang basin. However, UAZ_4_ is identified in the Nanpanjiang basin by means of its characteristic pairs of species.

Characteristic pairs of species: *Ns. dieneri* or *Ns*. ex. gr. *cristagall*i with either *Ns. spitiensis*, *Ns. bevelledi*, *Pc. obliqua*, *Sc. mosheri*, *Sc. milleri*, or *Di*. n. sp. C.

In Laren, UAZ_4_ overlaps is within the negative carbon isotope shift that culminates higher up during the middle Smithian. UAZ_4_ is still within unit III which corresponds to the early Smithian *Flemingites* Beds. Similarly, UAZ_4_ overlaps with the end of the negative CIE (beds 13–15) in Mingtang.

#### UAZ_5_

Content: *Nv*. ex gr. *waageni, Di. discreta, Pg. peculiaris, Ns. spitiensis, Ns. bevelledi, Pc. obliqua, Sc. mosheri, Sc. milleri, Di. n. sp. C, Ns. pseudodieneri, Ur. unicorna, Di.* aff*. discreta, Gu. bransoni, Di. n. sp. D and Di. n. sp. B.*

Age: middle Smithian.

Geographical distribution: Shanggang, Qiakong, Laren, Youping Cascade, Jiarong, Bianyang, Guandao and West Pingdingshan.

Characteristic species: *Ns. pseudodieneri, Ur. unicorna, Di.* aff*. discreta, Gu. bransoni, Di. n. sp. D* and *Di. n. sp. B.*

UAZ_5_ has an excellent lateral reproducibility in the newly documented sections. UAZ_5_ largely overlaps with the *Owenites* beds in Laren. In Qiakong, UAZ_5_ occurs within the Daye Fm., which represents slope deposits typically yielding a meagre conodont record. In Laren, Shanggang and Youping Cascade, this zone is within unit IVa, where it coincides with the negative plateau of the stable carbon isotope curve. In Jiarong, UAZ_5_ corresponds to sample JRC-39 (Chen et al., 2015). UAZ_5_ partly overlaps with the *Nv. waageni* interval zone in West Pingdingshan, and with the *Di. discreta* interval zone of Jiarong and Bianyang.

#### UAZ_6_

Content: *Nv*. ex gr. *waageni, Di. discreta, Pg. peculiaris, Pc. obliqua, Sc. mosheri, Sc. milleri, Ha. aequabilis, Nv. expansus, Bo. buurensis, Nv. aff. clinatus* and *Nv.* ex gr*. pingdingshanensis.*

Age: late Smithian.

Geographical distribution: Shanggang, Lilong, Qiakong, Laren, Youping Cascade, Jiarong, Bianyang, and Jurong (Longtan).

Characteristic species: *Hadrodontina aequabilis.*

Characteristic pairs of species: either *Di. discreta*, *Pg. peculiaris*, *Pc. obliqua*, *Sc. mosheri*, or *Sc. milleri* with either *Nv. expansus*, *Bo. buurensis*, *Nv*. aff. *clinatus*, or *Nv.* ex gr*. pingdingshanensis*.

UAZ_6_ has an excellent lateral reproducibility and occurs in all five newly described sections. UAZ_6_ concurs with the late Smithian ammonoid *Anasibirites* beds as documented in Laren, Shanggang and Youping Cascade. UAZ_6_ occurs in the lower part (Shanggang, Qiakong, Youping Cascade) and in the middle part (Laren) of Unit IVb. In Jiarong and Bianyang, UAZ_6_ corresponds to the *Parachirognathus–Pachycladina* assemblage zone. In Longtan (Jurong), UAZ_6_ partly overlaps with the lower part of the *Nv. pingdingshanensis* interval zone (Bed 57). Apart from *Nv.* ex gr*. pingdingshanensis*, all the taxa found in this zone are traditionally considered to be typical Smithian taxa. Similarly, the *Anasibirites* beds classically define the early late Smithian.

#### UAZ_7_

Content: *Nv*. ex gr. *waageni, Nv. expansus, Bo. buurensis, Nv. aff. clinatus* and *Nv.* ex gr. *pingdingshanensis, Nv. ex gr. abruptus, Ic. zaksi, Nv. gryphus* n. sp. *and Nv. praebrevissimus* n. sp.

Age: late Smithian.

Geographical distribution: Shanggang, Qiakong, Laren, Youping Cascade, Jiarong, West Pingdingshan and Jurong (Longtan).

Characteristic pairs of species: either *Nv*. ex gr. *waageni*, *Nv. expansus*, *Bo. buurensis*, or *Nv. aff. clinatus* with either *Nv. abruptus*, *Ic. zaksi*, *Nv. gryphus* n. sp. or *Nv. praebrevissimus* n. sp.

UAZ_7_ occurs in Qiakong, Laren, Shanggang and Youping Cascade. UAZ_7_ occurs within unit IVb and within the late Smithian positive CIE in all studied sections. In Jiarong and West Pingdingshan, UAZ_7_ partly overlaps with the *Nv pingdingshanensis* interval zone and is within the positive CIE (and beds JB-11 and JC-5 in Jiarong). The ammonoid *Glyptophiceras–Xenoceltites* beds are also included within UAZ_7_ in Laren and Youping Cascade. This ammonoid zone has been usually regarded as late Smithian in age. In terms of conodonts, this zone is composed by the association of ‘typical Smithian’ forms such as *Nv*. ex gr. *waageni* and *Bo. buurensis* with ‘typical Spathian’ forms such as *Nv. abruptus* and *Ic. zaksi*. Here, we assign a late Smithian age to this zone because of the clear ammonoid intercalibration.

#### UAZ_8_

Content: *Nv. ex gr. pingdingshanensis, Nv.* ex. gr. *abruptus, Ic. zaksi, Nv. gryphus* n. sp.*, Nv. praebrevissimus* n. sp.*, Nv.* n. sp. Z*, Tr.* aff*. symmetricus, Nv. brevissimus, Tr. symmetricus, Ic.* ex gr. *crassatus* and *Sp.* n. sp. A.

Age: early Spathian.

Geographical distribution: Lilong, Qiakong, Laren, Youping Cascade, West Pingdingshan and Jurong (Longtan and Qingshan).

Characteristic species: *Nv.* n. sp. Z.

Characteristic pairs of species: either *Nv*. ex. gr. *pingdingshanensis*, *Nv. gryphus* n. sp., or *Nv. praebrevissimus* n. sp. with either *Tr*. aff. *symmetricus*, *Nv. brevissimus*, *Tr. symmetricus*, *Ic.* ex gr. *crassatus*, or *Sp*. n. sp. A.

UAZ_8_ consistently occurs in the upper part of unit IVb in all our newly described sections, except Shanggang where the top of Unit IVb has been obscured by a low angle fault.

All the conodont taxa found in this zone are traditionally considered to be typical Spathian taxa. As a consequence, this zone is considered as the oldest one known from the Spathian, and the SSB must be placed within the interval of separation that intercalates between UAZ_7_ and UAZ_8_.

In Qiakong, Laren and Youping Cascade, the SSB can be unambiguously placed in the interval of separation between UAZ_7_ and UAZ_8_. In Lilong, the SSB is located within the thin interval bracketed by *Xenoceltites*–*Glyptophiceras* beds below and UAZ_8_ above, hence ca. 50 cm below the sharp lithological boundary between units IVb and Va. In all these sections, UAZ_8_ overlaps with the positive peak of the CIE except in Laren where the peak is located within the uncertainty interval between UAZ_7_ and UAZ_8_. In Longtan, UAZ_8_ is recognized in bed 70, which separates the *Nv. pingdingshanensis* and the *Tr*. aff. *homeri* interval zones, whereas in Qingshan, UAZ_8_ overlaps only with the *Nv. pingdingshanensis* interval zone (bed 70). In West Pingdingshan this zone is recognized within bed 54 and it overlaps with the positive peak of the CIE, as well.

#### UAZ_9_

Content: *Nv.* ex. gr. *abruptus, Ic. zaksi, Tr.* aff*. symmetricus, Nv. brevissimus, Tr. symmetricus, Ic.* ex gr. *crassatus, Sp.* n. sp. A*, Gl*. *laii* (morph. B), *Sp*. n. sp. B, *Gl*. *laii* (morph. A), *Ic. collinsoni*, *Nv. brochus*, *Sp. spathi*, *Tr. homeri*, *Cr. kochi, Ad. unicosta*, *Sp*. n. sp. C, *Nv. triangularis*, *Nv. clinatus*, *Nv. curtatus*.

Age: early-to-middle Spathian.

Geographical distribution: Shanggang, Lilong, Qiakong, Laren, Youping Cascade, Jiarong, Mingtang, Qingyan, Bianyang, Motianling, Guandao, West Pingdingshan and Jurong (Longtan and Qingshan).

Characteristic species: *Gl*. *laii* (morph. B), *Sp*. n. sp. B, *Gl*. *laii* (morph. A), *Ic. collinsoni*, *Cr. kochi, Ad. unicosta*, *Sp*. n. sp. C, *Nv. triangularis*, *Nv. clinatus*, and *Nv. curtatus*.

Characteristic pairs of species: either *Ic*. *zaksi*, *Tr*. aff. *symmetricus*, *Nv*. *brevissimus*, or *Ic.* ex gr. *crassatus* with either *Nv. brochus*, *Sp. spathi*, or *Tr. homeri*.

This UA zone is recognized in all newly studied sections. In Qiakong and Youping Cascade, the base of UAZ_9_ corresponds to the lower limit of Unit Va. In Qiakong and Laren, UAZ_9_ ranges up into unit Vc and in Laren, UAZ_9_ includes the Tirolitid n. gen A beds, the *Tirolites/Columbites* beds and the middle Spathian *Procolumbites* beds. UAZ_9_ is the laterally most reproducible zone and occurs in all investigated sections. AZ_9_ also includes the *Ic. collinsoni*, *Tr. homeri* and *Nv. triangularis* interval zones (e.g. Jiarong, Bianyang, Mingtang, Jurong, Motianling and West Pingdingshan).

#### UAZ_10_

Content: *Nv.* ex. gr. *abruptus, Tr. symmetricus, Sp.* n. sp. A*, Nv. brochus*, *Sp. spathi*, *Tr. homeri*, *Ch. gondolelloides, Ch. timorensis*.

Age: late Spathian.

Geographical distribution: Guandao.

Characteristic pairs of species: *Nv. brochus* with either *Ch. gondolelloides*, or *Ch. timorensis.*

#### UAZ_11_

Content: *Nv.* ex. gr. *abruptus, Tr. symmetricus, Sp.* n. sp. A*, Sp. spathi*, *Tr. homeri*, *Ch. gondolelloides, Ch. timorensis, Ni. germanicus, Ni. kockeli*.

Age: Anisian.

Geographical distribution: Mingtang and Guandao.

Characteristic species: *Ni. germanicus* and *Ni. kockeli*.

## Discussion

### Comparison with other SSB UA-based conodont biozonations in South China

Wu et al. (2019b) have recently presented a UA-based biozonation using 72 conodont species from 28 sections in South China. The time interval they studied spans from the latest Permian to the earliest Middle Triassic and led to a succession of 26 UAZ (note that our UAZ numbering is distinct from theirs). However, their study did not address several critical issues pertaining to the quality of raw data, most importantly the Sidazhai and Ganheqiao sections as mentioned above. For instance, their UAZ_19_ of supposed Spathian age, includes *Ns. dieneri*, a taxon that is usually restricted to the Dienerian and earliest Smithian. This unusual occurrence roots into the work of Liang et al. (2016), who illustrated a specimen of ‘*Ns. dieneri’* from a Spathian horizon (their fig. 4.5 from bed 18 in Mingtang section). Although Wu et al. (2019b) noticed that this was an incorrect identification (their Table 2), they did not update the data set accordingly. Apart from Liang et al. (2016), there is no other known report of *Ns. dieneri* from the Spathian. Lehrmann et al. (2015) reported the occurrence of the typically Smithian *Guangxidella bransoni* in a Spathian sample on the basis of a single broken element whose diagnostic cusp is missing (their fig. 5.18, sample WG-82), thus making this identification problematic. Zhao et al. (2013) reported *Nv. pingdingshanensis* from early Smithian strata at Daxiakou (their bed 91) on the basis of a single broken specimen whose diagnostic lower margin is missing (their fig. 11.I; note that the captions of their figs. 10 and 11 have been mistakenly swapped). This single specimen can probably be assigned to *Nv. waageni*, thus leading to a virtual co-occurrence with *Eurygnathodus hamadai*. Finally, the upward extension of *Nv. waageni* so that it intersects with *Tr. homeri* as done by Wu et al., (2019b, Additional files 2 and 3) is an unwarranted assumption that could never be confirmed by real samples. In our view, the above-mentioned occurrences and associations are questionable, either from a taxonomic and/or a stratigraphic point of view. In this work, we have revised these conflicting data before the analysis, thus explaining the differences with the earlier zonation of Wu et al. (2019b).

Chen et al. (2019) published a UA-based SSB conodont biozonation with an extremely heterogenous paleobiogeographic scope. Their data set comprises two sections from Oman, one from Slovenia and one (Jiarong) from South China. They obtained 7 UAZs and proposed to place the SSB between their UAZ_4_ and UAZ_5_ (no correspondence with our own UAZ numbering). Their biozonation suggests that *Nv*. *pingdingshanensis* is a characteristic species of their latest Smithian UAZ_4_, which is at odd with most reports documenting the association of this species with ‘typical’ Spathian taxa such as *Nv. brevissimus, Tr. symmetricus,* or *Ic.* ex gr. *crassatus.* Furthermore, most of the characteristic species of their latest Smithian and earliest Spathian zones (*Neospathodus planus* and *Neospathodus robustus* on one hand, and *Platyvillosus corniger* and *Platyvillosus regularis* on the other) are not know from South China, thus making the identification of their SSB in South China difficult. Their UAZ_2_ partly correlates with our UAZ_5_, their UAZ_3_ with our UAZ_6_, their UAZ_4_ with our UAZ_7_, and their UAZ_7_ with our UAZ_9_.

The conodont dataset produced by Widmann et al. (2020) was exclusively based on Qiakong, Shanggang, Laren and Lilong, without taking other published sections into account. The addition of 17 other sections as done here did not lead to substantial changes in the initial zonation proposed by Widmann et al. (2020). This underlines the overall quality and completeness of the conodont record in these four initial sections. The minor modifications introduced here are mostly due to the larger temporal and spatial scope of the present study. Note that the latest Smithian UAZ_7_ (equivalent to UAZ_5_ in Widmann et al., 2020) can now be recognized in Laren but no longer in Lilong, since *Nv.* n. sp. A is excluded from the present analysis.

### Comparison with carbon-isotope chemostratigraphy

The evolution of the stable carbon isotopic ratio can be an important additional proxy for Early Triassic correlations because it appears as global signal overriding local fluctuations (e.g. Payne et al., 2004). Chen et al. (2019) and Wu et al. (2019b) could not establish a clear intercalibration between their UAZs and their δ^13^C_carb_ records, whereas our new biozonation is fully consistent with the δ^13^C_carb_ fluctuations within the South China Block. Zhang et al. (2019) proposed a convenient numbering of the distinct phases of the δ^13^C_carb_ signal that we adopt here. The middle Smithian negative plateau (referred as N3, Zhang et al., 2019) always includes our UAZ_5_ in West Pingdingshan, Guandao, Bianyang, Jiarong, Shanggang, Qiakong, Laren and Youping Cascade (Chen et al., 2013, 2015; Lehrmann et al., 2015; Song et al., 2013; Tong et al., 2007; Wang et al., 2005; Widmann et al., 2020; Zhao et al., 2007; Zuo et al., 2003, 2004; Fig. 9).

The positive CIE that marks the transition from N3 to P3 in Qiakong, Laren, Shanggang, Lilong, Youping cascade, Jiarong and West Pingdingshan consistently includes UAZ_6_ and UAZ_7_. With the exception of Shanggang where P3 is removed by a low angle fault, UAZ_8_ coincides with P3 in our other sections. The same correlation can be observed in West Pingdingshan. Zhang et al. (2019) proposed to define the SSB at the midpoint of the N3-to-P3 positive shift. Because the end of the late Smithian is frequently affected by unconformities (see Hammer et al. (2019) for Spitsbergen; Widmann et al. (2020) for the Luolou Fm.) such a definition cannot be recommended. In the Luolou Fm. the abrupt end of this positive shift is an additional sign pointing to the presence of unconformities within the end of the late Smithian black shales.

### Definition of the SSB

Tozer (1967) first defined the Smithian and the Spathian stages on the basis of ammonoids. His definitions were later refined by Silberling and Tozer (1968) who placed the SSB between the *Anasibirites* beds (which correlate with our conodont UAZ_6_) and the *Tirolites*/*Columbites* beds (which correlate with our conodont UAZ_9_) in North America. In the northern Indian margin (NIM) and the Western US, an intermediate *Glyptophiceras sinuatum* Zone (Brühwiler et al., 2010; Jenks & Brayard, 2018; Jenks et al., 2015) of Smithian affinity is found above the *Anasibirites multiformis* Zone and below the typical Spathian *Tirolites*/*Columbites* beds. Since UAZ_7_ coincides with the *Glyptophiceras sinuatum* beds in South China, it seems most appropriate to place the SSB above UAZ_7_ and UAZ_8_. The latter is characterized by the overlap of *Nv. pingdingshanensis* with either *Nv. brevissimus*, *Tr. symmetricus* or *Ic.* ex gr. *crassatus,* the last three species being exclusively known from the Spathian.

Zhao et al. (2007) proposed the FO of *Nv*. *pingdingshanensis* as the main proxy for a definition of the base of the Spathian and this definition has been subsequently endorsed by many conodont workers (e.g. Lyu et al., 2021; Sun et al., 2012; Wu et al., 2019a, 2019b). Yet, we confirm that *Nv. pingdingshanensis* also occurs in the late Smithian (i.e. UAZ_6_ and UAZ_7_). Late Smithian occurrences of *Nv. pingdingshanensis* were also reported by Orchard and Zonneveld (2009), Komatsu et al. (2016), Leu et al. (2019) and Maekawa and Jenks (2021). As a consequence, the FO of *Nv. pingdingshanensis* is not a suitable proxy for the base of the Spathian because it ranges from the late Smithian into the early Spathian. Our results also indicate that *Nv. pingdingshanensis* is the only known ‘segminate’ conodont that ranges across the SSB (from UAZ_6_ to UAZ_8_), thus supporting the hypothesis that this species may be the rootstock of most Spathian segminate conodonts, i.e. representatives of *Novispathodus*, *Triassospathodus* and *Icriospathodus* (Orchard, 2007).

### Extinction and recovery patterns across the SSB

**Fig. 10:**
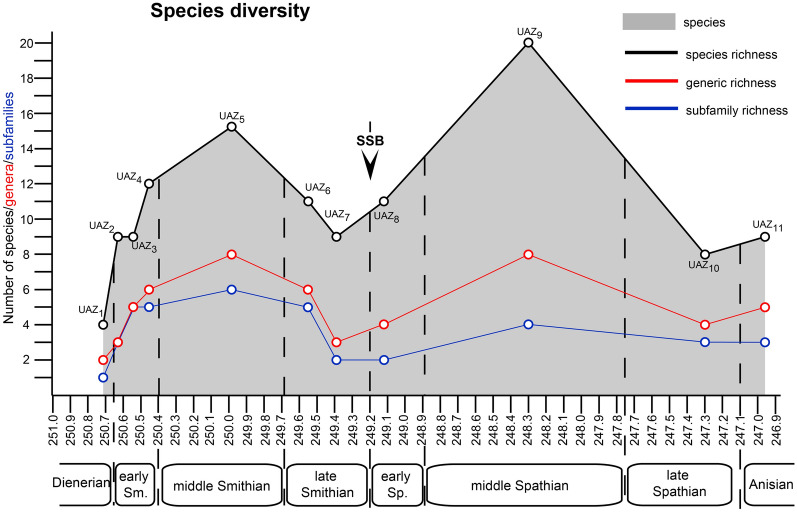
Temporal evolution of species- genera- and subfamily-diversity (succession of UAZs) for all the characteristic (part of UAZ) conodonts from South China during the Smithian and Spathian interval. Calculated from the optimal solution given in Fig. 8. Note the Early Smithian and Early Spathian radiation and the late Smithian and middle/late Spathian extinction. Absolute ages from Widmann (2019) and Widmann et al., (2020)

**Fig. 11:**
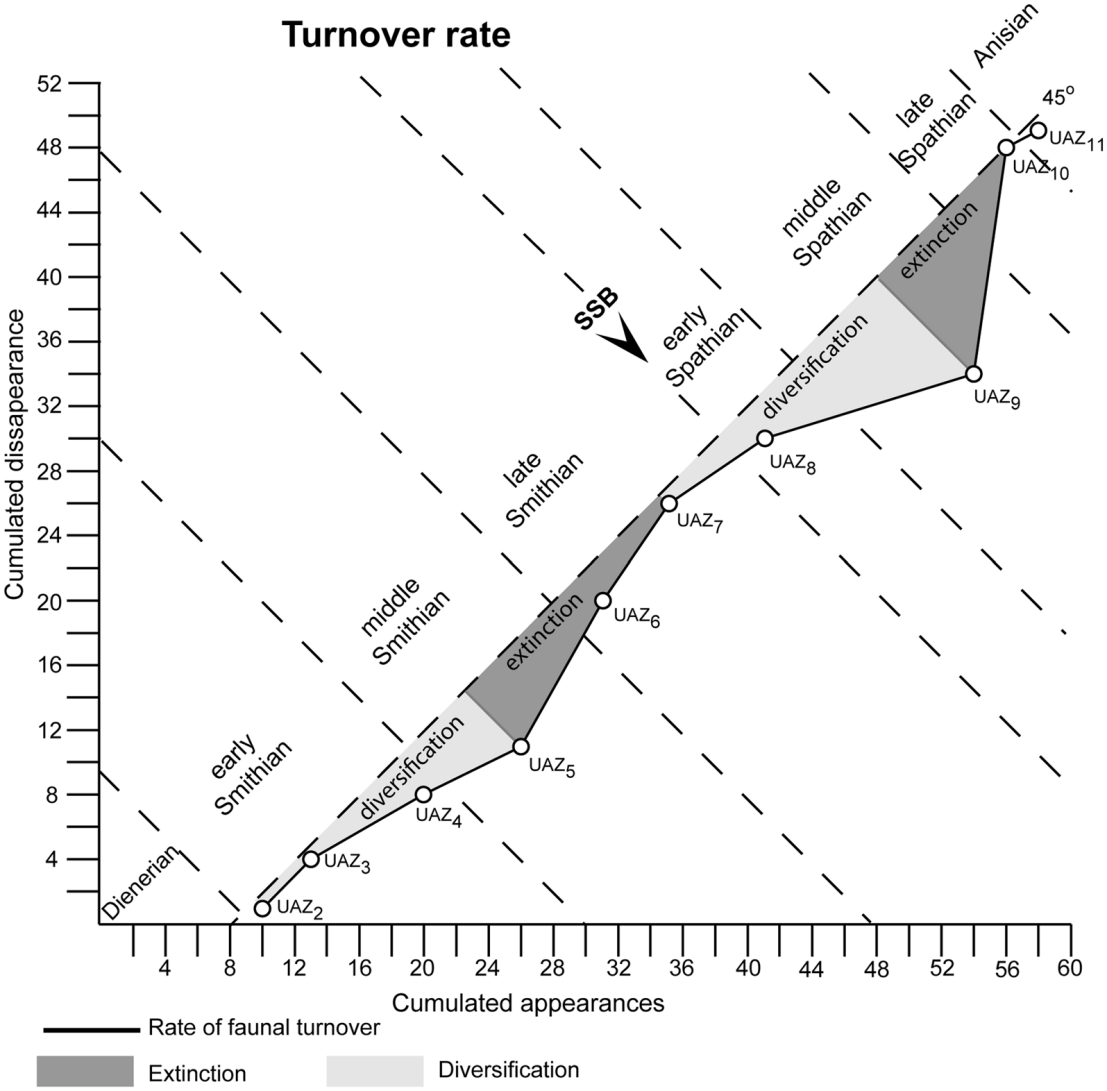
Faunal turnover rate for all the relying conodont UAZs from South China during the Smithian and Spathian interval. Calculated from the optimal solution given in Fig. 8. Note the early Smithian and early Spathian radiation and the late Smithian and middle/late Spathian extinction

As illustrated in Figs. 10 and 11, conodont taxonomic richness and turnover rate experienced two peaks during the middle Smithian and the middle Spathian, respectively. The relatively low diversity observed in the Dienerian (UAZ_1_) may be underestimated because the most relevant sections for that time interval are not included here. The early-to-middle Smithian rapid increase in conodont diversity reflects a global pattern that was first noticed by Orchard (2005, 2007). This diversity increase is also mirrored in terms of multi-element apparatuses (Orchard, 2005, 2007). This radiation initiated during the cool early Smithian (UAZ_2–4_) and culminated during the middle Smithian (UAZ_5_) thermal maximum (Goudemand et al., 2019; Romano et al., 2013).

The subsequent loss of more than a third of the conodont species between UAZ_5_ and UAZ_7_ corresponds to the largest conodont turnover of the studied interval (Figs. 8, 10 and 11). Previous interpretations (Orchard, 2007) and our preliminary observations from other basins suggest that this pattern may be of global significance. However, our refined biozonation indicates that changes in conodont diversity are not immediate and simple responses to changing trends in temperature (see Fig. 10). On the one hand, the conodont extinction already started during the end of the warm interval and continued during the late Smithian cooling. On the other hand, the early Spathian re-diversification started during the end of the cooling phase and extended further during the following warming phase. Therefore, changes in conodont diversity cannot be uniquely related to reversals in temperature trends. Moreover, segminate and segminiplanate conodonts have also been documented to have experienced opposite size trends during the late Smithian cooling (Chen et al., 2013; Leu et al., 2019). Hence, no simple causal relation can be inferred between temperature change and conodont diversity or size during the Smithian–Spathian transition. As suggested by Ginot and Goudemand (2020), other abiotic (sea level, salinity, oxygen content, etc.) and biotic factors must have come into the play.

The Spathian radiation of conodonts that spanned the time interval UAZ_7_ to UAZ_9_ (see Figs. 10 and 11) appears to be of greater magnitude than the early Smithian diversification (Fig. 11). We also note that middle Smithian and middle Spathian warm intervals (Goudemand et al., 2013, 2019) both corresponds to peak values in conodont species richness. However, the taxonomic structure of the two diversification events differed in that the Smithian one produced a higher diversity at the subfamily level than the Spathian one (see Fig. 10).

**Fig. 12:**
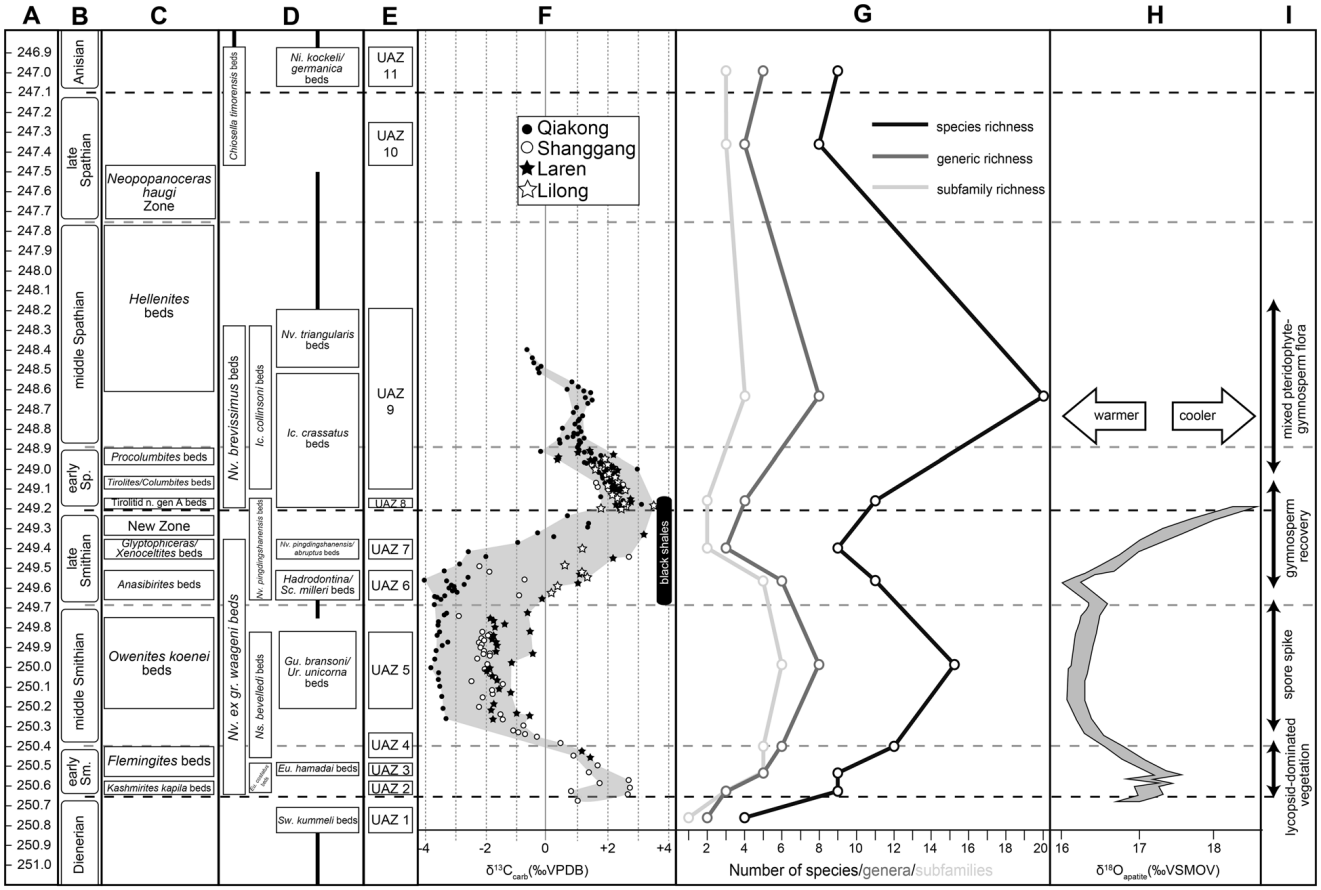
Calibration between U-Pb ages and fossiliferous beds, UAZs, δ^13^C_carb_ record, climatic proxies and conodont diversity. **A** U–Pb ages (after Widmann et al., 2020). **B** Geological timescales with Early Triassic substages **C** ammonoid biochronozones modified from Brayard and Bucher (2008), **D** Conodont beds from South China (this work), **E** Newly established conodont UAZs from South China. See Figs. 3, 4, 5, 6, 7, 8, 9. **F** Evolution of the composite δ^13^C_carb_ record during the Smithian and early Spathian from Qiakong, Laren, Shanggang, Lilong (see also Figs. 3, 4, 5, 6). **G** Temporal evolution of species- genera- and subfamily-diversity (after Fig. 10), **H** δ^18^C_phos_ from conodont apatite from Pakistan (after Goudemand et al., 2019). **I**: Palynological events (after Hermann et al. 2011)

Based on U–Pb ages provided by Widmann et al. (2020), the respective duration of our UAZs can be assessed. The Bayesian age depth model constructed by Widmann et al. (2020) suggests that the absolute ages of individual UAZs decreased from the middle Smithian to the earliest Spathian (UAZ_6–8_, Fig. 12). Absolute durations of UAZ_5_ to UAZ_9_ can be derived from this age–depth model [Fig. 12 and Additional file 5, Widmann et al. (2020)]. The resulting figures are UAZ_5_: 366 ± 193 ka; UAZ_6_: 147 ± 146 ka; UAZ_7_: 91 ± 112 ka; UAZ_8_: 7 ± 86 ka and UAZ_9_: 290 ± 89 ka. Although error margins are substantial, the duration of the zones are lowest either side of the SBB, in UAZ_7_ and UAZ_8_. This result indicates that the assumption of equal duration time bins should be used with caution when computing rates of extinction, rate of origination and turnover rate.

## Conclusion

We reported five new SSB sections from the Nanpanjiang basin (Qiakong, Shanggang, Laren, Lilong and Youping Cascade) with high-resolution conodont and stable carbon isotope sampling. Using the abundant conodont material, we described one new conodont genus and 21 new conodont species from these sections. After having standardized the conodont taxonomy on the basis of 19 SSB sections from the South China Block, we processed the updated dataset by means of the Unitary Associations Method. The resulting, new, robust, high-resolution biozonation comprises 11 UAZs whose good lateral reproducibility ensures accuracy for correlating all the sections included in the data set. The new biozonation is also intercalibrated with ammonoid biochronozones, C-isotope chemostratigraphy and lithological markers.

We propose placing the SSB between UAZ_7_, a zone characterized by the association of, for instance, *Novispathodus* ex. gr. *waageni* with either *Icriospathodus zaksi* or *Novispathodus praebrevissimus* n. sp., and UAZ_8_, a zone characterized by the association of, for instance, *Novispathodus* ex gr. *pingdingshanensis* with either *Icriospathodus.* ex gr. *crassatus* or *Triassospathodus symmetricus*. We further confirm that *Novispathodus pingdingshanensis* straddles the SSB, thus making its first occurrence a poor marker for the base of the Spathian.

## Systematic palaeontology (Leu and Goudemand)

Synonymies are limited to key citations and illustrations. All illustrated specimens are shown at the same scale. Many species descriptions are based on the P_1_ element only. All figured specimens are located and stored in the Paleontological Institute and Museum of the University of Zurich (PIMUZ), Karl-Schmid-Strasse 4, 8006 Zürich, Switzerland. Suprageneric classification is following mostly Donoghue et al. (2008) and Orchard (2005). The terminology for the orientation of the element is the traditional one based on the orientation and curvature of the cusp. It only refers to the element itself and not its natural orientation within the animal (Purnell et al., 2000). ‘Lower’ refers to the side of the element from which the basal cavity opens. ‘Upper’ refers to the opposite side. The term ‘cusp’ refers only to the cusp sensu stricto (Klapper & Philip, 1972). For the figures, the orientation is standardized with the anterior side to the top, and the denticles tips to the left in ‘lateral’ views. For each element, the order of views from left to right is ‘lateral’, ‘oral’ and ‘aboral’ views, unless otherwise specified.

Class CONODONTA Eichenberg, 1930

Division PRIONIODONTIDA Dzik, 1976

Order OZARKODINIDA Dzik, 1976

Superfamily GONDOLELLOIDAE (Lindström, 1970)

Family GONDOLELLIDEA Lindström, 1970

Subfamily GLADIGONDOLELLINAE Hirsch, 1994

Genus GLADIGONDOLELLA Müller, 1962

1968 *Dichodella* Mosher, p. 923

*Type species. Polygnathus tethydis* Huckriede, 1958, p. 157–158, pl. 2, fig. 38a–b.

*Type stratum and locality. Trachyceras austriacum* bed (Julian), Feuerkogel near Röthelstein, Austria.

*Remarks*. This genus was first introduced by Müller (1962) for a Middle Triassic carminiplanate P_1_ element with a relatively short posterior process and corresponding keel posteriorly of the pit, a narrow platform and a low carina. As the type species may have been significantly different from some Spathian forms, the phylogenetic relationships of *Gladigondolella* are uncertain but the rootstock might lie within the evolution from *Borinella* (Orchard, 2007).

*Gladigondolella laii* Chen et al. morphotype A

Fig. 13B, C, G?

**Fig. 13:**
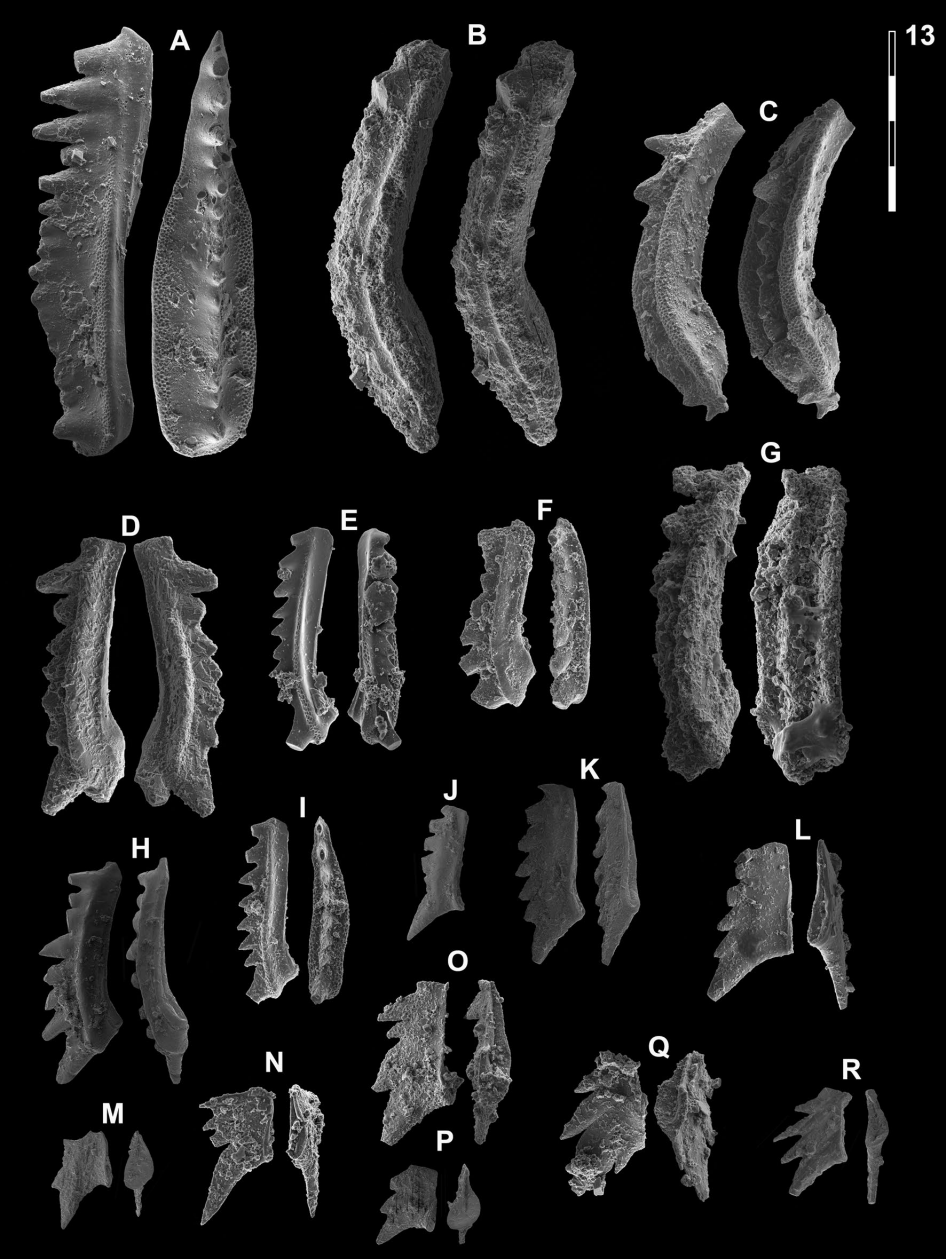
Gladigondolellinae, Neogondolellinae and Cornudininae from Qiakong, Laren, Shanggang and Lilong. Magnification is × 80. The scale bar is 400 μm. All elements are considered to be P_1_ elements if not specifically identified otherwise. **A** *Borinella buurensis* (Dagis), LAR212, PIMUZ 39103. **B**,** C**, **G** *Gladigondolella laii* (morphotype A); **B** LAR231C, PIMUZ 39132; **C** BAN2, PIMUZ 39133; **G** LAR227C, PIMUZ 39134. **D**, *sp. indet*. (P_2_ element?), SHA319, PIMUZ 39293. **E**,** F**,** H**,** I** *Gladigondolella laii* (morphotype B); **E** LIL510, PIMUZ 39135; **F** LIL512, PIMUZ 39136; **H** QIA140, PIMUZ 39137; **I** LIL510, PIMUZ 39138. **J–L**, **O**, *Spathicuspus spathi* (Sweet); **J** QIA141, PIMUZ 39300; **K** QIA144, PIMUZ 39301; **L** BAN1, PIMUZ 39302; **O** SHA322, PIMUZ 39303. **M**, **P** *Spathicuspus* n. sp. A; **M** QIA144, PIMUZ 39297; **P**, QIA143, PIMUZ 39298. **N**, **R** *Spathicuspus spathi* (juvenile) (Sweet); **N**, LIL515D, PIMUZ 39304; **R** QIA143, PIMUZ 39305. **Q** *Spathicuspus* n. sp. B; LAR226C, PIMUZ 39299

2013 *Neogondolella* sp.; Yan et al., fig. 6, nr. X.

2015 *Neogondolella*? n. sp. A; Chen et al., figs. 9.5–9.8.

2019 *Neogondolella*? n. sp. A; Chen et al., figs. 6.8, 6.9.

2021 *Gladigondolella laii* sp. nov.; Chen et al., figs. 4.20, 4.21 (only).

*Material*. ca. 15 specimens.

*Diagnosis.* Segminiplanate P_1_ element. Platform is arched, thickened and the platform margins are oriented orally. Denticles slightly fused at mid-length, larger and more discrete towards the anterior end.

*Description.* The element is arched in lateral view. In oral view, the margins of the thickened platform are subparallel at mid-length, and taper anteriorly. The platform is V-shaped, and the margin is directed orally. The cusp may be conspicuous in well preserved specimens. A posterior process may be present, which bears additional posterior denticles, otherwise the posterior margin is rounded. The carina denticles are partly fused at mid-length, taller and more discrete towards the anterior end.

*Remarks. Gladigondolella laii* differs from *Borinella* by having a more fused carina and an arched and thick platform whose margin are oriented orally. In comparison with the type species of *Gladigondolella*, a Middle Triassic species, the posterior process is much less developed and less arcuated in the carinal axis. Chen et al. (2015) rejected the assignment of their *Ng*. n. sp. A to *Gladigondolella* on the basis that posterior denticles are never present behind the cusp. In our material (e.g. Fig. 13C) small denticles are present occasionally. With the exception of these additional posterior denticles, our specimens resemble those of Chen et al. (2015) very closely, hence they are synonymized here. Implicitly we assume there is variation within the species, a small posterior process being present or not. This taxon is thought to be intermediary between *Borinella* and *Gladigondolella*, but through the shared derived characteristic of the posterior cusp we determine it as the former genus*.* Chen et al., (2019; figs. 6.8, 6.9) illustrated similar and coeval (associated with *Ic. collinsoni*) elements from Oman, which they assigned to *Neogondolella* n. sp. A. Their illustrated specimens more closely resemble *Gladigondolella tethydis* (Huckriede) and were later assigned to a new species of *Gladigondolella*: *Gladigondolella laii* (Chen et al., 2021). To date *Gladigondolella* is mostly known from the Middle Triassic (e.g. Goudemand et al., 2012a; Orchard et al., 2007a, 2007b). If, as it seems, the here illustrated specimens and/or those from Chen et al., (2019, 2021) do indeed belong to *Gladigondolella*, then they represent their oldest known representatives of this genus so far. A few unpublished specimens from middle Smithian rocks of Oman may also belong to this genus (Leu et al., submitted; L. Dudit, pers. comm.), which further question the origin and age of *Gladigondolella*. Its preference for deeper, colder habitats (Trotter et al., 2015) may then explain its spotty occurrences within the Early Triassic, preferably during relatively colder intervals and/or in deeper, colder refuge areas like Oman.

*Occurrence*. South China; *Ic. collinsoni* and *Tr. homeri* zone, Jiarong (Chen et al., 2015). Bed 54, Luolou formation, *Ic. collinsoni* and *Tr. homeri* zone, Bianyang section, Guizhou (Yan et al., 2013).

*Gladigondolella laii* Chen morphotype B

Fig. 13E, F, H, I

2015 *Neogondolella*? n. sp. B; Chen et al., figs. 9.9–9.10.

2019 *Neogondolella*? n. sp. A Chen; Liu et al., p. 14, pl. 4, fig. 19 (only).

2019 *Neogondolella*? n. sp. B Chen; Liu et al., p. 14, pl. 4, fig. 9.

2021 *Gladigondolella laii* sp. nov.; Chen et al., figs. 4.5, 4.22, 4.25, 4.26 (only).

*Material*. ca. 20 specimens.

*Diagnosis.* Segminiplanate P_1_ element, narrow platform tapers towards the anterior, denticles moderately discrete. Very large, distinct, terminal, strongly reclined cusp.

*Description.* The platform is narrow, subparallel, slightly upturned and tapers towards the anterior end. The posterior margin is rounded in a narrow platform brim around the large terminal cusp. The cusp is massive and inclined posteriorly. The carina is moderately high with pointed denticles which are almost uniform in height.

*Remarks.* The large terminal cusp differs from *Borinella* whose denticles are also higher and more discrete, but resembles that of *Scythogondolella*. Yet, *Gl. laii* morphotype B lacks a prominent rounded basal loop surrounding a small pit, a distinctive feature of *Scythogondolella*. Chen et al. (2015) suggests that their *Ng*. n. sp. B could be a juvenile stage of *Ng*. n. sp. A (reassigned here and in Chen et al. (2021) to *Gladigondolella laii* see above) based on size considerations and the fact that they are both found in the same interval. Yet, they were not found in the same samples and the small forms (*Gl. laii* morphotype B) lack a posterior process, although the juvenile forms of ‘true’ gladigondolellids usually bear a posterior process. Hence, we tentatively keep them as separate morphotypes. Nevertheless, in Chen et al. (2021), both forms are found in the same sample (e.g. figs. 4.23 and 4.24) and can therefore be considered as different growth stages.

*Occurrence*. *Ic. collinsoni* and *Tr. homeri* zone, Luolou formation, Jiarong, Guizhou, South China (Chen et al., 2015).

Subfamily NEOGONDOLELLINAE Hirsch, 1994

Genus BORINELLA Budurov & Sudar 1994

1988 *Pseudogondolella* Kozur, p. 244.

1993 *Kozurella* Budurov & Sudar, p. 24.

*1994 (June) *Borinella* Budurov & Sudar, p. 30.

1994 (September) *Chengyuania* Kozur, pp. 529–530.

*Type species. Neogondolella buurensis* Dagis, 1984.

*Type stratum and locality.* Buur River Basin, Northern middle Siberia, Early Triassic *Hedenstroemia* Zone, Russia.

*Remarks.* The multi-element apparatus of this genus appears to be essentially the same as that of *Neogondolella* (Orchard, 2008). The P_1_ elements of the species of this genus all have in common discrete blade-carinal denticles that lengthen towards the anterior.

*Borinella buurensis* (Dagis, 1984)

Fig. 13A

1978 *Neogondolella jubata* Sweet; Weitschat & Lehmann, p. 98, pl. 13, figs. 1–6.

*1984 *Neogondolella buurensis* n. sp.; Dagis, p. 12, pl. 2, figs. 6–15, pl. 3, fig. 1–2, pl. 11, figs. 1–4, pl. 12, figs. 1, 2, pl. 16, figs. 1–4.

**Fig. 14:**
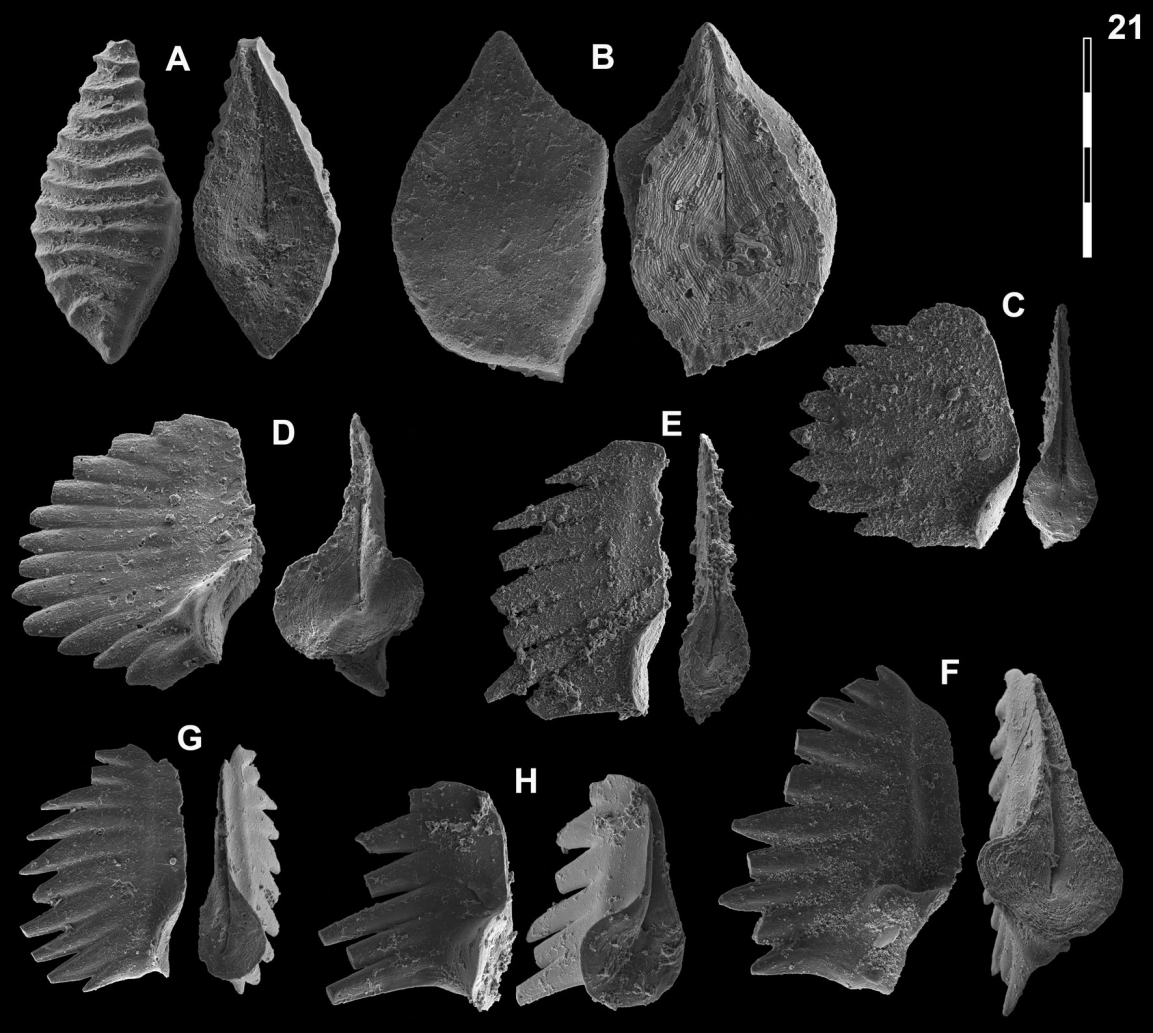
Neogondolellinae, Novispathodinae and uncertain from Shanggang*.* Magnification is × 80. The scale bar is 400 μm. All elements are considered to be P_1_ elements if not specifically identified otherwise. **A**, *Eurygnathodus costatus* (Staesche); SHA325, PIMUZ 39130. **B** *Eurygnathodus hamadai* (Koike); SHA326, PIMUZ 39131. **C**, **D**, **F**, **G** *Novispathodus* ex gr. *waageni* (Sweet); **C** SHA328, PIMUZ 39238; **D** SHA328, PIMUZ 39239; **F** SHA325, PIMUZ 39240; **G** SHA326, PIMUZ 39241. **E** *Neospathodus* ex gr. *cristagalli* (Huckriede); SHA330, PIMUZ 39182. **H** *Neospathodus dieneri* (Sweet); SHA338, PIMUZ 39181

1984 *Neogondolella elongata* Sweet; Hatleberg & Clark, Pl. 1, fig. 14 (only).

2005 *Neogondolella* aff. *sweeti* Kozur & Mostler; Zhao, p. 131, pl. 13, fig. 1.

2007 *Borinella buurensis* (Dagis); Orchard, p. 113, pl. 1, nr. 7, 15, 27.

2008 *Borinella buurensis* (Dagis); Nakrem et al., p. 528, fig. 4.19.

2008 *Borinella* aff. *buurensis* (Dagis); Nakrem et al., p. 528, fig. 4.17–4.18.

2008 *Borinella buurensis* (Dagis); Orchard, p. 400, figs. 5.9–5.13.

2012a *Borinella* aff. *buurensis* (Dagis); Goudemand & Orchard in Goudemand et al., p. 1032, figs. 2AA.

2015 *Borinella* aff. *buurensis* (Dagis); Chen et al., fig. 9.11.

2018 *Borinella* aff. *buurensis* (Dagis); Maekawa in Maekawa et al., p. 45, fig. 29.27.

2019 *Neogondolella* ex. gr. *jakutensis* Dagis; Chen et al., figs. 4.3, 4.5 (only).

2019 *Borinella* aff. *buurensis* (Dagis); Liu et al., p. 13, pl. 3, fig. 10 (only).

2021 *Borinella* aff. *buurensis* (Dagys); Chen et al., fig. 5.9 (only).

*Number of specimens*. 5

*Description*. Subsymmetrical segminiplanate P_1_ element whose platform extends as a brim around the posterior edge, but not to the anteriormost quarter of the unit. In the posterior half, the platform margins are subparallel in oral view. The cusp varies in height and may be conspicuous. A smaller posterior denticle may be present in some specimens, in which case, it tends to be offset from the main axis. As in any *Borinella* species, the denticles become gradually more discrete and taller towards the anterior end. As illustrated by the figured specimen, whose denticles at mid-length are almost as high as the anteriormost ones, this denticulation gradient may be variable.

*Remarks*. Orchard (2007) suggested that the cusp of the P_1_ of *B. buurensis* is typically weak, contrary to that of *B. sweeti*. Our collections suggest instead it is variable, as illustrated here. In our view it is difficult to differentiate *B. sweeti* and *B. buurensis*.

*Occurrence.* South China; *Nv. pingdingshanensis* Zone, Tsoteng section (Goudemand et al., 2012b), Jiarong section, Guizhou (Chen et al., 2015): Southwest Japan (Maekawa et al., 2018): Boreal region; *Hedenstroemia* zone, Siberia (Dagis, 1984), *Tardus* zone, Canadian Arctic (Orchard, 2008), Spitsbergen (Nakrem et al., 2008; Weitschat & Lehmann, 1978).

Genus NEOSPATHODUS Mosher, 1968

*Type species and holotype*. *Spathognathodus cristagalli* Huckriede, 1958, pp. 161–162, pl. 10, fig. 15.

*Type stratum and locality*. Lower Ceratite Limestone (LCL), Mittiwali near Chhidru, Salt Range, Pakistan.

*Neospathodus dieneri* Sweet, 1970

Fig. 14H

*1970 *Neospathodus dieneri* n. sp.; Sweet, pp. 249–251, pl. 1, figs. 1, 4.

1982 *Neospathodus dieneri* Sweet; Matsuda 1982, p. 90, pl. 2, figs. 1–11.

1982 *Neospathodus dieneri* Sweet; Koike, p. 37, pl. 6, figs. 15–21.

1984 *Neospathodus dieneri* Sweet; Dagis, p. 27, pl. 6, figs. 4–7.

1991 *Neospathodus dieneri* Sweet; Beyers & Orchard, pl. 5, fig. 4.

2007 *Neospathodus dieneri* Sweet; Orchard & Krystyn, p. 33, figs. 3, 6, 7.

2007 *Neospathodus dieneri* Sweet; Zhao & Orchard in Zhao et al., p. 35, pl. 1, figs. 9A–C, 12A–B.

2009 *Neospathodus dieneri* Sweet; Igo in Shigeta et al., p. 186, figs. 151.6–151.16, 152.8 (only).

2009 *Neospathodus dieneri* Sweet; Orchard & Zonneveld., pp. 782–784, fig. 14, parts 1–4.

2016 *Neospathodus dieneri* Sweet; Maekawa et al., p. 199, figs. 4.8–4.10.

2018 *Neospathodus dieneri* Sweet; Maekawa in Maekawa et al., pp. 25–28, figs. 15.8–15.9, 15.12–15.14, 15.18–15.19, 15.21–15.28 (only).

*Material.* ca. 40 specimens.

*Diagnosis.* See Sweet (1970), pp. 249–251. The basal cavity is symmetrically rounded with a circular outline. The posterior margin is upturned. The denticles are round (subcircular in cross-section) with a pointy end, discrete and slightly recurved posteriorly. Basal groove runs from the basal pit to the anterior end.

*Remarks.* Zhao et al. (2007) distinguished three morphotypes of *Neospathodus dieneri* on the basis of the length of the terminal cusp relative to the other denticles. Their illustrated morphotype 3 (Zhao et al., 2007, Fig. 11A–C) however does not, in our opinion, belongs to *N*. ex gr. *dieneri* but is more closely related to *N. cristagalli* on the basis of its laterally flattened denticles and S-shaped posterior margin. Maekawa and Igo (2014, in Shigeta et al., 2014) assigned specimens to *N. dieneri* which is laterally flattened, blade-shaped denticles look very different from the holotype. They may instead belong to another species or even to another genus (possibly to *Discretella*?).

*Occurrence*. This species has been reported worldwide from the Dienerian and early Smithian. This includes South China (Zhao et al., 2007 and this study), Malaysia (Koike, 1982), the Northern Indian margin (Matsuda, 1982; Sweet, 1970), South Primorye in Russia (Shigeta et al., 2009), Canada (Beyers & Orchard, 1991) and Japan (Maekawa et al., 2018).

*Neospathodus* ex gr. *cristagalli* Huckriede, 1958

Fig. 14E

1970 *Neospathodus cristagalli* Huckriede; Sweet, p. 346, pl. 1, figs. 14, 15.

1982 *Neospathodus cristagalli* Huckriede; Matsuda p. 92, pl. 3, figs. 1–12.

2005 *Neospathodus* cf. *cristagalli* Sweet; Orchard, p. 89, text-fig. 14.

2014 *Neospathodus cristagalli* Huckriede; Maekawa & Igo in Shigeta et al., p. 223, figs. 161.10–161.12.

2015 *Neospathodus cristagalli* Huckriede; Maekawa in Maekawa et al., p. 315, figs. 5.4–5.6.

*Material.* ca. 30 specimens.

**Fig. 15:**
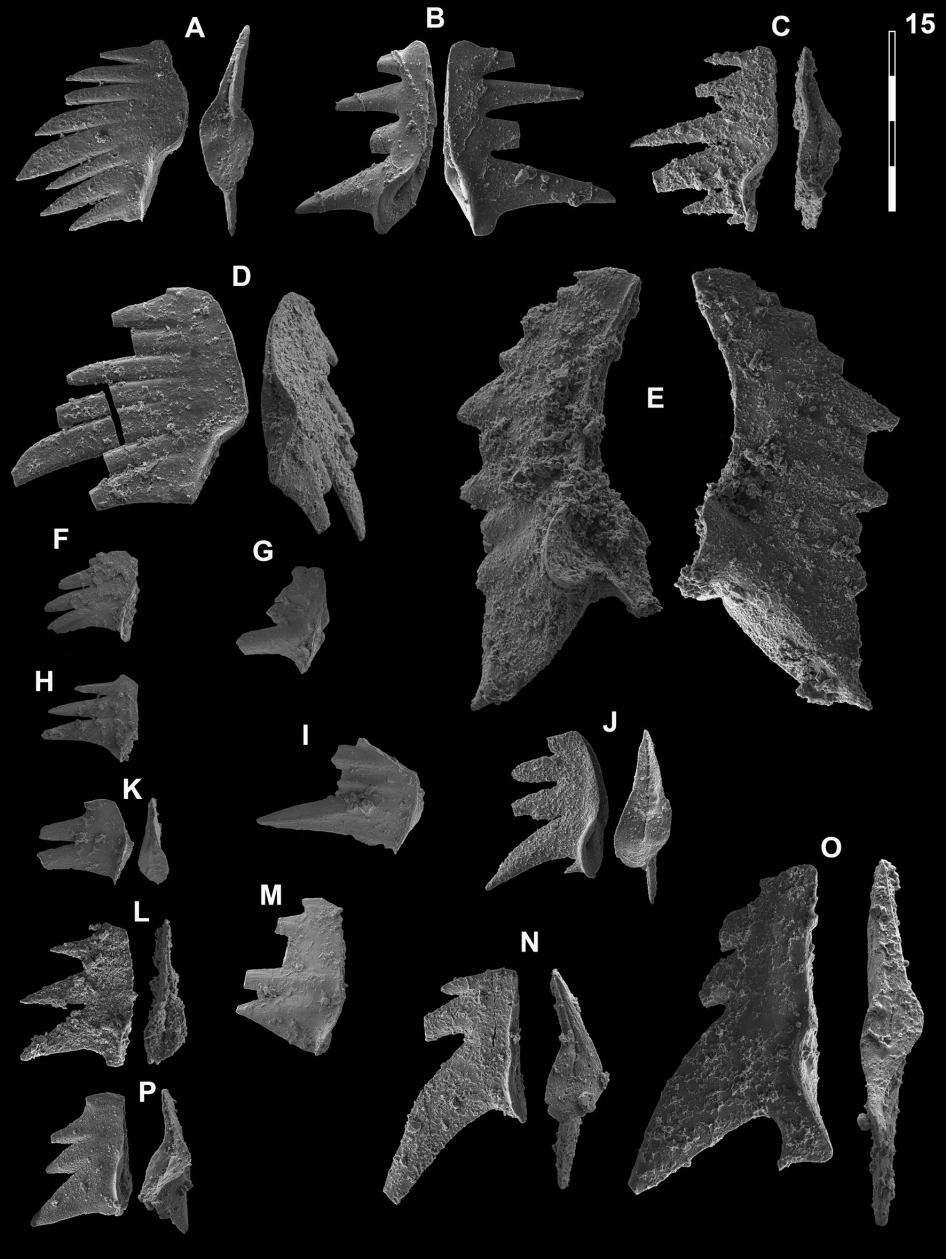
Neogondolellinae and Mullerinae from Qiakong, Laren and Shanggang*.* Magnification is × 80. The scale bar is 400 μm. All elements are considered to be P_1_ elements if not specifically identified otherwise. **A**, **D** *Neospathodus bevelledi* n. sp.; **A** SHA333, PIMUZ 39176; **D**, SHA332, PIMUZ 39177. **B**, **J** *Discretella* aff. *discreta* (Müller); **B** SHA304, PIMUZ 39104; **J** SHA334, PIMUZ 39105. **C**, **L** *Discretella discreta* (Müller); **C** SHA334, PIMUZ 39107; **L** SHA332, PIMUZ 39108. **E** *Guangxidella bransoni* (Müller); SHA304, PIMUZ 39139. **F**, **H**, **K**, **M**, **P** *Discretella pseudodieneri* n. sp.; **F** QIA124, PIMUZ 39120; **H** QIA124, PIMUZ SQL54990; **K** QIA121, PIMUZ 39121; **M** QIA120, PIMUZ 39122; **P** LAR232, PIMUZ 39123. **G** *Discretella* cf. *discreta* (Müller); QIA120, PIMUZ 39109. **I** sp. indet.; QIA120, PIMUZ 39294. **N** *Discretella*? n. sp. B; LAR232, PIMUZ 39112. **O** *Discretella*? n. sp. C; SHA342, PIMUZ 39114

*Remarks.* In comparison with the holotype of *N. cristagalli* (Huckriede, 1958, Pl. 10, fig. 15), this element is much shorter, the basal cavity is oval, posteriorly rounded and only partly inverted, and the posterior triangular cusp is not conspicuously separated from the other denticles. Sweet still included such forms within *N. cristagalli*, but excluded similar forms with a rounded basal cavity and rounded, pointy denticles and assigned them to the then new species *N. dieneri*. In our opinion, such short elements where the cusp is not separated from the other denticles would deserve to be differentiated as a new species.

*Occurrence*. *N. cristagalli* is known worldwide in the Dienerian and earliest Smithian. It is not clear yet whether the present form is younger than *N. cristagalli* sensu stricto and occurs only close to the Dienerian–Smithian (Induan–Olenekian) boundary: Toad Formation, British Columbia (Orchard, 2005). Salt Range, Pakistan (Sweet, 1970). Guryul Ravine, Kashmir (Matsuda, 1982). Nanpanjiang basin; north-eastern Vietnam *Flemingites* beds within the *Novispathodus* ex. gr. *waageni* Zone (Maekawa et al., 2015; Shigeta et al., 2014). Luolou Formation, *Flemingites* limestone (this study).

*Neospathodus bevelledi* n. sp*.*

**Fig. 16:**
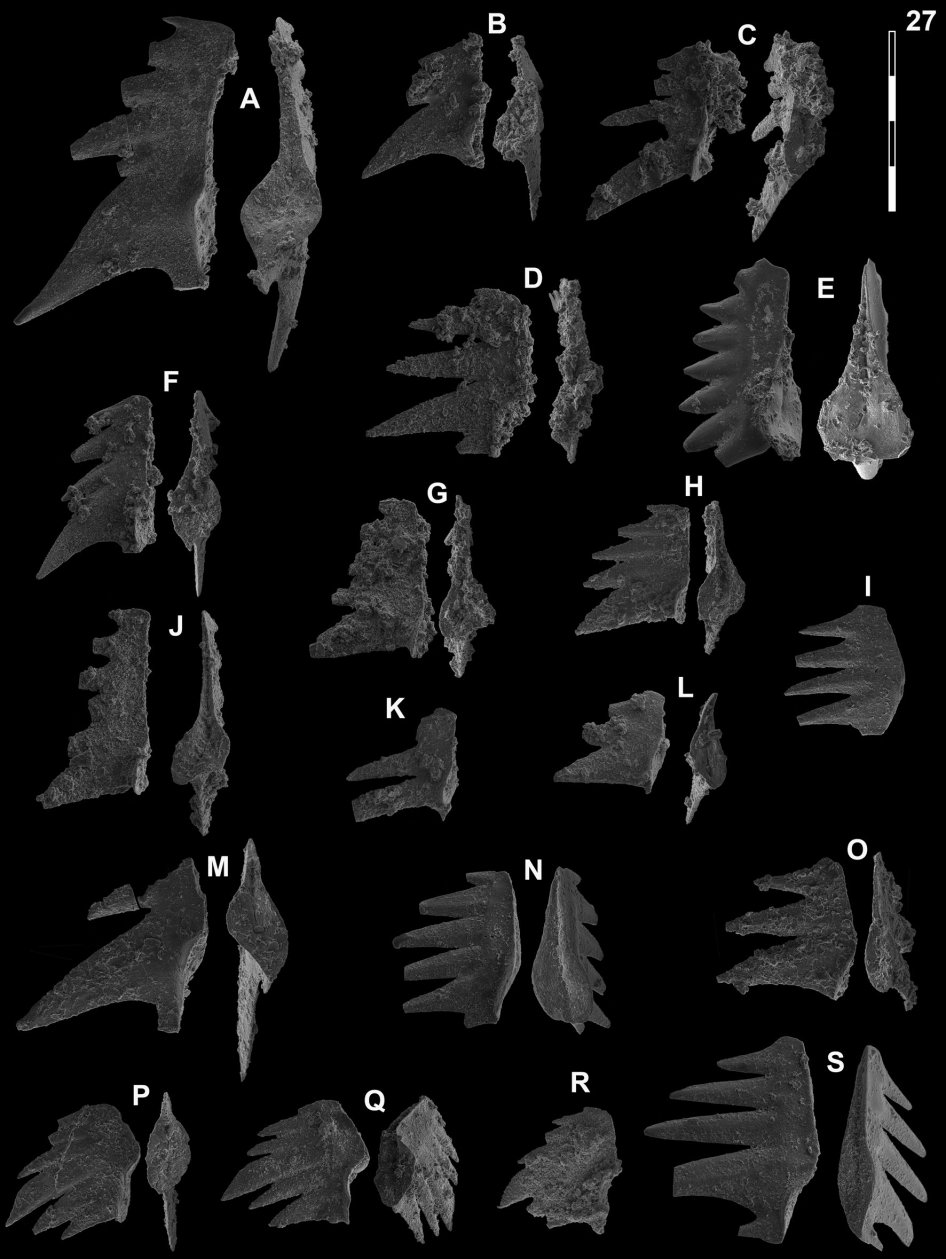
Neogondolellinae, Novispathodinae and Mullerinae from Shanggang and Youping cascade. Magnification is × 80. The scale bar is 400 μm. All elements are considered to be P_1_ elements if not specifically identified otherwise. **A**, **F**, **M** *Discretella?* n. sp. C; **A** YC16, PIMUZ 39115; **F** YC16, PIMUZ 39116; **M** SHA342C, PIMUZ 39117. **B** *Discretella?* n. sp. B; YC16, PIMUZ 39113. **C** *Discretella* aff. *discreta* (Müller); YC22, PIMUZ 39106. **D**, **S** *Discretella discreta* (Müller); **D** YC16, PIMUZ 39110; **S** SHA304C, PIMUZ 39111. **E** *Novispathodus* ex gr*. abruptus* (Orchard); YC41, PIMUZ 39225. **G–I**, **K**, **L**, **N**, **O** *Discretella pseudodieneri* n. sp.; **G** YC16, PIMUZ 39124; **H** YC25, PIMUZ 39125; **I** SHA304C, PIMUZ 39126; **K** YC22, PIMUZ 39127, **L** YC25, PIMUZ 39128; **N** SHA304C, PIMUZ 39129; **O** SHA343C, PIMUZ SQL54979. **J** *Guangxidella bransoni* (Müller); YC16, PIMUZ 39140. **P**–**R** *Neospathodus bevelledi* n. sp.; **P** SHA 304C, PIMUZ 39178; **Q** SHA333C, PIMUZ 39179; **R** YC12, PIMUZ 39180

Figs. 15A, D, 16P–R.

1990 *Neospathodus pamirensis* n. sp.; Dagis, p. 79–80, pl. 4, fig. 8, pl. 6, fig. 9 (only).

2007 *Neospathodus* n. sp. V Orchard; Orchard, p. 96, fig. 2.

2013 *Neospathodus cristagalli* Huckriede; Yan et al., p. 516, fig. 6, I–L.

2014 *Neospathodus spitiensis* Goel; Maekawa & Igo in Shigeta et al., p. 233–236, figs. 167.17–167.30, 168.7–168.9, 168.16–168.27, 168.34–168.36, 169.4–169.9, 169.21–169.32, 170.1–3.

2014 *Neospathodus* sp. indet A Maekawa & Igo; Shigeta et al., p. 179, fig. 170.10–170.33.

2014 *Novispathodus* ex gr. *waageni* Sweet; Maekawa & Igo in Shigeta et al., p. 244, figs. 179.4–179.6, 179.13–179.15.

2015 *Neospathodus cristagalli* Huckriede; Chen et al., p. 112, fig. 8.12–13, 8.15.

2016 *Neospathodus* sp. indet A; Maekawa et al. p. 200, figs. 5.2–5.7.

2018 *Neospathodus cristagalli* Huckriede; Maekawa in Maekawa et al., p.22, fig. 14.24 (only).

?2019a *Novispathodus pingdingshanensis* Zhao & Orchard; Wu et al. figs. 32–34.

2021 *Novispathodus waageni* (Sweet); Sun et al., fig. 5.22 (only).

*Etymology*: named after the Latin (and British English) word ‘bevelled’, which refers to the wedge-like cessation of the denticles of the P_1_ element.

*Holotype:* specimen illustrated in Fig. 15A

*Paratype:* specimen illustrated in Fig. 15D

*Type locality:* Shanggang road cut, Luolou formation, Guangxi Province, China.

*Type level*: Luolou Formation, within the early-to-middle Smithian limestones (*Owenites* beds), 2–3 m above *Flemingites* limestone.

*Material. *> 10 specimens.

*Diagnosis*. A species with a segminate-to-carminate P_1_ element with distinctively wedge-shaped, bevelled denticle tips. Cusp is either terminal or in front of one or two posterior denticles. The shallow basal cavity is posteriorly elongated and tapered. In lateral view the lower margin is straight or upturned anteriorly, conspicuously upturned at mid-length, in front of the cusp, and straight or upturned posteriorly. The denticle directly in front of the cusp and above the kick in the lower margin may get broader towards its wedge-shaped tip and is usually conspicuously broader than the other denticles.

*Description*. The P_1_ element is segminate to carminate (the posterior process bearing one or two denticles) and has a rigid cockscomb-like form. In lateral view, the lower margin is conspicuously bent or upturned at mid-length, the anterior and posterior parts of the lower margin making an angle of 15° to 40° and being often offset. The anterior lower margin is straight or slightly upturned, the posterior lower margin is usually more upturned. The upper margin is arcuate, with a peak in height slightly behind mid-length. The length-to-height ratio of the element is about 1–1.2:1. The moderately fused and laterally flattened denticles seem to radiate from a point that is located below the element, slightly anterior of the lower margin kick. The denticles are usually slightly recurved posteriorly. The cusp is located directly behind the lower margin kick and is as high as, or smaller than the up to 2 posterior denticles. The denticle directly in front of the cusp is usually conspicuously larger and broader than the other denticles. It may get broader towards its characteristically wedge-shaped tip. In lower/aboral view, the shallow basal cavity is subtriangular, being elongated and tapered posteriorly. The posterior end of the basal cavity can be either pointy or sub-rounded (as in the holotype). A groove runs from the basal cavity pit to the anterior end.

*Remarks.* Orchard (2007) was the first to differentiate such forms on the basis of their wedge-shaped denticle tips (his *Neospathodus* n. sp. V). The holotype of *Neospathodus pamirensis* Dagis (1990) appears to fall within the scope of *Ns*. *bevelledi* n. sp. but Dagis differentiated *Ns. pamirensis* on the basis of a “sharply angular lower margin” of the P_1_ element and “cut-off ends” of the denticles (our rough translation from Russian), which based on his illustrations seem to correspond to sharply *broken* denticles, not to the wedge-shaped natural end that we use as a diagnostic feature of *Ns*. *bevelledi* n. sp., a feature that cannot be observed in his holotype of *Ns. pamirensis.* Moreover, the “sharply angular lower margin” does not differentiate his elements from those of *Ns. spitiensis*. *Ns. bevelledi* n. sp. compares most to *Ns. cristagalli* and *Ns. spitiensis* but none of the latter display the mid-length located, broad, large and bevelled denticle that is characteristic of *Ns. bevelledi* n. sp. Furthermore, *Ns. spitiensis*, as defined and illustrated by Goel (1977) has a similarly extended basal cavity and hence a similar lower margin but a much larger length-to-height ratio of about 1.8:1, its denticles being essentially smaller and more reclined posteriorly than in *Ns. bevelledi* n. sp. Maekawa and Igo (in Shigeta et al., 2014) assigned similar elements from Vietnam either to *Ns. spitiensis* or to their ‘*Ns*. sp. indet. A’. They differentiated ‘*Ns*. sp. indet. A’ on the basis that the P_1_ elements are higher and display fewer denticles. In our view, most of the elements they assigned to *Ns. spitiensis* are already too high to belong to *Ns. spitiensis* and most of them display the characteristic bevelled denticles that none of the original *Ns. spitiensis* possessed. Hence, they probably belong to *Ns. bevelledi* n. sp. instead. Their material, however, shows a possible somewhat continuous transition between *Ns. spitiensis* Goel and *Ns. bevelledi* n. sp*.*, suggesting a close relationship between both taxa. In comparison with *Ns*. *cristagalli*, the P_1_ elements of *Ns*. *bevelledi* n. sp. are shorter and higher and although some elements display a posteriormost denticle that is triangular, it is neither smaller nor separated from the other denticles as is characteristic of Ns. *cristagalli*.

*Occurrence*. China: Bianyang and Jiarong sections, Nanpanjiang basin, Guizhou province (Chen et al., 2015; Yan et al., 2013); Japan: Taho Formation (Maekawa et al., 2018); Vietnam: Bac Thuy Formation, Smithian *Flemingites* to *Owenites* beds (Shigeta et al., 2014); Oman: Smithian age (Orchard, 2007).

Subfamily NOVISPATHODINAE Orchard, 2005

Genus NOVISPATHODUS Orchard, 2005

*Type species and holotype*. *Neospathodus abruptus* Orchard, 1995, pp. 118–119, figs. 3.23–24.

*Type stratum and locality*. Jabral Safra, Oman.

Remarks. The genus *Novispathodus* was introduced as a new genus with a 15-element apparatus by Orchard (2005) and revised by Goudemand et al. (2012b) based on the swapping of the S_1_ and S_2_ positions. Additional to the type species (*Nv. abruptus*) and other species whose multi-element apparatus has been reconstructed (e.g. *Nv. pingdingshanensis* (Goudemand et al., 2012b), *Nv. waageni* (unpublished)), we tentatively assign also the following species to *Novispathodus* on the basis that they co-occur with S and M elements that are reminiscent of *Novispathodus*.

*Novispathodus pingdingshanensis* (Zhao & Orchard, 2007)

**Fig. 17:**
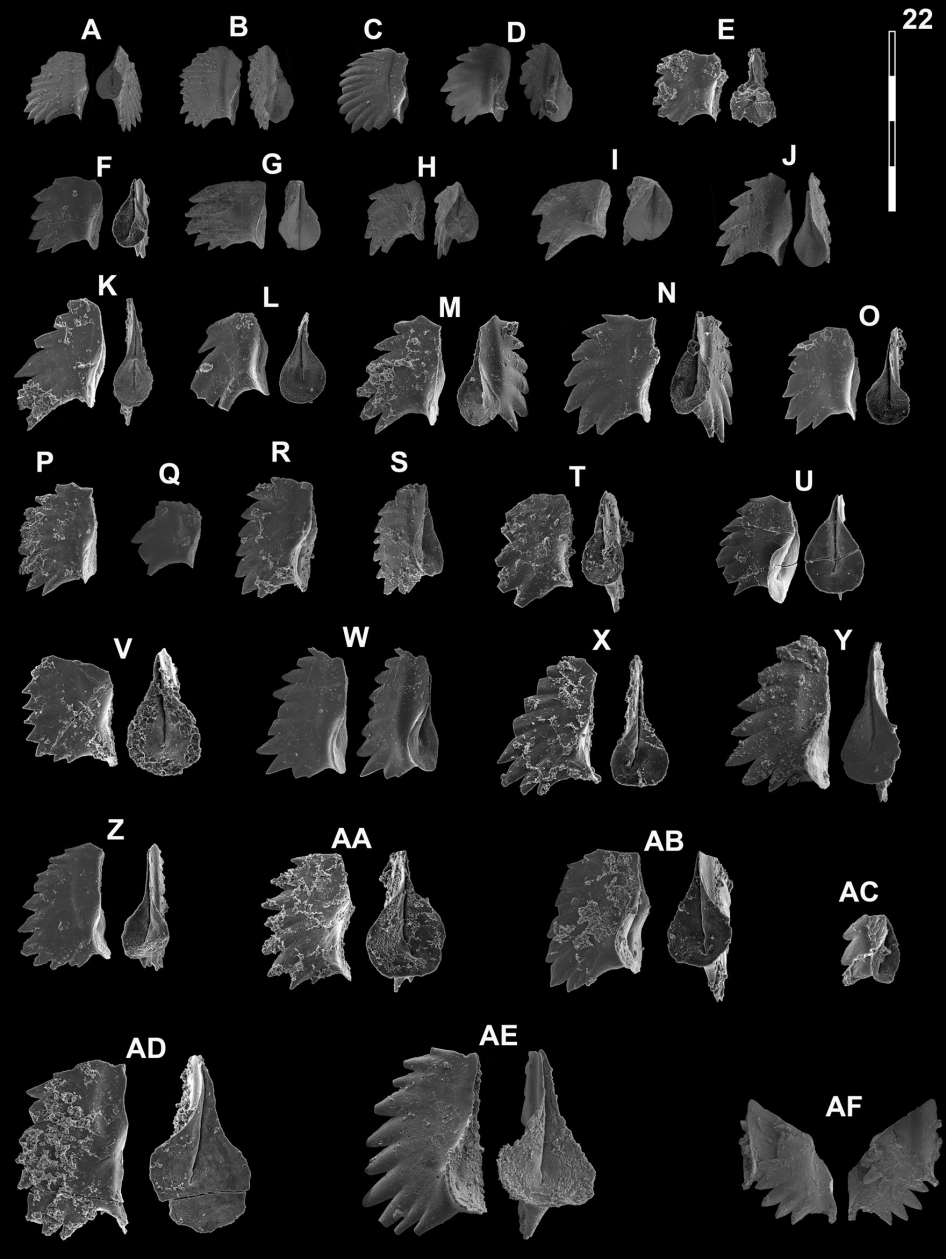
Novispathodinae from Qiakong, Laren, Shanggang, and Lilong*.* Magnification is × 80. The scale bar is 400 μm. All elements are considered to be P_1_ elements if not specifically identified otherwise. **A–C**, **J**, **T**, **AA** *Novispathodus* ex gr. *pingdingshanensis* (Zhao & Orchard); **A**, QIA138, PIMUZ 39226; **B** QIA138, PIMUZ 39227; **C** LAR202, PIMUZ 39228; **J** QIA136, PIMUZ 39229; **T** LIL504, PIMUZ 39230; **AA** LIL508, PIMUZ 39231. **D–F**, **M–P**, **R**, **S**, **U**, **X**, **AB**, **AD** *Novispathodus pingdingshanensis* (Zhao & Orchard); **D** QIA135, PIMUZ 39259; **E** LIL508, PIMUZ 39260; **F** LIL506, PIMUZ 39261; **M** LIL506, PIMUZ 39262; **N** LIL506, PIMUZ 39263; **O** LIL507, PIMUZ 39264; **P** LIL506, PIMUZ 39265; **R** LIL508, PIMUZ 39266; **S** LAR204, PIMUZ 39267; **U** LIL507, PIMUZ 39268; **X** LIL508, PIMUZ 39269; **AB** LIL508, PIMUZ 39270; **AD** LIL508, PIMUZ 39271. **G**, **I**, **Q** *Novispathodus* cf. ?*gryphus* n. sp.; **G** QIA136, PIMUZ 39204; **I** QIA136, PIMUZ 39205; **Q** QIA135, PIMUZ 39206. **H**, **K**, **L** *Novispathodus gryphus* n. sp.; **H** QIA135, PIMUZ 39243; **K** LIL506, PIMUZ 39244; **L** LIL506, PIMUZ 39245. **V** *Novispathodus praebrevissimus* n. sp.; LIL507, PIMUZ 39273. **W**, **Y**, **Z**, **AE** *Novispathodus* ex gr. *abruptus* (Orchard); **W** LIL505, PIMUZ 39208; **Y** SHA346, PIMUZ 39209; **Z** LIL506, PIMUZ 39210; **AE** SHA346, PIMUZ 39211. **AC** sp. indet.; LIL504, PIMUZ 39295. **AF** *Novispathodus pingdingshanensis* (P_1_ cluster) (Zhao & Orchard); QIA136, PIMUZ 39272

Fig. 17D–F; M–P, R, S, U, X, AA, AB, AD, AF

*2007 *Neospathodus pingdingshanensis* n. sp.; Zhao & Orchard, Zhao et al., p.36, pl. 1, fig. 4A–C.

2012a *Novispathodus pingdingshanensis* (Zhao & Orchard); Goudemand & Orchard in Goudemand et al., p. 1030–1031, figs. 2B, F, G, I–J, M, P, Q, AD, 3T-U, 6.

2013 *Neospathodus pingdingshanensis* Zhao & Orchard; Chen et al., p 825, fig. 3.10, 3.12.

2013 *Neospathodus waageni* subsp. nov. A; Metcalfe et al., p. 1144, figs. 9.1–9.5, 9.7, 9.8, 9.10.

2014 *Novispathodus pingdingshanensis* (Zhao & Orchard); Maekawa & Igo in Shigeta, p. 239–240, figs. 171.13–171.31.

2015 *Novispathodus pingdingshanensis* (Zhao & Orchard); Chen et al., p. 111, 112, figs. 7.1–7.4, 8.5, 8.6.

2016 *Novispathodus* ex. gr. *pingdingshanensis* (Zhao & Orchard); Komatsu et al., p. 69, figs. 5.4a–5.5c.

2016 *Neospathodus robustus* Koike; Chen & Kolar-Jurkovšek in Chen et al., p. 93, fig. 9.5 (only).

2018 *Novispathodus pingdingshanensis* (Zhao & Orchard); Maekawa in Maekawa et al., p. 36–37, figs. 20.2–20.18, 21.1–21.13.

2019 *Novispathodus pingdingshanensis* (Zhao & Orchard); Chen et al., fig. 3, nr. 8.

2019 *Novispathodus pingdingshanensis* (Zhao & Orchard); Liu et al., p. 13, pl. 3 fig. 5 (only).

*Material.* > 100 specimens.

*Diagnosis* (Zhao and Orchard, in Zhao et al., 2007; emended by Goudemand, in Goudemand et al., 2012b). Small segminate P_1_ elements characterized by a length/height ratio in the range of 1.32–2.34, and about 4–9 robust, wide, mostly fused, and distinctively posteriorly recurved denticles. In lateral view, the basal margin is usually straight. A large, broadly expanded oval to sub-rounded basal cavity is upturned on the inner margin and flat to downturned on the outer margin.

**Fig. 18:**
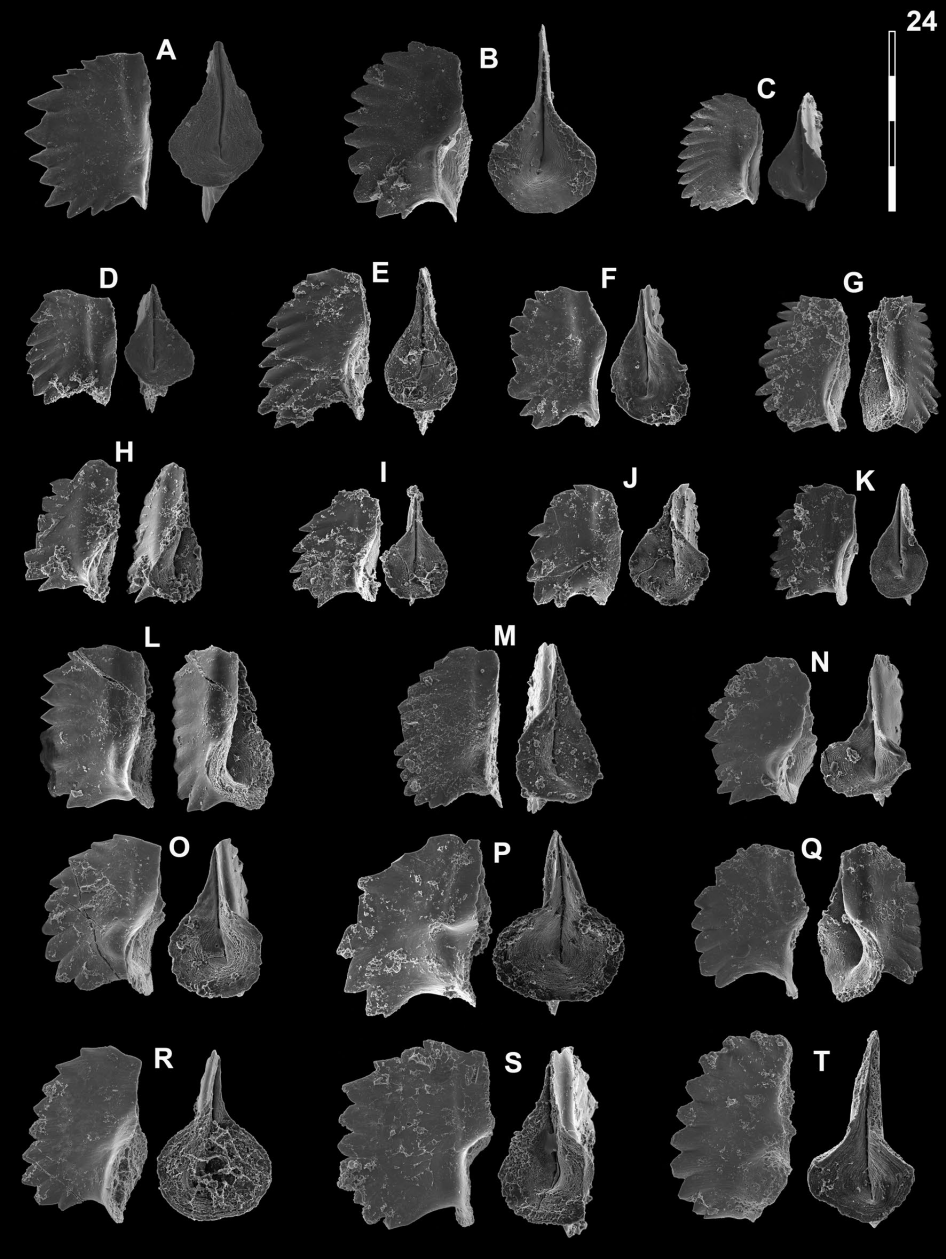
Novispathodinae from Laren and Lilong. Magnification is × 80. The scale bar is 400 μm. All elements are considered to be P_1_ elements if not specifically identified otherwise. **A**, **B**, **D**, **F**, **L**–**N**, **Q**, **S**, **T** *Novispathodus praebrevissimus* n. sp.; **A** LIL506, PIMUZ 39274; **B** LAR207, PIMUZ 39275; **D** LIL508, PIMUZ 39276; **F** LIL507, PIMUZ 39277; **L** LIL507, PIMUZ 39278; **M** LIL509, PIMUZ 39279; **N** LIL507, PIMUZ 39280; **Q** LIL507, PIMUZ 39281; **S** LIL507, PIMUZ 39282; **T** LIL507, PIMUZ 39283. **C**, **O**, **R** *Novispathodus* ?*praebrevissimus* n. sp.; **C** LAR204, PIMUZ 39284; **O** LIL507, PIMUZ 39285; **R** LIL507, PIMUZ 39286. **E**, **I** *Novispathodus* ex gr. *pingdingshanensis* (Zhao & Orchard); **E** LIL507, PIMUZ 39232, **I** LIL507, PIMUZ 39233. **G** *Triassospathodus* aff. *symmetricus* (Orchard); LIL507, PIMUZ 39309. **H** *Novispathodus* ex gr. *abruptus* (Orchard); LIL507, PIMUZ 39212. **J**, **K** *Novispathodus praebrevissimus* (juvenile) n. sp.; **J** LIL507, PIMUZ 39287; **K** LIL507, PIMUZ 39288. **P** *Novispathodus robustispinus* (Zhao & Orchard); LIL507, PIMUZ 39290

**Fig. 19:**
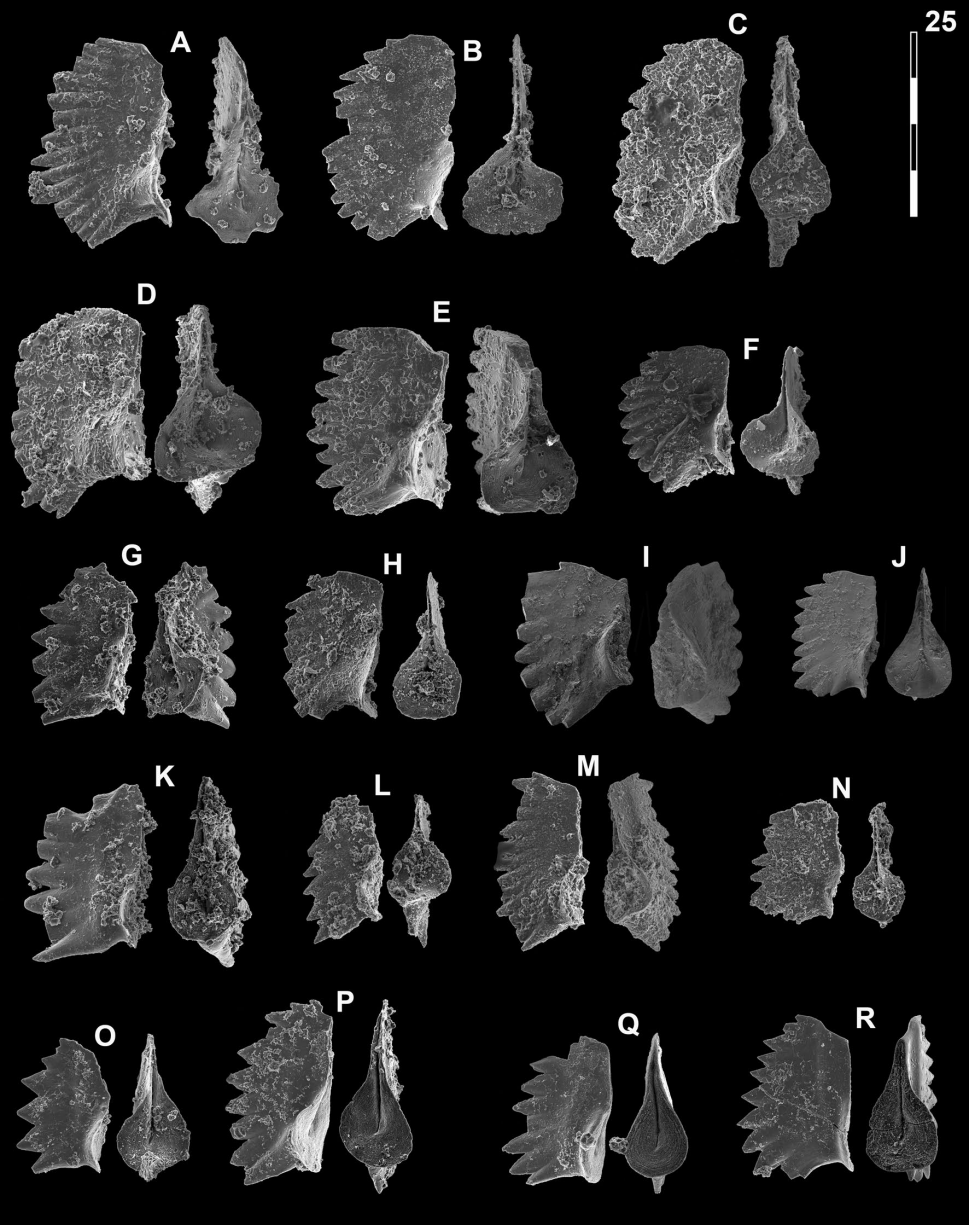
Novispathodinae from Qiakong, Laren and Lilong. Magnification is × 80. The scale bar is 400 μm. All elements are considered to be P_1_ elements if not specifically identified otherwise. **A** *Novispathodus* cf. *brevissimus* (Orchard); LIL515C, PIMUZ 39199. **B**, **F** *Novispathodus brevissimus* (Orchard); **B** LIL515B, PIMUZ 39200; **F** BAN1, PIMUZ 39201. **C** *Triassospathodus homeri* (Bender); LIL515D, PIMUZ 39317. **D**, **H** *Novispathodus* ?*brevissimus* (Orchard); **D** LIL515D, PIMUZ 39202; **H** LIL514A, PIMUZ 39203. **E** *Triassospathodus* cf. *homeri* (Bender); LIL515C, PIMUZ 39318. **G**, **K**, **O**–**Q** *Novispathodus* n. sp. A; **G** LIL504, PIMUZ 39251; **K** LIL504, PIMUZ 39252; **O** LIL505, PIMUZ 39253; **P** LIL505, PIMUZ 39254; **Q** LIL505, PIMUZ 39255. **I**, **L**, **M**, *Triassospathodus symmetricus* (Orchard); **I** QIA141, PIMUZ 39336; **L** LIL512, PIMUZ 39337; **M** LIL514A, PIMUZ 39338. **J** *Novispathodus* ex gr. *pingdingshanensis* (Zhao & Orchard); QIA138, PIMUZ 39237. **N** *Triassospathodus* cf. *symmetricus*; LIL514B, PIMUZ 39339. **R** *Novispathodus praebrevissimus* n.sp; LIL505, PIMUZ 39289

**Fig. 20:**
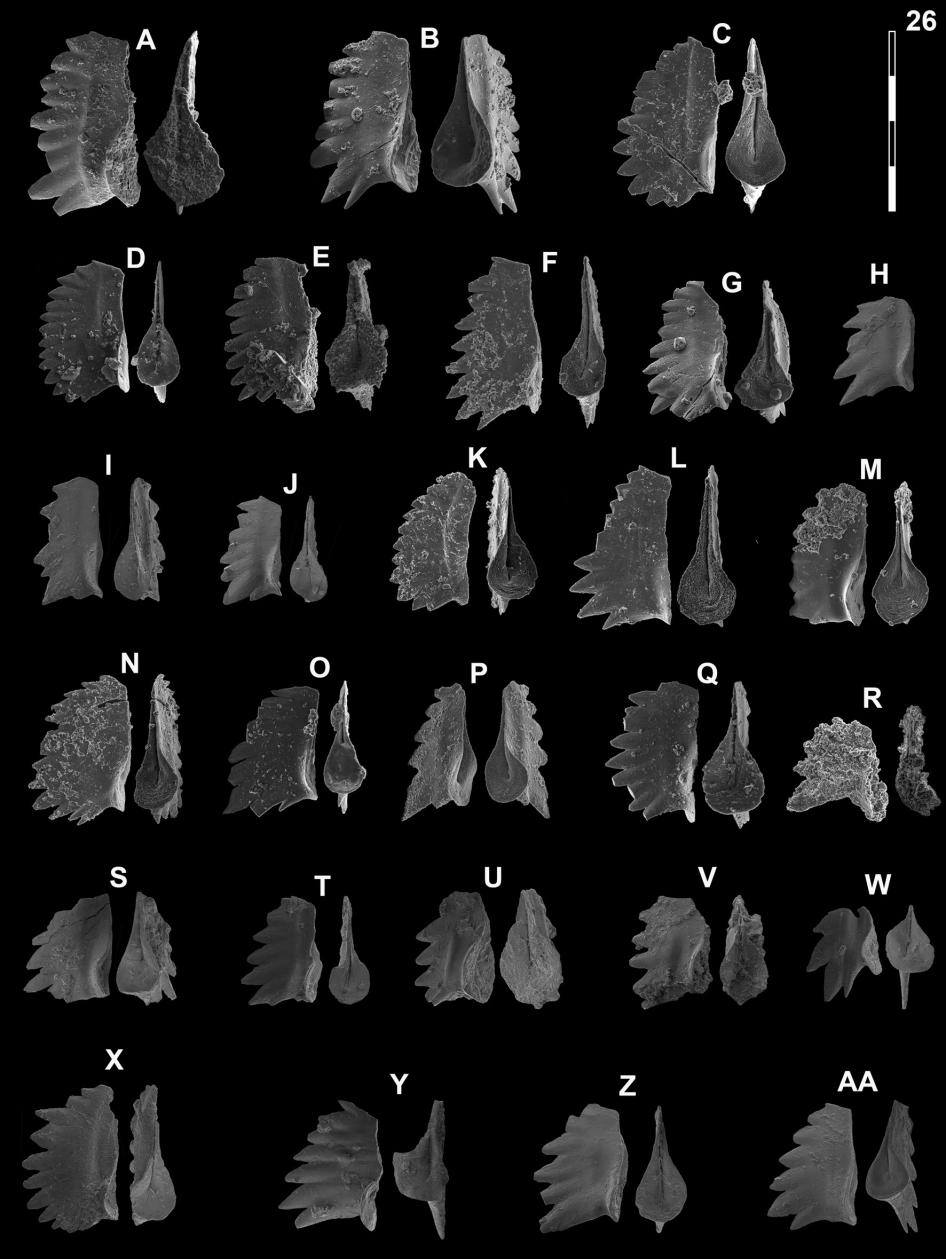
Novispathodinae from Qiakong, Laren and Lilong. Magnification is × 80. The scale bar is 400 μm. All elements are considered to be P_1_ elements if not specifically identified otherwise. **A**–**G**, **K**–**M**, **Q**, **X** *Novispathodus* ex gr. *abruptus* (Orchard); **A** LAR212, PIMUZ 39213; **B** LAR207, PIMUZ 39214; **C** LAR205, PIMUZ 39215; **D** LAR202, PIMUZ 39216; **E** LAR202, PIMUZ 39217; **F** LIL506, PIMUZ 39218; **G** LAR210, PIMUZ 39219; **K** LIL505, PIMUZ 39220; **L** LIL506, PIMUZ 39221; **M** LIL505, PIMUZ 39222; **Q** LAR203, PIMUZ 39223; **X** QIA136, PIMUZ 39224. **H**, **V** *Novispathodus* sp. indet.; **H** QIA136, PIMUZ 39291; **V**, QIA134, PIMUZ 39292. **I**, **J**, **P**, **AA** *Novispathodus* n. sp. Z; **I** QIA138, PIMUZ 39247; **J** QIA138, PIMUZ 39248; **P** LIL506, PIMUZ 39249; **AA** QIA136, PIMUZ 39250. **N**, **O**, **S** *Novispathodus* ex gr. *pingdingshanensis* (Zhao & Orchard); **N** LIL506, PIMUZ 39234; **O** LIL505, PIMUZ 39235; **S** QIA136, PIMUZ 39236. **R** sp. indet.; LIL511, PIMUZ 39296. **T**, **Y**, **Z** *Novispathodus* n. sp. A; **T** QIA134, PIMUZ 39256, **Y** QIA135, PIMUZ 39257; **Z** QIA134, PIMUZ 39258. **U** *Novispathodus expansus* (Zhao & Orchard)*;* QIA134, PIMUZ 39242. **W** *Novispathodus gryphus* n. sp.; QIA135, PIMUZ 39246

*Remarks.* Goudemand (in Goudemand et al., 2012b) revised the original diagnosis by noticing that the basal margin is not necessarily straight and therefore, the most strikingly difference to *Nv. waageni* is the denticulation: the denticles axes are distinctively recurved posteriorly whereas in *Nv. waageni* and *Nv. abruptus,* the denticles are straight, inclined or radiating. The two or three denticles anterior of the cusp are often clearly asymmetrical and the posterior edge of the element is much shorter than the anterior one. In most sections worldwide, *Nv. pingdingshanensis* first occurs within the positive δ^13^C_carb_ excursion of the latest Smithian and may extend to the earliest Spathian (Goudemand et al., 2019; Leu et al., 2019; Zhang et al., 2019). Some elements within our rich material resemble *Ns. Pingdingshanensis*, but appear to have a relatively small basal cavity, more posteriorly recurved denticles or more numerous denticles that what was previously described for this species. These elements (Figs. 17C, J, K, T; 18E, I–K; 19O, Q; 20H, N–O, S; here assigned to *N.* ex gr. *pingdingshanensis*) may deserve differentiation in the future. Two elements illustrated by Metcalfe et al., (2013, figs. 9.6, 9.9, p. 1144) as ‘*Neospathodus waageni* subsp. nov. A’ display a higher anterior end and more recurved denticles than the other elements they included in ‘*Neospathodus waageni* subsp. nov. A’, which we synonymized with *Ns. pingdingshanensis*: elements like these two elements do not seem to fit in *Ns. pingdingshanensis* and may deserve assignment to a new species. Some further elements resembling *Ns. pingdingshanensis* but distinctively shorter than *Ns. pingdingshanensis* are herein assigned to a new species (*Nv. gryphus* n. sp. see below).

*Occurrence*. Worldwide occurrence. China: Jinya/Waili area, Guangxi (Goudemand et al., 2012b) Jiarong, southern Guizhou (Chen et al., 2013, 2015). Anshun Fm., Qingyan section, Guizhou (Ji et al., 2011). Chaohu (Zhao et al., 2007, 2008). Daxiakou, Hubei (Zhao et al., 2013). Vietnam: Bac Thuy Fm., *Xenoceltites variocostatus* and *Tirolites* beds, (Komatsu et al., 2016; Shigeta et al., 2014). Canada: *Scythogondolella mosheri* zone, Wapiti Lake (Orchard & Zonneveld, 2009). Australia: Hovea Member of Kockatea Shale, upper part of *Neospathodus waageni* Zone, Smithian substage (Metcalfe et al., 2013).

*Novispathodus gryphus* n. sp.

Fig. 17G–I, K?, L

*Etymology*: from the Greek root ‘gryph’ referring to the hooked shape of the P_1_ element.

*Holotype:* specimen illustrated in Fig. 17H.

*Paratype:* specimen illustrated in Fig. 17L.

*Type locality:* Qiakong, Luolou formation, Guizhou Province, China.

*Type level*: Luolou Formation, within latest Smithian black shales.

*Number of specimens.* ca. 10 specimens.

*Diagnosis*. A species with a short segminate-to-segminiscaphate P_1_ element with 3 to 5 highly recurved denticles and a relatively large, sub-rounded basal cavity.

*Description.* The P_1_ element is segminate to segminiscaphate, the sub-rounded basal cavity extending to most of the length of the element with a tapering at the anterior end. The denticles are mostly fused and highly recurved posteriorly: although the denticles get larger from the anterior to the posterior, except for the posteriormost one, the height of the carina looks sub-uniform because of the way the denticles are recurved. The length-to-height ratio is about 1:1.

*Remarks.* These elements resemble strongly the homologous ones of *Nv*. *pingdingshanensis*, except that they are much shorter and bear less denticles, which are usually more recurved. Still, some may be confused with broken elements of *Nv*. *pingdingshanensis* whose anteriormost end is missing (Fig. 17G). Hence the anterior end must be inspected for traces of breakage. Although the specimens we have seen do not show obvious, surfacial traces of breakage, it is not excluded that some of these elements were broken and were subsequently repaired, thus covering such traces at the surface of the crown. Goudemand and Orchard (in Goudemand et al., 2012b) differentiated similar but longer elements as *Nv*. aff. *pingdingshanensis* (Goudemand et al., 2012b, figs. 2K, 2L) because they are lower and their denticles more recurved than in elements of *Nv*. *pingdingshanensis*: their denticles are so recurved that their upper profile appears straight; as such they are reminiscent of the present species, with whom they may be closely related. In comparison with *Nv. soleiformis* (Zhao & Orchard, 2008) *Nv. gryphus* n. sp. has a higher carina with less numerous but more posteriorly recurved denticles. Furthermore, the basal cavity of *Nv. gryphus* n. sp. does not extend as a wide deep groove anteriorly like in *Nv. soleiformis*.

*Occurrence.* Luolou Formation, within the latest Smithian black shales, Guangxi and Guizhou, South China (this study).

*Novispathodus* ex gr. *abruptus* (Orchard, 1995)

Figs. 16E; 17W, Y, Z, AE; 18H; 20A–G, K, L, M, Q, X

1981 *Neospathodus homeri* Bender; Koike, pl. 1, fig. 5.

1984 *Neospathodus* sp. A; Hatleberg & Clark, pl. 3, fig. 8, 21.

*1995 *Neospathodus abruptus* n. sp.; Orchard, p. 118, 119, figs. 3.16–3.19, 3.23–3.26.

2005 *Novispathodus abruptus* (Orchard); Orchard, p. 90, text-fig. 16.

2009 *Novispathodus abruptus* (Orchard); Orchard & Zonneveld, p. 784, fig. 15 parts 34–37.

2012a Novispathodus sp. nov. A; Goudemand & Orchard in Goudemand et al., p. 1031, figs. 2A, R?, Z?.

2012a Novispathodus sp. nov. B; Goudemand & Orchard in Goudemand et al., p. 1031, fig. 3V.

2018 *Novispathodus abruptus* (Orchard); Maekawa in Maekawa et al., p. 33, figs., 18.1–18.3, 18.20, 18.23, 18.24, 18.26, 18.27 (only).

*Material*. > 50 specimens.

*Diagnosis*. As in Orchard, 1995.

*Remarks*. Here we consider *Nv. abruptus* in a broad sense, including elements that correspond to *Nv. abruptus* sensu stricto together with forms that have been suggested to deserve assignment to separate species, such as *Nv*. sp. nov. A and *Nv*. sp. nov. B Goudemand and Orchard (in Goudemand et al., 2012b). We consider the most diagnostic feature of *Nv*. ex gr. *abruptus* is the terminal 1–3 progressively smaller denticles at the posterior end.

The P_1_ element of this species is less robust, more rectangular with more fused denticles than that of *Ic*. *crassatus*. Its morphology also recalls that of the homologous element in *Nv*. *pingdingshanensis*, but its basal cavity is relatively smaller and its denticles are not as posteriorly recurved nor usually as broad as in the latter. The P_1_ element of *Tr. symmetricus* has more posteriorly reclined denticles, it may have a small terminal denticle but not several of increasingly smaller size. Yet, the distinction between *Tr. symmetricus* and *Nv. abruptus* may be confusing. The P_1_ element of *Tr*. *homeri* has a more elongated basal cavity and a more developed, posteriorly reclined and laterally deflected process than in both *Tr. symmetricus* and *Nv. abruptus*. Goudemand and Orchard (in Goudemand et al., 2012b) implicitly suggested that elements like those of their *Nv*. sp. nov. B, where the small terminal denticles are not increasingly smaller but of equal (small) height instead, may be transitional between *Nv. abruptus* and *Tr. homeri* (compare the elements illustrated in figs. 2.9 and 3.17 of Orchard 1995, assigned to *Tr. homeri* and *Nv. Abruptus*, respectively). Note further that, based on the material from Tsoteng, it is likely that such P_1_ elements were still associated with a *Novispathodus* apparatus. This suggests that P_1_ elements like that of *Tr. homeri* may have evolved before the more substantial modifications of the rest of the apparatus implied by the difference between *Novispathodus* and *Triassospathodus*.

*Occurrence*. Worldwide occurrence in latest Smithian and early Spathian rocks. *Xenoceltites* and *Tirolites* beds within *Nv*. *pingdingshanensis* and *Nv*. *brevissimus* zones, Japan (Koike, 1981; Maekawa et al., 2018). Oman (Orchard, 1995). British Columbia (Orchard & Zonneveld, 2009), South China; Goudemand et al., 2012b; this study).

*Novispathodus* n. sp. Z Orchard, 2007

Fig. 20, I, J, P, AA

*2007 *Ns*. n. sp. Z; Orchard, p. 96, fig. 2.

2016 *Novispathodus* ex gr. *abruptus* (Orchard); Komatsu et al., p. 77, figs. 5.6a–c.

2018 *Novispathodus abruptus* (Orchard); Maekawa et al., p. 33, fig. 18.8.

2019 *Novispathodus pingdingshanensis* (Zhao & Orchard); Liu et al., p. 13, pl. 3, figs. 3, 6 (only).

2019 *Triassospathodus symmetricus*? (Orchard); Chen et al., fig. 4.15.

*Material.* ca. 15 specimens.

*Remarks*. One small terminal denticle behind the cusp is present. The denticles are mostly fused, with subtriangular tips and increasingly reclined towards the posterior end. A moderately deep, conical, subcircular shaped basal cavity is present. The overall shape of the segminate P_1_ element is reminiscent of that of the sub-coeval *Nv. pingdingshanensis* or *Nv. abruptus* except that, contrary to *Nv. pingdingshanensis*, the denticles are reclined not recurved, and contrary to *Nv. abruptus*, there is only one small denticle behind the cusp, the latter being also conspicuously broader than adjacent denticles. Our specimens from China resemble the specimens from Panthalassa California (Darwin material), suggesting that this species may be important for worldwide correlations. Orchard (2007) mentioned, that *Neospathodus kedahensis* (Koike 1973) might be an available name for his *Ns*. n. sp. Z (Orchard 2007). However, *Neospathodus kedahensis* (Koike 1973) a Middle-to-Late Triassic species, lacks a conspicuous cusp and its posterior part is composed of slightly inclined and subequal denticles.

*Occurrence*. Taho Formation, Japan (Maekawa et al., 2018). North America (Orchard 2007, Goudemand et al., in prep.). Oman: Radio Tower section, UAZ_5_ (Chen et al., 2019), South China; Nanpanjiang basin: Bac Thuy Formation, *Nv*. *pingdingshanensis* zone (Komatsu et al., 2016); Qinglong Formation, *Nv. pingdingshanensis* Zone, Jiangsu Province (Liu et al., 2019) Luolou Formation, Qiakong section, Southern Gouizhou, China (this study).

*Novispathodus brevissimus* (Orchard, 1995)

**Fig. 21:**
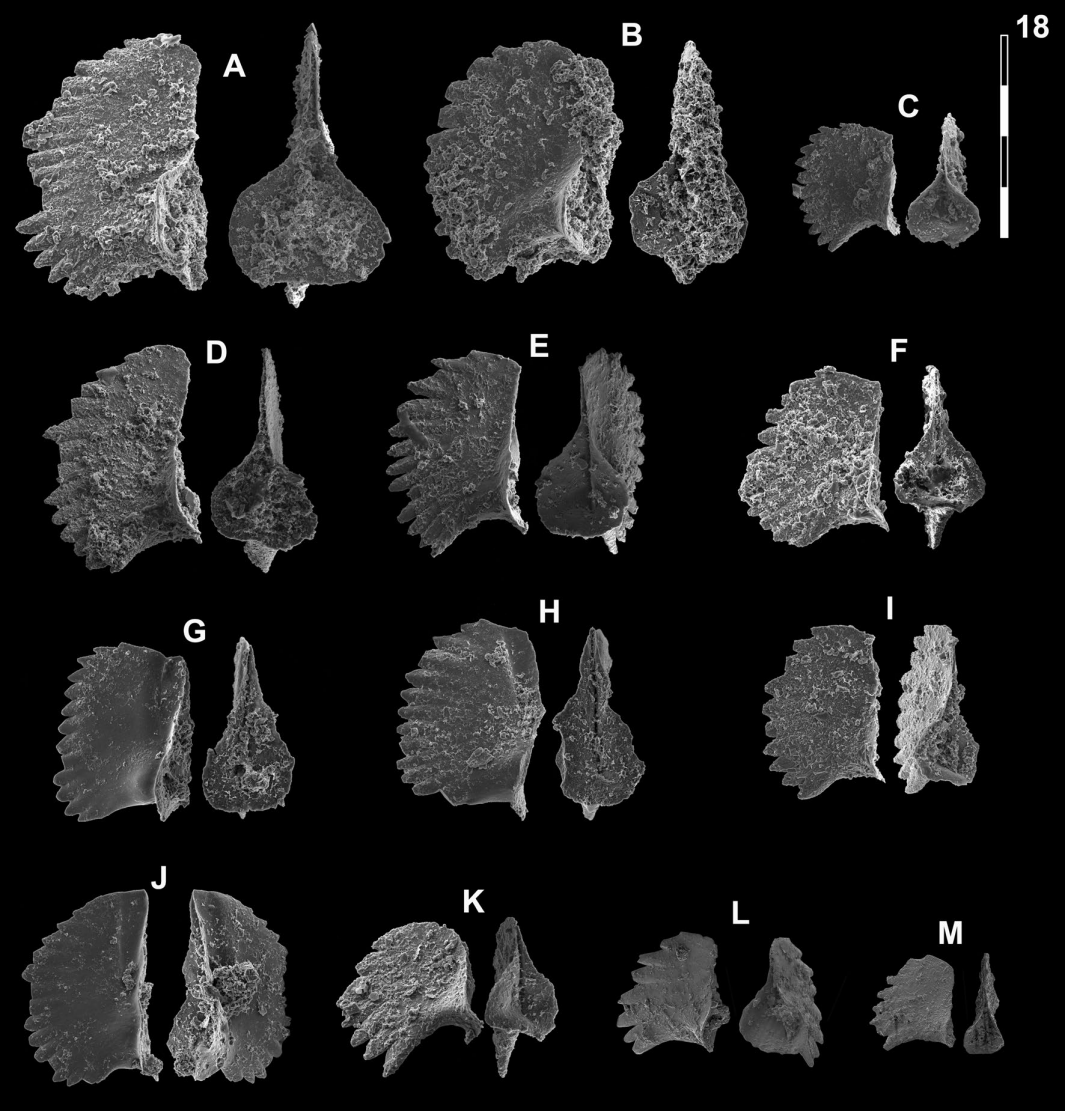
Novispathodinae from Qiakong, Laren, Shanggang and Lilong. Magnification is × 80. The scale bar is 400 μm. All elements are considered to be P_1_ elements if not specifically identified otherwise. **A**–**J**, **L**, **M**, *Novispathodus brevissimus* (Orchard); **A** LIL515A, PIMUZ 39184; **B** LIL513A, PIMUZ 39185; **C** BAN2, PIMUZ 39186; **D** SHA312, PIMUZ 39187; **E** SHA313, PIMUZ 39188; **F** LIL515A, PIMUZ 39189; **G** LIL509, PIMUZ 39190; **H** LIL509, PIMUZ 39191; **I** LIL514B, PIMUZ 39192; **J** LIL509, PIMUZ 39193; **L** QIA141, PIMUZ 39195; **M** QIA144, PIMUZ 39197. **K**, *Novispathodus clinatus* (Orchard and Sweet in Orchard); BAN5, PIMUZ 39207

**Fig. 22:**
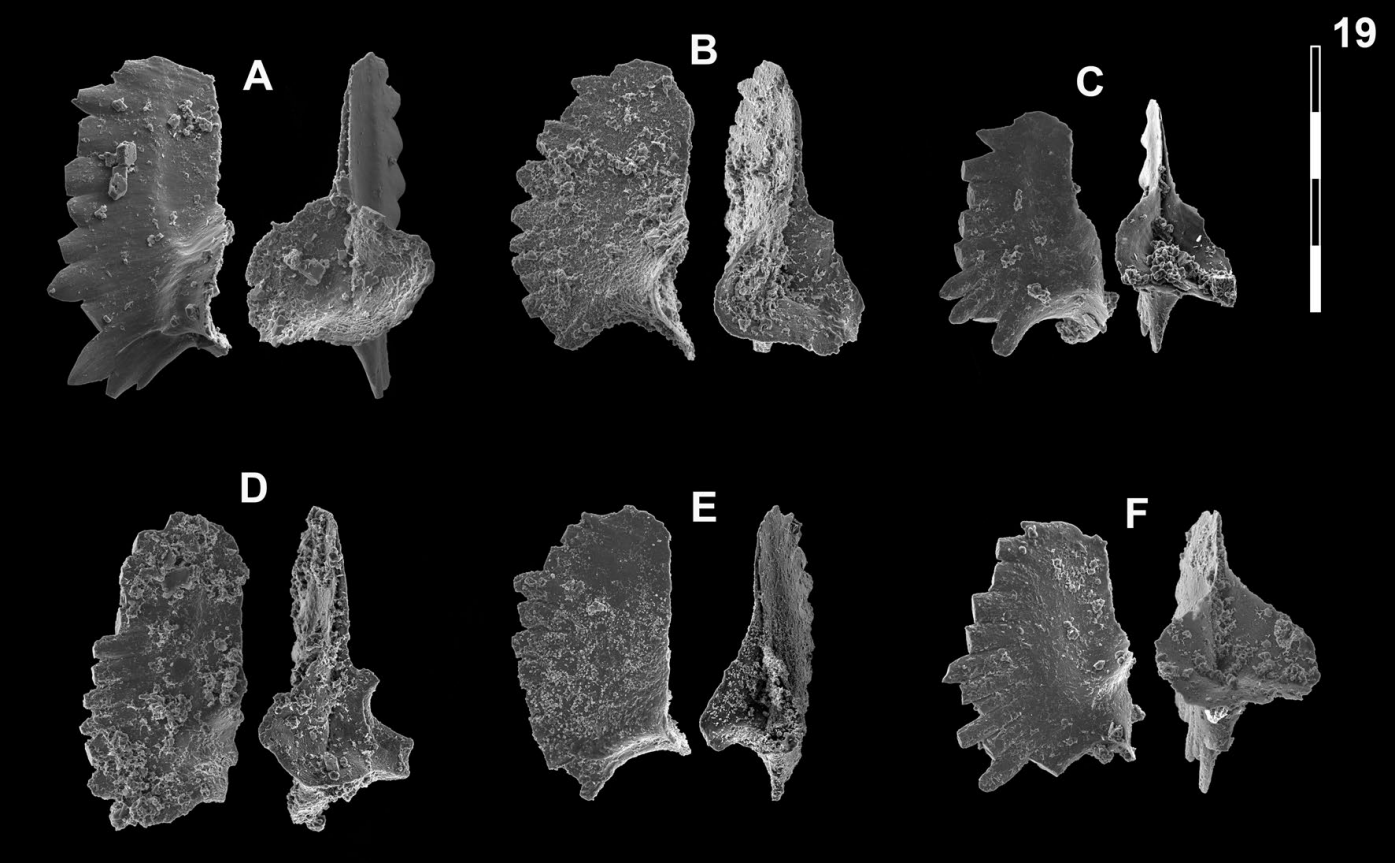
Novispathodinae from Laren and Lilong. Magnification is × 80. The scale bar is 400 μm. All elements are considered to be P_1_ elements if not specifically identified otherwise. **A**, **D** *Icriospathodus* cf. *crassatus*? (Orchard); **A** BAN1, PIMUZ 39168; **D** LIL513A, PIMUZ 39169. **B**, **E** *Novispathodus brevissimus* (Orchard); **B** LIL513B, PIMUZ 39194; **E** LIL514A, PIMUZ SQL55056. **C**, **F** *Novispathodus* cf, *brevissimus* (Orchard); **C** LIL513A, PIMUZ 39196; **F**, LIL514A, PIMUZ 39198

Figs. 19A, B, D, F; 21A–J, L, M; 22B, C, E, F?

1981 *Neospathodus triangularis* Bender; Koike, pl. 1, fig. 6.

*1995 *Neospathodus brevissimus* n. sp., Orchard, pp. 117, pl. 3, figs. 14–15, 20–22.

2011 *Neospathodus brevissimus* Orchard; Ji et al., p. 219, fig. 3, nr. 7.

2014 *Novispathodus triangularis* (Bender); Maekawa and Igo in Shigeta et al., p. 241–243, figs. 172.1–9, 172.13–27, 173.1–3, 173.6–12, 173.16–21, 173.28–44, 174.1–174.24.

2015 *Triassospathodus brevissimus* (Orchard); Yan et al., p. 240, fig. 3.4.

2016 *Novispathodus triangularis* (Bender); Komatsu et al., p. 69, fig. 5.8.

2018 *Novispathodus brevissimus* (Orchard); Maekawa in Maekawa et al., p. 34, figs. 19.4, 19.5, 19.7.

2019 *Neospathodus curtatus* Orchard; Chen et al., fig. 6 nr. 13, 14.

2019 *Neospathodus brevissimus* Orchard; Chen et al., fig. 6 nr. 15.

2019a *Triassospathodus brevissimimus* [sic.] (Orchard); Wu et al., fig. 4, nr. 36.

2019 *Novispathodus brevissimus* (Orchard); Liu et al., p. 14, pl. 4, figs. 15, 18

*Number of specimens*. > 50.

*Original diagnosis*. “Species characterized by small, short, and high segminate elements with a length:height ratio of 1:1, about 8–12 largely fused, generally upright denticles atop a deep blade, and a large basal cavity with a subcircular basal outline that occupies most of the lower side” (Orchard, 1995).

*Remarks.* Characteristic of this species is the posterior lateral margin which is slightly to strongly curved. Subcircular to subtriangular outline of the large basal cavity in the lower view. The denticles are fused and compressed with an erect to radial outline (bouquet-like). The carina is relatively high. In lateral view, the upper edge is usually straight to slightly arched. Cusp is usually undistinguishable from other denticles.

The here illustrated adult specimens are a bit larger with more denticles than the holotype (Orchard, 1995; pp. 117, Pl. 3, fig. 22), but the denticulation and the convex posterior edge of the process is very distinct in this species. Therefore, the holotype (Orchard 1995) might represent a younger, juvenile morphotype of this taxon. *Novispathodus eotriangularis* (Zhao & Orchard in Zhao et al., 2007) has a more triangle-shaped basal cavity and the anterior half of the carina is gradually declining in height, whereas *Novispathodus brevissimus* shows a more abrupt decline at the posterior end. Compared to *Novispathodus clinatus* (Orchard 1995), this species shows more numerous, short and upright denticles. *Novispathodus brevissimus* shares most similarities with *Novispathodus curtatus* (Orchard, 1995), but the latter has a relatively smaller basal cavity, is longer and bears larger and more reclined denticles. Some specimens (e.g. Fig. 19F) bear the postero-lateral pinching that is usually considered diagnostic of *Nv. triangularis* (Bender), as revised by Orchard (1995). Chen et al. (2015) illustrated very similar specimens from a higher horizon (their figs. 7.9, 7.10). Contrary to ‘true’ *Nv. triangularis*, our specimens have more rounded denticles tips and the pinching is less conspicuous, suggesting they can still be retained within *Nv. brevissimus* sensu lato.

*Occurrence.* Oman, Jabal Safra, lower Spathian (Orchard, 1995); Japan, Taho Limestone, occurs with *Nv*. *pingdingshanensis*, Tahogawa Member (Koike, 1981; Maekawa et al., 2018); Nanpanjiang basin, South China and North Vietnam, together with *Tirolites* sp. nov. and *Icriospathodus collinsoni* (Komatsu et al., 2016; Liu et al., 2019; Shigeta et al., 2014; Yan et al., 2015).

*Novispathodus expansus* (Zhao & Orchard 2008)

Fig. 20U

2008 *Neospathodus expansus n. sp.; Zhao & Orchard in Zhao et al., p. 211, pl. 1, figs, 2a, 2b, 2c.

Submitted *Novispathodus expansus* (Zhao & Orchard); Leu et al., fig. 12Q.

Number of specimens. 5.

*Remarks.* Short robust denticles, a large basal cavity and a midlateral thickening are diagnostic for *Nv. expansus*. Some specimens of *Nv. praebrevissimus* n. sp. (Fig. 18O, P, T) feature a lateral, knob-like bulging on the flanks of the basal pit, but not a flange-like thickening along the mid-part of the carina as in *Nv. expansus*. Furthermore, in the latter the carina is lower than in *Nv. praebrevissimus* n. sp.

*Occurrence. Columbites–Tirolites* Zone, Nanlinghu Formation, Lower Triassic, Chaohu, Anhui Province, China (Zhao et al., 2008) Luolou Fm. Qiakong (this study); sample, JA15C, Jebel Aweri, Batain, early Spathian age, Oman (Leu et al., submitted).

*Novispathodus clinatus* (Orchard 1995)

Fig. 21K

*1995 *Neospathodus clinatus* n. sp. Orchard & Sweet in Orchard, p. 119, figs. 3.5–3.7

*Number of specimens*. 8

*Remarks.* This segminate element is relatively short and small. The basal cavity is subtriangular in outline. The posterior and anterior margins are abrupt and show both a bowed outline. The seven denticles are uniformly reclined. Other specimens assigned to *Novispathodus clinatus* sensu lato (e.g. *Nv.* aff. *clinatus* in Chen et al., 2015, fig. 5 or *Nv.* aff. *clinatus* in Maekawa et al., 2018, figs. 19.9–19.13) usually show a more elliptical basal cavity and a blade with a higher length: height ratio with more gradually declining denticles in the anterior part. This species resembles *Nv. brevissimus* with a subtriangular shaped basal cavity but the denticles in the latter species are less uniformly reclined and more numerous.

*Occurrence.* Top of Narmia Member of the Mianwali Formation at Narmia Pakistan (Orchard 1995); Laren section, Luolou Fm., Spathian, Guangxi, South China (this study).

*Novispathodus praebrevissimus* n. sp.

Figs. 17V; 18A–D, F, J–N, O?, P?, Q, S, T; 19R

1984 *Neospathodus* sp. aff. *triangularis* Bender; Hatleberg & Clark, pl. 3, fig. 16

2014 *Icriospathodus*? *zaksi* (Buryi); Maekawa & Igo in Shigeta et al., fig. 192, nr. 10–13 (only).

2015 *Novispathodus brevissimus* (Orchard); Chen et al., p. 111, fig. 7.8.

2019 *Novispathodus pingdingshanensis* (Zhao & Orchard); Chen et al., fig. 3 nr. 5, 9 (only) fig. 5, nr. 6 (only).

2019 *Novispathodus pingdingshanensis* (Zhao & Orchard); Liu et al., p. 13, pl. 3, fig. 7 (only).

*Etymology*: According to its presumed relationship as a predecessor of *Nv*. *brevissimus*.

*Holotype:* specimen illustrated in fig. 24R

*Paratype:* specimen illustrated in fig. 24Q

*Type locality:* Lilong cliff, Luolou formation, Guangxi Province, China.

*Type level*: Luolou Formation, within latest Smithian black shales. Present in UAZ_5_ which corresponds to the peak of the positive δ^13^C_carb_ excursion and the *Xenoceltites*/*Glyptophiceras* beds.

*Number of specimens*. > 40.

*Diagnosis*. Short robust segminate P_1_ element. Large rounded-to-sub-rounded basal cavity. Posterior margin often slightly concave. Small, largely fused denticles. Groove from basal pit to anterior end.

*Description*. The segminate P_1_ element shows a very large rounded to sub-rounded basal cavity in the posterior half of the element. A very deep basal groove is present from the basal pit to the anterior end of the lower margin. The carina is relatively high with numerous small, fused denticles. The cusp is undistinguishable. The posterior lateral margin (posteriormost edge between basal cavity and last denticle) is slightly to strongly curved. Sometimes, the height of the posteriormost denticles is gradually but rapidly decreasing. In lateral view, the denticles tend to be radiating or recurved.

*Remarks*. This species is thought to be intermediate between *Nv. pingdingshanensis* and *Nv. brevissimus*. The P_1_ element of this species is very similar to that of *Nv*. *pingdingshanensis*, but the basal cavity is much larger and rounded. In comparison to *Nv*. *brevissimus,* this species is usually smaller and has a relatively bigger and more rounded basal cavity. Furthermore, the carina is lower, with less numerous denticles. The new species is distinguished from the similar *Novispathodus shirokawai* (Maekawa et al., 2018) by reclined and less pointy denticles, a larger, rounded basal cavity and a less triangular shape in lateral view. Compared to *Ic. zaksi*, the P_1_ element of this species is less robust and has a relatively higher carina. *Ns. expansus* (Zhao & Orchard) has a very similar morphology to *Nv. praebrevissimus* n. sp., but it has a distinct conspicuous thickening in the middle part of its unit. Some specimens of *Nv. praebrevissimus* n. sp. (Fig. 18L, O and P) show a bulbous thickening in the posterior part of the carina but never as pronounced as in *Ns. expansus*.

*Occurrence*. Spitsbergen, Botneheia Formation (Hatleberg & Clark, 1984). South China, Nanpanjiang basin: Jiarong, southern Guizhou (Chen et al., 2015). Luolou Formation, within latest Smithian black shales. Present in UAZ_5_ which corresponds to the peak of the positive δ^13^C_carb_ excursion and the *Xenoceltites*/*Glyptophiceras* beds, Guangxi, South China (this study). Oman (Leu et al., submitted).

*Novispathodus* ex gr. *waageni* (Sweet, 1970)

Fig. 14C, D, F, G

*1970 *Neospathodus waageni* n. sp.; Sweet, pp. 260–261, pl. 1, figs. 11, 12.

1977 *Neospathodus waageni* Sweet; Goel, p. 1094, pl. 2, figs. 1–4.

1978 *Neospathodus waageni* Sweet; Weitschat & Lehmann, pl. 14, figs. 11–12.

1979 *Neospathodus waageni* Sweet; Solien, p. 292, pl. 3, fig. 9.

1980 *Neospathodus waageni* Sweet; Chhabra & Sahni, pl. 1, figs. 9–10, 14?, 16, 20?.

1982 *Neospathodus waageni* Sweet; Koike, p. 39, pl. 6, figs. 24–27.

1983 *Neospathodus waageni* Sweet; Matsuda, p. 88–91, pl. 1, figs. 6–10.

1984 *Neospathodus waageni* Sweet; Berry et al., p. 133, pl. 1, figs. 1–4.

1984 *Neospathodus waageni* Sweet; Dagis, p. 24, pl. 7, figs. 2–5, 7–10, pl. 8. fig. 7 (only).

2004 *Neospathodus waageni* Sweet; Zhao et al., figs. 1, 3.

2007 *Neospathodus waageni* Sweet; Zhao & Orchard in Zhao et al., pp. 36–37, pl. 1, figs. 5A, B, 10A, B.

2007 *Neospathodus* ex gr. *waageni* Sweet; Orchard & Krystyn, plate, figs. 8–18.

2008 *Neospathodus waageni* Sweet; Nakrem et al., figs. 5.7, 5.8, 5.11, 5.14.

2008 *Neospathodus waageni* Sweet; Orchard, p. 406, pl. 8, figs. 8.1, 8.2, 8.8, 8.9.

2009 *Novispathodus waageni* (Sweet); Orchard & Zonneveld, p. 785, figs. 13.1–13.10, 14, 15.

2009 *Neospathodus* ex gr. *waageni* Sweet; Igo in Shigeta et al., p. 194, figs. 152.1, 152.3, 152.14–15?, 152.1618, 152.19?, 153.8–9, 156.9, 156.14–19.

**Fig. 23:**
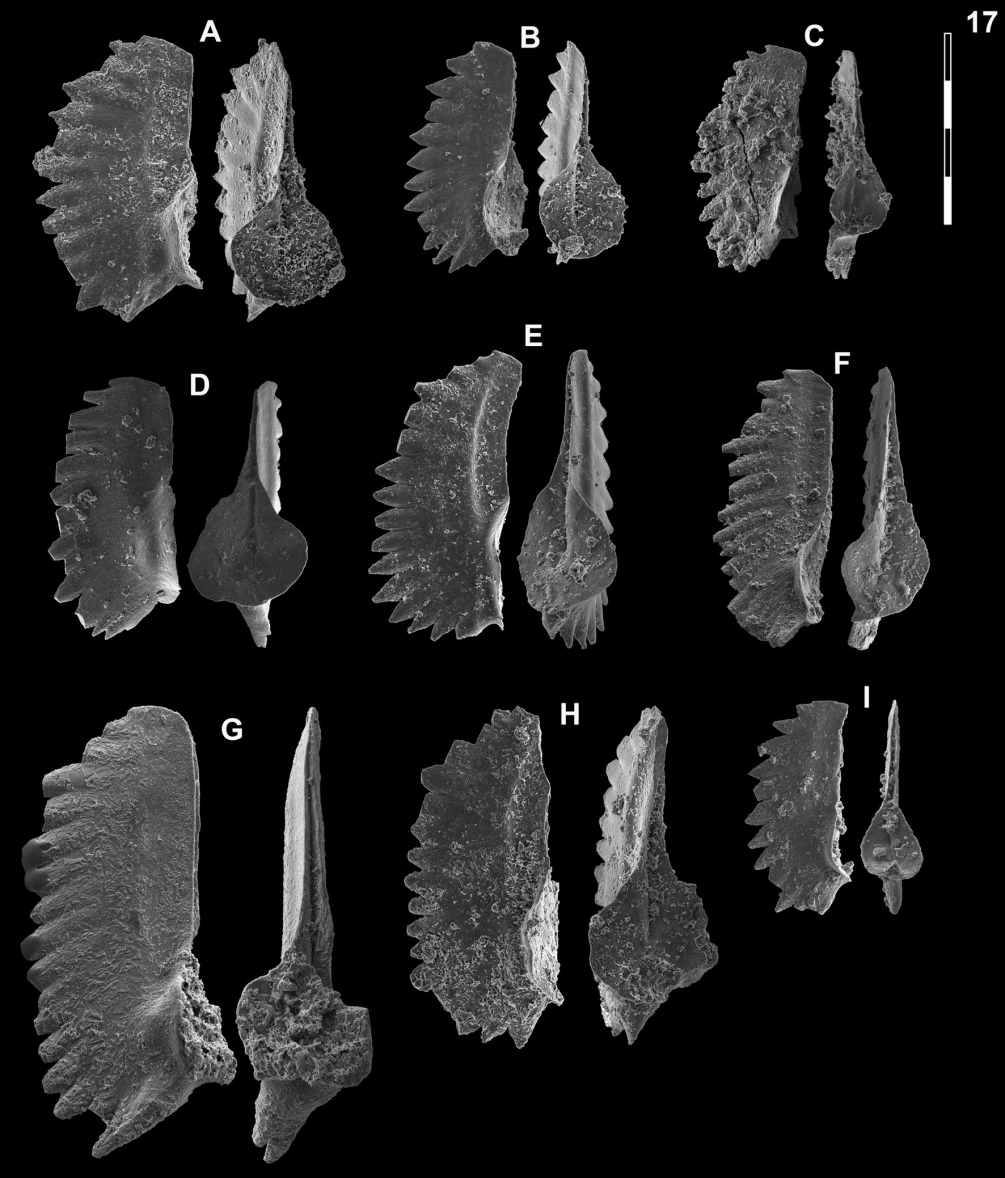
Novispathodinae from Qiakong, Laren, Shanggang and Lilong. Magnification is × 80. The scale bar is 400 μm. All elements are considered to be P_1_ elements if not specifically identified otherwise. **A**, **B**, **D**, **E**, **H**, **I**, *Triassospathodus symmetricus* (Orchard); A, LIL509, PIMUZ 39320; B, LIL509, PIMUZ 39321; D, QIA202, PIMUZ 39322; E, LIL509, PIMUZ 39323; H, LIL509, PIMUZ 39324; I, BAN2, PIMUZ 39325. **C**, **F**, **G**, *Triassospathodus homeri* (Bender); C, LAR231C, PIMUZ 39310; F, QIA155, PIMUZ 39311; G, SHA318, PIMUZ 39312

2010 *Novispathodus waageni* (Sweet); Beranek et al., figs. 6.22–23.

2012a *Novispathodus waageni* (Sweet); Goudemand & Orchard in Goudemand et al., p. 1031, figs. 3C?, D, E, H, N, S.

2013 *Novispathodus waageni* (Sweet); Zhao et al., figs. 9CC, 10A-H, 11F?, 11P-R?.

2014 *Novispathodus waageni* n. subsp. A; Goudemand, figs. 1A–1D

2014 *Novispathodus* ex gr. *waageni* (Sweet); Maekawa & Igo in Shigeta et al., p. 244, figs. 174.31–174.57, 175–178, 179.1–179.3, 179.7–179.12, 179.16–179.30, 180, 181.1–181.27.

2015 *Novispathodus waageni* (Sweet); Chen et al., figs. 6.23, 7.11, 8.3, 8.8, 8.9, 8.11, 8.14.

2016 *Novispathodus waageni* (Sweet); Liang et al., fig. 4.8.

2018 *Novispathodus* ex gr. *waageni* (Sweet); Maekawa in Maekawa et al., figs. 22.3, 22.10–11, 22.13?, 23.14–15, 23.18?.

2018 *Novispathodus waageni* (Sweet); Lyu et al., figs. 5, 6.

2019 *Novispathodus waageni* (Sweet); Lyu et al., figs. 7.8–10.

2019 *Novispathodus waageni* (Sweet); Souquet & Goudemand, figs. 3, 4a, 4g-4t.

2019 *Novispathodus* aff. *waageni* (Sweet); Souquet & Goudemand, figs. 4b–4f.

*Number of specimens*. > 50.

*Diagnosis*. See Sweet (1970).

*Remarks.* Despite the exclusion of several former morphotypes which have been formally described as separate species, this common species still encompasses a lot of variation in the P_1_ element and may deserve further differentiation in the future. Up to six morphotypes have been recognized by distinct authors, e.g. Zhao et al. (2004) or Orchard and Krystyn (2007), and several others later by Goudemand (unpublished Ph.D. thesis, 2011). It is not yet clear whether these various morphotypes may be of any utility and hence whether some of those may deserve assignment to separate species (see for instance Lyu et al., 2018 for a discussion on the value of *N. waageni eowaageni*).

*Occurrence*. *N. waageni* is a very common species in Smithian rocks worldwide. Its FAD has been proposed as a proxy for defining the base of the Olenekian (see discussions in e.g. Goudemand 2014; Lyu et al., 2018; Orchard, 2007, 2010; Orchard & Krystyn, 2007; Zhao et al., 2007; Shigeta, 2009).

*Novispathodus* n. sp. A

Figs. 19G, K, O, P, Q; 20T, Y, Z

2015 *Novispathodus* aff. *Clinatus* Orchard & Sweet; Chen et al., p. 111, fig. 7, nr. 6 (only).

*Number of specimens*. > 10.

*Description.* Segminate P_1_ element. 6–10 denticles present. The cusp is terminal and distinctly conical and often more reclined than anterior denticles. Anteriorly, the denticles are almost as high as the cusp and decline gradually towards the anterior end. The denticles are mostly fused and the cusp is conical or sometimes swollen at mid-height. The lower margin is straight to slightly upturned in the posterior half. Basal cavity rounded at posterior margin and tapers towards the anterior end. Basal groove runs from the basal pit to the anterior end.

*Remarks*. Similar to *Nv*. ex gr. *pingdingshanensis* in size and in its overall morphological outline, but differs by having a conical and terminal cusp. The denticles are straight, upright to slightly reclined, not recurved as in *Nv. pingdingshanensis.* In *Nv*. *abruptus*, the posteriormost denticles show a gradual decline that is not observed here. One of the elements illustrated by Chen et al. (2015) as *Nv*. aff. *clinatus* seems to fall within the variation of this species although it has more inclined posterior denticles.

Occurrence. In the late Smithian UAZ_7_ in Qiakong, Lilong, Guangxi, South China (this study).

Genus TRIASSOSPATHODUS Kozur et al., 1998

*Type species and holotype. Spathognathodus homeri* Bender, 1970, pp. 528–529, pl. 5, fig. 16a–c.

*Type stratum and locality.* Marmarotrapeza Formation, Marathovuno, Chios, Greece.

F23;F24

*Triassospathodus symmetricus* (Orchard, 1995)

**Fig. 24:**
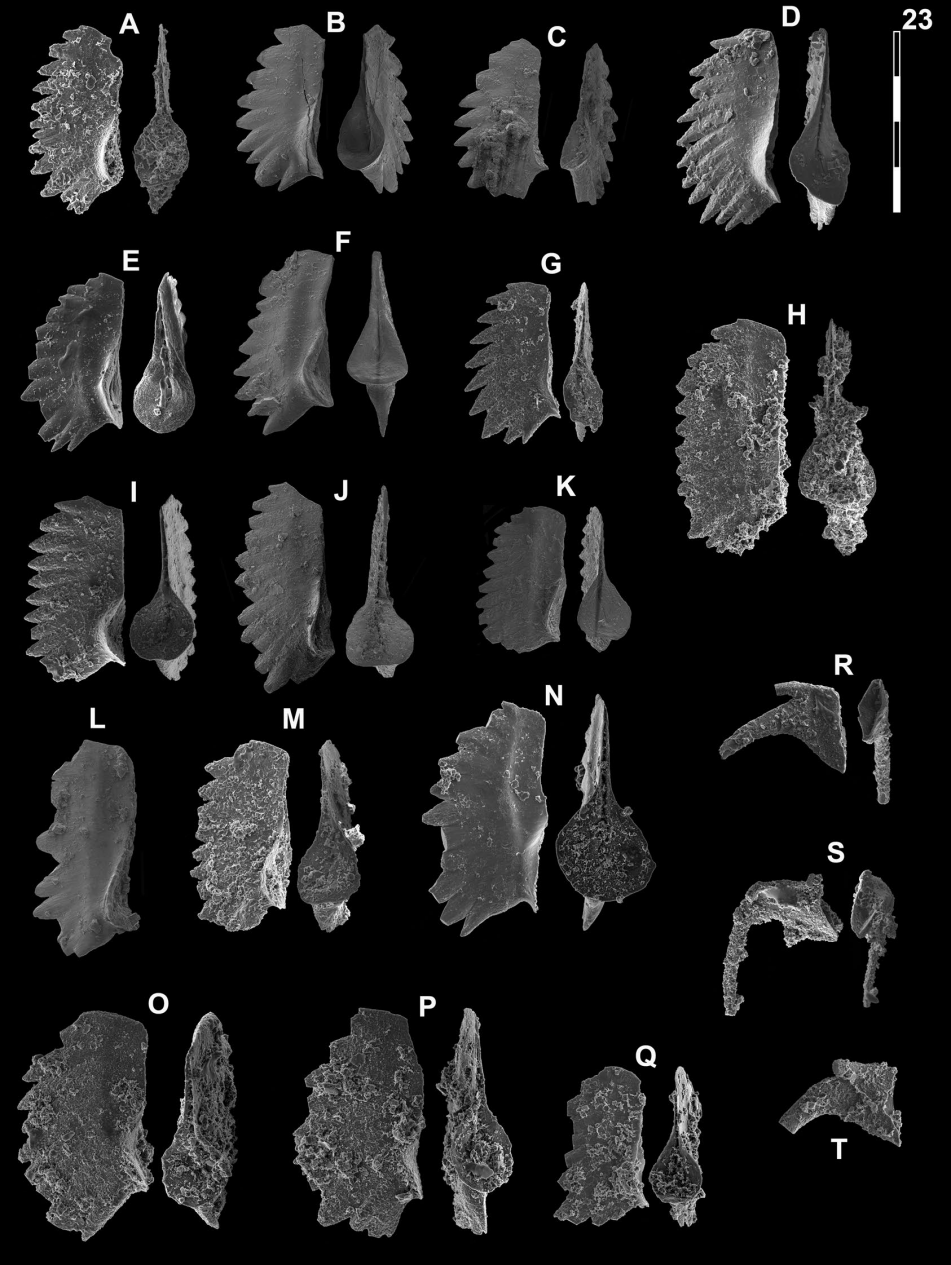
Novispathodinae and uncertain from Qiakong, Laren, Shanggang, and Lilong*.* Magnification is × 80. The scale bar is 400 μm. All elements are considered to be P_1_ elements if not specifically identified otherwise. **A**, **D**, **G**, **H** *Triassospathodus homeri* (Bender); **A** LIL515C, PIMUZ 39313; **D** QIA155, PIMUZ 39314; **G** LIL513B, PIMUZ 39315; **H** LIL515B, PIMUZ 39316. **B**, **E**, **F** *Triassospathodus* aff. *symmetricus* (Orchard); **B** QIA138, PIMUZ 39306; **E** LIL505, PIMUZ 39307; **F** QIA136, PIMUZ 39308. **C**, **I**–**Q** *Triassospathodus symmetricus* (Orchard); **C** QIA140, PIMUZ 39326; **I** SHA313, PIMUZ 39327; **J** QIA143, PIMUZ 39328; **K** QIA144, PIMUZ 39329; **L** QIA141, PIMUZ 39330; **M** LIL515D, PIMUZ 39331; **N** LIL509, PIMUZ 39332; **O** LIL513B, PIMUZ 39333; **P** LIL513B, PIMUZ 39334; **Q** LIL513A, PIMUZ 39335. **R**–**T** *Aduncodina unicosta* (Ding); **R** BAN5, PIMUZ 39100; **S** SHA320, PIMUZ 39101; **T** SHA318, PIMUZ 39102

Figs. 19H, I–M; 23A, B, D, E, H, I; 24C, I–Q

1970 *Neospathodus homeri* Bender; p.245, pl. 1, figs. 2, 3, 9, 10.

1970 *Neospathodus triangularis* Bender; Sweet, pp. 253–254, pl. 1, figs. 7, 8.

1973 *Neospathodus homeri* Bender; Mosher, p. 171, pl. 20, fig. 14.

1977 *Neospathodus homeri* Bender; Goel, p. 1097, pl. 2, figs. 10, 11.

1986 *Neospathodus homeri* Bender; Durkoop et al., pl. 20, figs. 9–10.

*1995 *Neospathodus symmetricus* n. sp.; Orchard, p. 120, 121, figs. 2.6, 2.10–2.13, 2.18.

2004 *Neospathodus symmetricus* Orchard; Koike, p. 137, figs. 35–38.

2007b *Triassospathodus* ex. gr. *homeri* (Bender); Orchard et al., p. 345, fig. 5.5, 5.6.

2009 *Triassospathodus* ex. gr. *homeri* (Bender); Orchard & Zonneveld, p. 788, fig. 15, parts 38–40.

2011 *Neospathodus symmetricus* Orchard; Ji et al., p. 219, figs. 3.5a, b, c.

2014 *Triassospathodus symmetricus* (Orchard); Maekawa & Igo in Shigeta et al., p. 254, figs. 182–185, 186.1–186.3

2015 *Triassospathodus symmetricus* (Orchard); Chen et al., figs. 7.16–7.17, 8.19, 9.15.

2015 *Novispathodus abruptus* (Orchard); Chen et al., figs. 8.2, 9.13.

2018 *Novispathodus abruptus* (Orchard); Maekawa in Maekawa et al., p. 33, figs. 17.22, 17.24, 17.25, 18.4–18.12, 18.14–18.22, 18.25, 18.28 (only).

2019 *Triassospathodus symmetricus* (Orchard); Chen et al., fig. 4, nr. 8, 1–13, fig. 5, nr. 4 (only)

Number of specimens. >50

*Description*. Segminate P_1_ element with length/height ratio of 2.0–2.5:1 and usually 10–13 subequal, variably fused denticles that become increasingly reclined posteriorly. Straight or arcuate upper margin, subtriangular denticles edges. The cusp is indistinct, it has as high or slightly higher than the other denticles. The basal margin is straight anteriorly and may be downcurved posteriorly. The margin of variably shaped basal cavity is expanded laterally and downturned posteriorly. A basal groove extends from the basal pit to the anterior end. Some specimens develop a midlateral rib.

*Remarks*. The elements are similar to *Tr*. *homeri* but usually shorter and they lack a ‘true’ denticulate posterior process: in *Tr*. *symmetricus*, the basal cavity is more or less rounded posteriorly, whereas in *Tr*. *homeri* it tapers posteriorly below the inturned posterior edge of the carina (Orchard, 1995). Some elements (Fig. 23B, E, H) may deserve differentiation in the future as they bear strongly reclined and gradually smaller, posteriormost denticles, as opposed to typical elements of this species.

*Occurrence*. Almost worldwide distribution. Nanpanjiang basin: North-eastern Vietnam, Bac Thuy Formation, *Tirolites* cf. *cassianus* beds (Shigeta et al., 2014). South China, Luolou Formation (several sections in this paper), Qingyan section, (*Neospathodus homeri* Zone, Ji et al., 2011); Northern Indian margin: Salt Range, Pakistan (Zone 9, Sweet, 1970). Spiti India (Goel, 1977), Oman, Jabal Safra (Orchard, 1995). Japan: Taho limestone (Koike, 2004). British Columbia: Canada (*Keyserlingites subrobustus* Zone, Mosher, 1973).

*Triassospathodus* aff. *symmetricus* (Orchard, 1995)

Figs. 18G; 24B, E, F

2004 *Neospathodus* sp. aff. *N. symmetricus* Orchard; Koike, p. 133, figs. 2.4–2.5.

2015 *Triassospathodus symmetricus* (Orchard); Chen et al., fig. 8.1.

*Number of specimens*. > 20.

*Remarks*. The biostratigraphical range of these specimens is usually slightly older than that of *Tr. symmetricus*. The P_1_ element of *Tr*. aff. *symmetricus* resembles that of *Tr. symmetricus*, but is shorter with fewer denticles and a more circular and larger outline of the basal cavity. The denticles are less inclined posteriorly compared to *Tr. symmetricus* and often have one additional small posteriormost denticle. Its denticles are not posteriorly arched as in *Nv. pingdingshanensis*. It differs from the very similar *Nv*. *abruptus* by having a downturned posterior part and posteriorly reclined denticles. This species might represent a transitional form between *Novispathodus abruptus* and *Triassospathodus symmetricus* but a multi-element apparatus reconstruction is currently lacking.

*Occurrence*. Taho Formation, Southwest Japan, Spathian age (Koike, 2004); South China, Luolou Formation, at the top of the black shales and the base of the nodular limestone in the earliest Spathian (this study).

*Triassospathodus homeri* (Bender, 1970)

Figs. 19C; 23C, F, G; 24A, D, G, H

*1970 *Spathognathodus homeri* n. sp.; Bender, p. 528, pl. 5, figs. 16a–c.

1970 *Neospathodus homeri* (Bender); Sweet, p.245, pl. 1, figs. 2, 3, 9, 10.

1980 *Neospathodus homeri* (Bender); Chhabra & Sahni, pl. 1, figs. 28 (only).

1983 *Neospathodus homeri* (Bender); Matsuda, p. 94–95, pl. 4, figs. 3, 4, 5.

1986 *Neospathodus homeri* (Bender); Durkoop et al., pl. 20, figs. 8a–d.

1995 *Neospathodus homeri* (Bender); Orchard, p. 115, figs. 2.1–2.3, 2.7–2.9, 2.14–2.17, 2.20, 2.21.

2005 *Triassospathodus homeri* (Bender); Orchard, p. 93, figs. 19.

2007 *Triassospathodus* ex. gr. *homeri* (Bender); Lucas & Orchard, p. 123, figs. 7.8, 7.9.

2007b *Triassospathodus* ex. gr. *homeri* (Bender); Orchard et al., p. 353, figs. 6, nr. 10–12.

2011 *Neospathodus homeri* (Bender); Ji et al., p. 220, figs. 3.9a, b, c.

2014 *Triassospathodus homeri* (Bender); Maekawa & Igo in Shigeta et al., p. 253, figs. 181.43–181.48.

2015 *Triassospathodus homeri* (Bender); Chen et al., figs. 7.13, 8.18, 9.14, 9.19.

2015 *Novispathodus abruptus* (Orchard); Chen et al., figs. 7.16–7.17, 9.16.

2019 *Triassospathodus homeri* (Bender); Chen et al., fig. 7, nr. 1.

2019 *Triassospathodus symmetrucis* [sic.] (Orchard); Liu et al., p. 12, pl. 2, fig. 11.

2021 *Novispathodus abruptus* (Orchard); Chen et al., fig. 4.3.

2021 *Triassospathodus homeri* (Bender); Chen et al., fig. 4.15, 4.16 (only).

Number of specimens: >30

*Diagnosis*. “Species with segminate elements that have a length:height ratio of 2.5–3:1, and commonly 15–18 subequal, moderately fused denticles that are increasingly reclined toward the posterior end where 3–5 small, low denticles occur on a short, variably inturned process. The posterior edge of the blade is moderately reclined. In basal outline, the basal cavity is elliptical, tapers in both anterior and posterior directions, and is strongly asymmetrical in specimens with an inturned posterior process.” (Orchard, 1995).

*Remarks*. This species may be slightly younger than the closely related *Tr*. *symmetricus*.

*Occurrence*. Worldwide distribution. Nanpanjiang basin: North-eastern Vietnam, Bac Thuy Formation, *Tirolites* beds (Shigeta et al., 2014). South China, (Orchard et al., 2007b, this study). Northern Indian margin: Kashmir (Matsuda, 1983). Jabal Safra, Oman (Orchard, 1995). Salt Range, Pakistan (Sweet, 1970). Western USA: Lower Spathian interval of Thaynes Group (*Tirolites* beds, Lucas & Orchard, 2007).

Genus ICRIOSPATHODUS Krahl et al., 1983

*Type species and holotype. Neospathodus collinsoni* Solien, 1979.

*Type stratum and locality.* Unit D, Thaynes Formation, near Salt Lake City, Utah, USA.

*Remarks.* Originally, the basis for establishing this genus was the characteristic ridge-like denticulation of the P_1_ element of *Ic*. *collinsoni*. Orchard (2005) reconstructed the multi-element apparatus of *Ic. collinsoni*, showing a smaller degree of denticulation of the P_2_, M and S elements in comparison to *Novispathodus*. Because their S elements (especially the S_1_) are significantly different from those of the subfamily Novispathodinae, he regarded their subfamily assignment as uncertain. Pending future multi-element reconstructions of the herein included taxa, we tentatively retain *Icriospathodus* within the Novispathodinae. The affinity of *Ic*. *collinsoni*, *Ic*. *crassatus* and *Ic*. *zaksi* was recognized in several studies (Koike, 1992; Orchard, 2007; Maekawa & Igo in Shigeta et al., 2014; Maekawa in Maekawa et al., 2018), and we follow these authors.

*Icriospathodus collinsoni* (Solien, 1979)

Fig. 25M, O, P

**Fig. 25:**
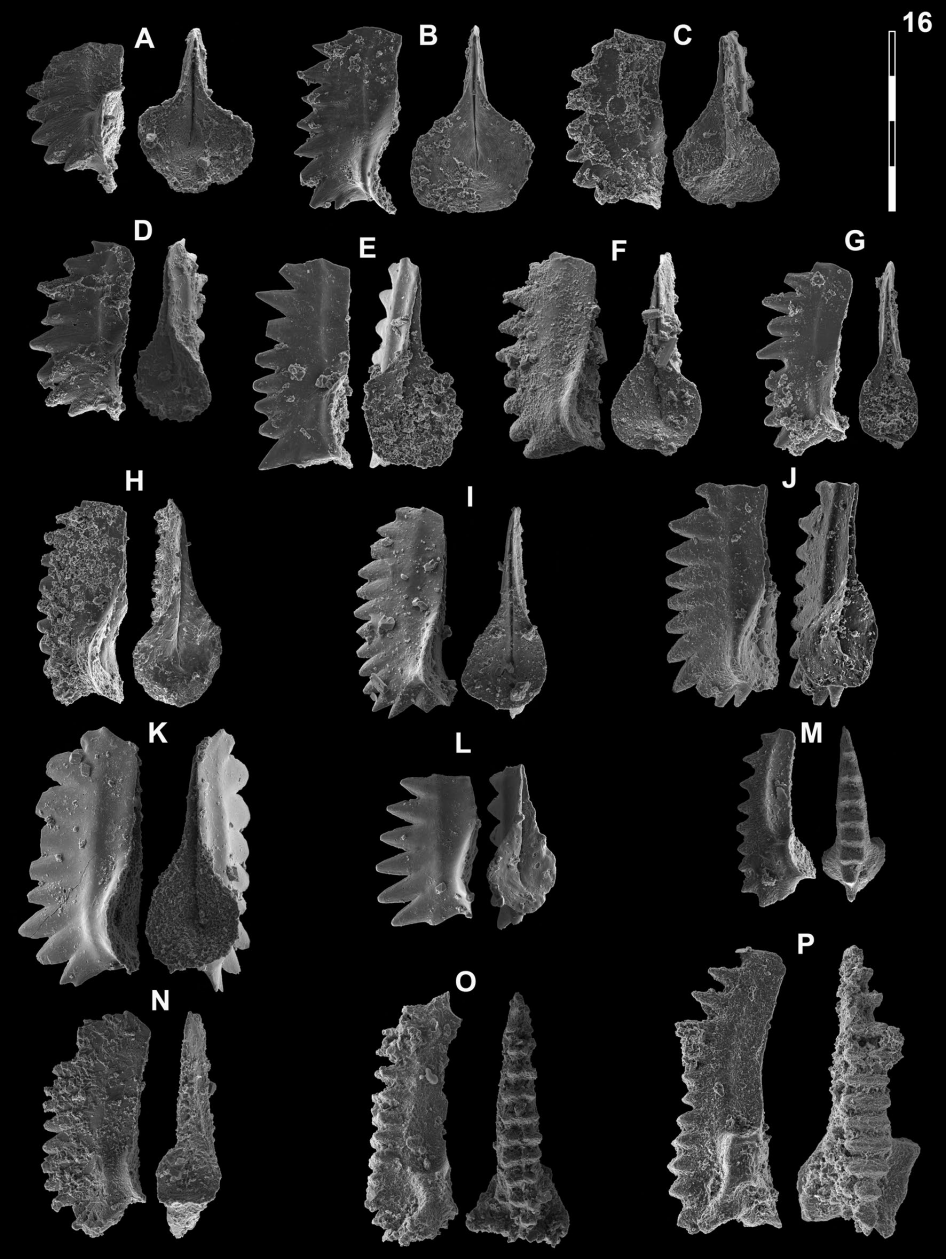
Novispathodinae from Laren, Shanggang and Lilong. Magnification is × 80. The scale bar is 400 μm. All elements are considered to be P_1_ elements if not specifically identified otherwise. **A**–**F**, *Icriospathodus zaksi* (Buryi); **A** LAR205, PIMUZ 39170; **B** LIL508, PIMUZ 39171; **C** LIL508, PIMUZ 39172; **D** LAR204, PIMUZ 39173; **E** LIL510, PIMUZ 39174; **F** LAR203, PIMUZ 39175. **G**–**L** *Icriospathodus* aff. *crassatus* (Orchard); **G** LIL510, PIMUZ 39159; **H** LIL508, PIMUZ 39160; **I** LAR203, PIMUZ 39161; **J** LIL509, PIMUZ 39162; **K** LAR202, PIMUZ 39163; **L** LAR210, PIMUZ 39164. **M**, **O**, **P** *Icriospathodus collinsoni* (Solien); **M** SHA313, PIMUZ 39165; **O** SHA320, PIMUZ 39166; **P** LIL515A, PIMUZ 39167. **N** *Triassospathodus symmetricus* (Orchard); SHA320, PIMUZ 39319

1964 *Icriodus*; Clark et al., p. 376, pl. 60, fig. 1.

1970 *Neospathodus* n. sp. G; Haselmüller, p. 45–47, pl. 2, fig. 12a–b.

1971 *Neospathodus* n. sp. G; Sweet et al., p. 453, pl. 1, figs. 12, 13

*1979 *Neospathodus collinsoni* n. sp.; Solien, p.302, pl. 3, figs. 10, 12–20

1981 *Neospathodus*? *collinsoni* (Solien); Koike, pl. 1, figs. 42–44.

1987 *Neospathodus collinsoni* (Solien); Zakharov & Rybalka, p. 43–44, pl. 5, figs. 4, 5.

1990 *Neospathodus collinsoni* (Solien); Metcalfe, p. 136, pl. 1, figs. 6, 7, 17.

1992 *Spathoicriodus collinsoni* (Solien); Koike, p. 357–361, figs. 11, 12.13–12.42, 12.44, 12.47–12.50, 13.8–13.37.

1995 *Icriospathodus collinsoni* (Solien); Orchard, p. 113, fig. 2.22–2.24.

2005 *Icriospathodus collinsoni* (Solien); Orchard, p.96, fig. 22A

2007 *Icriospathodus collinsoni* (Solien); Orchard p.96, fig. 2

2007 *Icriospathodus collinsoni* (Solien); Lucas & Orchard, p. 123, figs. 7.4–7.7, 7.13–7.15.

2011 *Icriospathodus collinsoni* (Solien); Ji et al., p. 221, figs. 5a–5c.

2014 *Icriospathodus collinsoni* (Solien); Maekawa & Igo in Shigeta et al., p. 260, figs. 186.10–186.22, 187–191, 192.1–192.6.

2015 *Icriospathodus collinsoni* (Solien); Chen et al., p.113, figs. 9.1–9.4.

2015 *Icriospathodus collinsoni* (Solien); Yan et al., p. 240, figs. 3.5, 3.6.

2016 *Icriospathodus collinsoni* (Solien); Komatsu et al., p. 69, fig. 5.9.

2018 *Icriospathodus collinsoni* (Solien); Maekawa in Maekawa et al., p. 49–51, figs. 28.5–28.10, 29.1–29.7.

2019 *Icriospathodus collinsoni* (Solien); Chen et al., fig. 6, nr. 10–12.

2019 *Icriospathodus collinsoni* (Solien); Liu et al., p. 14, pl. 4, figs. 6–8.

2021 *Icriospathodus collinsoni* (Solien); Chen et al., figs. 4.9–11.

Number of specimens: >40

*Description*. A robust segminate P_1_ element. The upper edge bears biserial or/and ridge-like denticles. The width of the denticles is usually longest in the middle part to the posterior third. The basal cavity shows a lot of variation, but is mostly asymmetrical.

*Remarks*. Koike (1992) showed a wide range of intraspecific variation within this species (see below). The two rows of nodes on the upper margin and the pairs of nodes connected by a ridge are diagnostic features that are easy to determine and miss-identification of even broken elements is unlikely. P_1_ elements of *Eurygnathodus* are superficially similar but they are scaphate, not segminate.

*Occurrence*. *I. collinsoni* indicates the early Spathian worldwide and generally co-occurs with the *Tirolites* and *Columbites* beds. USA: Idaho, (Zone 11, Sweet et al., 1971), Utah (Thaynes formation, Solien, 1979), Nevada (Lucas & Orchard, 2007), South China (Chen et al., 2015; Ji et al., 2011), Vietnam (Bac Thuy formation, Komatsu et al., 2016; Shigeta et al., 2014), Oman (Orchard, 1995), Primorye (Zakharov & Rybalka, 1987), Japan (Maekawa et al., 2018), Malay Peninsula (Metcalfe, 1990).

*Icriospathodus* aff. *crassatus* (Orchard, 1995)

Figs. 22A?, D?; 25G–L, N

2019 *Icriospathodus crassatus* (Orchard); Liu et al., pl. 4, figs. 10, 14.

Number of specimens: >30

*Diagnosis.* In lateral view, the segminate P_1_ element is subrectangular. Moderately fused carina. Denticles increasingly lower at both anterior and posterior ends. Posteriormost denticles often bent inwards. Basal cavity generally symmetrical and subquadrate to subtriangular in shape. Lateral margins and basal cup often relatively thick and sometimes accessory nodes on the latter.

*Description.* In oral view, the rather symmetrical large basal cavity looks bulbous. Laterally, the thick node-like, irregular denticles are lower and more bent inwards towards the posterior end. The basal cavity is symmetrical. The subterminal cusp is situated near the posterior end, up to two more reclined denticles being sometimes present behind it. The lower margin is downturned posteriorly.

*Remarks.* Orchard (1995) created *Ic. crassatus* for *Ic. collinsoni*-like elements that lack the ridge-like or paired platform nodes. Our specimens share the attributes of *Ic. crassatus* but their basal cavities are subsymmetrical, which is uncommon for *Ic. crassatus*. The elements of *N. symmetricus* have a similar outline in lateral view but have relatively higher and more uniform denticles with a pointy tip, and a relatively larger basal cavity. In our material, *Ic. zaksi* and *Ic.* aff. *crassatus* are morphologically similar, but *Ic*. aff. *crassatus* is longer, bears more denticles, has a relatively smaller basal cavity and is usually found in younger strata. *Ic. zaksi* may be a forerunner of *Ic*. aff. *crassatus*. In the material studied from South China, no “true” *Ic. crassatus* was found. In addition, all illustrated specimens from South China determined as *Ic. crassatus* from the literature can neither be determined as *Ic. crassatus* sensu stricto in the authors view. The specimen illustrated in Lehrmann et al. (2015) (fig. 5.16, 5.17), resembles more *Tr. homeri* (see Additional files)*.* The studies from West and North Pingdingshan do either not provide any illustrations (Liang et al., 2011; Zhao et al., 2007, 2008) or the specimens cannot be determined as *Ic. crassatus* with certitude because the specimens are broken and resembles more *Ic*. cf. *zaksi* (Pl. 6 fig. 2) or *Ic*. cf. aff. *crassatus* (Pl. 12 fig. 11) in Zhao (2005). In Ji et al. (2011) the illustrated specimen from Qingyan consists of a large subsymmetrical basal cavity and resembles more *Ic*. aff. *crassatus*. The specimen determined as Ic. *crassatus* in Liu et al. (2019) is in the synonymy list of *Ic*. aff. *crassatus* (see above). Furthermore, the specimen illustrated in Yan et al. (fig. 6GG, 2013) resembles more *Tr. homeri* with an inturned posterior end. Therefore, it has to questioned if *Ic*. aff. *crassatus* is provincialistic of South China and is worth of defining a new species formally in the future.

*Occurrence.* South China, Nanpanjiang basin, Luolou Fm., Laren, Lilong, Shanggang top of black shales to nodular limestone, SSB and early Spathian, (this study).

*Icriospathodus zaksi* (Buryi, 1979)

Fig. 25A–F

*1979 *Neospathodus zaksi* Buryi, p. 60 pl. 18 figs. 3a, b.

2007 *Icriospathodus*? *zaksi* (Buryi); Orchard, fig. 2.

2013 *Neospathodus novaelhollandiae* McTavish; Yan et al., p. 516, fig. 6BB–DD.

2014 *Icriospathodus*? *zaksi* (Buryi); Maekawa & Igo in Shigeta et al., fig. 192, nr. 14–29.

2015 *Novispathodus pingdingshanensis* (Zhao & Orchard); Chen et al., p.111, figs. 7.1–7.2.

2016 *Neospathodus planus* sp. nov. Chen & Kolar-Jurkovšek; Chen et al., p.92, fig. 7.9a–c, 8.10.

2016 *Neospathodus robustus* Koike; Chen & Kolar-Jurkovšek in Chen et al., p.92, figs. 8.7–8.9.

2016 *Icriospathodus*? *zaksi* (Buryi); Komatsu et al., p. 69, fig. 5, nr. 2–3.

2018 *Icriospathodus zaksi* (Buryi); Henderson et al., p.18 pl. 1, figs. 18–19.

2018 *Icriospathodus zaksi* (Buryi); Maekawa in Maekawa et al., p. 51, figs. 29.20–29.26.

2018 *Icriospathodus*? sp. 1; Maekawa in Maekawa et al., p. 54, figs. 30.1–30.2.

2019 *Triassospathodus symmetricus* (Orchard); Chen et al., fig. 5, nr. 8 (only).

2019 *Neospathodus ex. gr. planus Chen & Kolar-Jurkovšek*; Chen et al., fig. 6, nr. 2.

2019 *Icriospathodus zaksi* (Buryi); Chen et al., fig. 6, nr. 4, fig. 7, nr. 9, 10.

2019 *Novispathodus pingdingshanensis* (Zhao & Orchard); Liu et al., p. 13, pl. 3, fig. 4 (only).

Number of specimens: >30

*Revised diagnosis.* Low, robust segminate P_1_ elements usually with 8–9 wide denticles. 2–3 small denticles may be located behind the variably conspicuous cusp. Denticles are node-like in form. The lower margin is straight. Deep, wide basal cavity. One or two variably shaped, lateral processes are occasionally present.

*Description.* Robust segminate P_1_ element with 7–12 short, robust, erect to uniformly recurved, mostly fused, triangular-shaped denticles. Rectangular to subrectangular outline of the element. In most elements, the cusp is distinct. Unit lowest at the posteriormost end. Big sub-rounded, bulbous, slightly concave basal cavity. The basal cavity occupies the entire posterior half of the element. The posterior margin coincides with the edge of the cavity.

*Remarks.* The holotype (Buryi, 1979) has postero-lateral processes, which, in the original diagnosis were considered “complex in form” and characteristic of this species. Because the holotype is broken and the original diagnosis was based only on that specimen, we revise here the diagnosis on the basis of more than 30 specimens that show broader morphological variation in the platform: as illustrated by others (e.g. Chen et al., 2019), a lateral process may or may not be present. Koike (1992) reported a similar morphological variation within *Ic. crassatus* and *Ic. collinsoni*, suggesting it is a generic property of *Icriospathodus*. The P_1_ element of *Ic. zaksi* resembles that of *Ic. crassatus* but it is smaller, has a shorter carina and a much larger basal cavity relatively to its length.

*Occurrence.* Worldwide occurrence. India: Unit H3, Khunamuh formation, Guryul Ravine; North-eastern Vietnam: *Novispathodus pingdingshanensis* Zone, *Xenoceltites variocostatus* beds, Bac Thuy Formation (Maekawa & Igo in Shigeta et al., 2014); Russia: upper part of *Anasibirites nevolini* Zone to lower part of *Tirolites cassianus* Zone, South Primorye (Buryi, 1979); South China: Bed 51, Luolou Formation, Bianyang Section, Guizhou province (Yan et al., 2013); Canada: British Columbia, Montney Formation; late Smithian–early Spathian (Henderson et al., 2018). Oman; Wadi Bani Khalid section, UAZ_4_, *Nv. pingdingshanensis* range Zone (Chen et al., 2019); Japan: Taho Formation, *Novispathodus brevissimus* Zone (Maekawa et al., 2018).

Subfamily MULLERINAE Orchard 2005

Genus DISCRETELLA Orchard 2005.

*Type species*. *Ctenognathodus discreta* Müller 1956, p. 821–822, pl. 95, fig. 28.

*Type stratum and locality*. Smithian ammonoid bed, Crittenden Springs, Elko County, Nevada.

*Discretella discreta* (Müller, 1956)

Figs. 15C, G, L; 16D, S.

*1956 Ctenognathodus discreta n. sp.; Müller, pp. 821–822, pl. 95, fig. 28.

1989 *Neospathodus discreta* (Müller); Thang, p. 402, pl. 30, fig. 7.

2005 *Discretella* sp. A; Orchard, p. 83, fig. 8.

2008 *Discretella discreta* (Müller); Orchard 2008, p. 402, figs. 8.18, 8.19.

2010 *Discretella discreta* (Müller); Beranek et al., p. 65, figs. 6.18, 6.19.

2014 *Discretella discreta* (Müller); Maekawa & Igo in Shigeta et al., pp. 196–202, figs. 141.13–141.33, 142–145, 146.1–146.30.

2018 *Discretella discreta* (Müller); Maekawa in Maekawa et al., p. 23, fig. 14.4.

Number of specimens: >20

*Diagnosis.* See Müller, 1956.

*Description.* The carminate-to-segminate P_1_ element has an upturned and inverted basal margin in the posterior one-third to one-half of the element. The cusp is usually larger than the other discrete and upright denticles but not conspicuously.

*Remarks.* Maekawa & Igo (in Shigeta et al., 2014) described two different morphotypes, A and B, for *Discretella discreta*. Their morphotype A would correspond to the holotype (Müller, 1956) and their morphotype B has an upturned posterior margin and a relatively broader and triangular cusp. Although Maekawa & Igo (in Shigeta et al., 2014) have recently illustrated numerous specimens, the intraspecific variation of this species remains unclear due to its general scarcity. Based on their illustrations and unpublished material, there seems to be scope for further differentiation. Both morphotypes A and B have a posterior process and hence a tapered posterior margin of the basal cavity. However, some of our specimens do not develop a posterior process and have a rounded posterior margin at the basal cavity. Those are here assigned to a different species, which for now is kept in open nomenclature (*Discretella* aff. *discreta*).

*Occurrence*. Wide global occurrence in the Smithian. *Meekoceras* beds, Nevada (Müller, 1956). *Euflemingites romunderi* Zone, Canadian Arctic (Orchard, 2008). Jabal Safra, Oman (Orchard, 2005). North Vietnam (Thang, 1989). *Nv*. ex. gr. *waageni* Zone, Taho Formation, Japan (Maekawa et al., 2018).

*Discretella* aff. *discreta* (Müller, 1956)

Figs. 15B, J; 16C?.

1989 *Neospathodus bransoni* (Müller); Thang, p. 417, pl. 29, fig. 10.

2009 *Guangxidella*? sp. A; Orchard & Zonneveld, p. 780, fig. 14, parts 33, 34.

2013 *Discretella discreta* (Müller); Yan et al., p. 516, fig. 6W.

Number of specimens: >15

*Diagnosis.* Segminate P_1_ element with rounded, upturned basal cavity at the posteriormost part. Large conspicuous cusp above basal cavity. Discrete, smaller upright denticles in the anterior part.

*Description.* These P_1_ elements have markedly discrete denticles and a large cusp. The lower margin is straight in the anterior and upturned at the posterior part. The basal cavity is drop-shaped, being rounded at the posterior edge and tapered at the anterior end. The cusp is relatively large and reclined. A small, accessory, posterior denticle is occasionally present.

*Remarks.* In comparison to *Discretella discreta*, this species has a rounded posterior margin of the basal cavity, more discrete denticles and the cusp is relatively larger and often recurved. In lateral view, the lower margin is upturned as in morphotype B of *Discretella discreta* (Maekawa & Igo, in Shigeta et al., 2014)*.*

*Occurrence*. Luolou formation, *Owenites* beds Smithian age, Shanggang road cut, Guangxi, South China.

*Discretella pseudodieneri* n. sp.

Figs. 15F, H, K, M, P; 16G–I, K, L, N, O

2013 *Neospathodus dieneri* Sweet; Yan et al., p. 516, fig. 6S (only).

2014 *Neospathodus dieneri* Sweet; Maekawa & Igo in Shigeta et al., p. 224, fig. 162, nr. 40–42, 46–54 (only).

2014 *Discretella* sp. indet. A; Maekawa & Igo in Shigeta et al., p. 202–207, fig. 151.13–151.15 (only).

2015 *Discretella* sp.; Chen et al., p. 112, fig. 8.12.

2018 *Neospathodus dieneri* Sweet; Maekawa in Maekawa et al., p. 26, fig. 15.32–15.34 (only).

2021 *Neospathodus* cf. *dieneri discreta*; Sun et al., fig. 5.10.

2021 *Discretella* sp. A; Sun et al., fig. 6.15.

*Etymology*: Named after its superficial resemblance to *Ns. dieneri*.

*Holotype:* specimen illustrated in Fig. 16O.

*Paratype:* specimen illustrated in Fig. 16H.

*Type locality:* Shanggang road cut, Luolou formation, Guangxi Province, China.

*Type level*: Luolou Formation, within the early-to-middle Smithian limestones (*Owenites* beds).

Number of specimens: >30

*Diagnosis.* Segminate P_1_ element, laterally flattened. Few, discrete, large denticles. Terminal cusp. Flat to slightly posteriorly upturned basal margin. Asymmetrical basal cavity.

*Description.* These P_1_ elements have markedly discrete denticles and a relatively large cusp, sometimes slightly larger than the other denticles that is situated terminally. In early ontogenetic stages (for adult forms, see Leu et al., submitted), the elements bear 3 erect to slightly and gradually reclined denticles. The basal margin is straight to slightly upturned posteriorly. In aboral view, the basal cavity is sub-rounded to lanceolate and asymmetrical.

*Remarks.* The P_1_ element of this species shows some superficial similarities with that of *Neospathodus dieneri*, but differs in being laterally flattened and having much less denticles (usually 3, vs. 5 to 10 in *Ns. dieneri*). Moreover, *N. dieneri* ranges up to the early Smithian only, whereas *D. pseudodieneri* occurs in the middle Smithian *Owenites* beds. Included here are also specimens where the cusp is less discrete, more robust and broader at the base (Figs. 15M, 16L).

*Occurrence*. Tahogawa Member, Olenekian *Nv*. ex gr. *waageni* Zone, Japan (Maekawa et al., 2018). Jiarong, *Discretella discreta* Zone, Smithian age Nanpanjiang basin, southern Guizhou, South China (Chen et al., 2015).

*Discretella*? n. sp. B

Figs. 15N; 16B

Number of specimens: >10

*Diagnosis.* Segminate P_1_ element with subsymmetrical, posteriorly rounded and anteriorly tapered basal cavity. Large terminal, subtriangular, recurved cusp. Anterior process bearing a few denticles only.

*Description.* The element shows a large cusp at the posterior end. In lateral view, the large, recurved, subtriangular cusp is about as high as the whole unit is long. An anterior process with two or three small upright to reclined denticles is present. The lower margin is straight to concave. In aboral view, the subsymmetrical basal cavity is sub-rounded at the posterior margin and tapers towards the anterior end.

*Remarks.* These P_1_ elements most closely resemble *Discretella* aff. *discreta*, but differ in having a much shorter anterior process and recurved denticles. They also bear some superficial resemblance with the P_1_ elements of the Spathian–Anisian genus *Cornudina*, but differ in their conformation of the basal cavity. *Urdyella unicorna* n. gen. n. sp. usually has a much thinner and larger, sickle-shaped cusp and a rounded basal cavity.

Because of the absence of a posterior process, some elements, especially arched ones, resemble homologous elements of *Guangxidella* and could possibly be assigned to that genus, but their basal cavity do not match those of *Guangxidella* and resemble instead that of *Discretella* aff. *discreta*, hence we tentatively retain them within *Discretella*.

*Occurrence*. South China: Luolou, Smithian age, Laren, Guangxi.

*Discretella*? n. sp. C

Figs. 15O; 16A, F, M.

1989 *Cratognathodus* sp. A; Thang, p. 405, pl. 32, fig. 8.

2013 *Neospathodus*? *peculiaris* Sweet; Yan et al., p. 516, fig. 6M.

2014 *Discretella* sp. indet. A; Maekawa & Igo in Shigeta et al., p. 202–207, fig. 151.1–151.12, 151.16–151.18 (only).

2014 Genus gen. indet. D; Maekawa & Igo in Shigeta et al., p. 269, figs. 193.10–193.12.

Number of specimens: >10

*Diagnosis and description.* Same as *Discretella*? n. sp. B except that a very small posterior process without denticle is always present and the laterally twisted basal cavity tapers at both ends. The anterior process may bear up to 5 denticles.

*Remarks.* The P_1_ elements of this species are very similar to those of the coeval *Discretella*? n. sp. B and both species may be conspecific although the presence of the incipient posterior process and the geometry of the basal cavity suggest they correspond at least to two different morphotypes, possibly due to sexual dimorphism. They also share strong similarities with the P_1_ of *Discretella?* n. sp. D, which may be a descendant of *Discretella*? n. sp. B via reduction/loss of the anterior process, forms like, Fig. 16M (and figs. 193.10–193.12 of Maekawa and Igo in Shigeta et al., 2014) that bear only one or two anterior denticles being intermediary. The relationship with *Discretella* sp. indet. A Maekawa & Igo is unclear but the P_1_ of the latter is almost identical, except that the incipient posterior process bears a small triangular denticle. Maekawa & Igo noted also that the anterior process of the P_1_ element of *Discretella* sp. indet. A (Maekawa & Igo) may also have been reduced over time. Conversely, *Discretella robustus* (Wang & Wang, 1976), which share a similar recurved cusp and a sigmoidal profile in lower view, has more developed anterior and posterior processes and may be their forerunner.

*Occurrence*. South China: Luolou, Smithian age, Laren, Guangxi.

*Discretella*? n. sp. D

Fig. 26AB, AC.

**Fig. 26:**
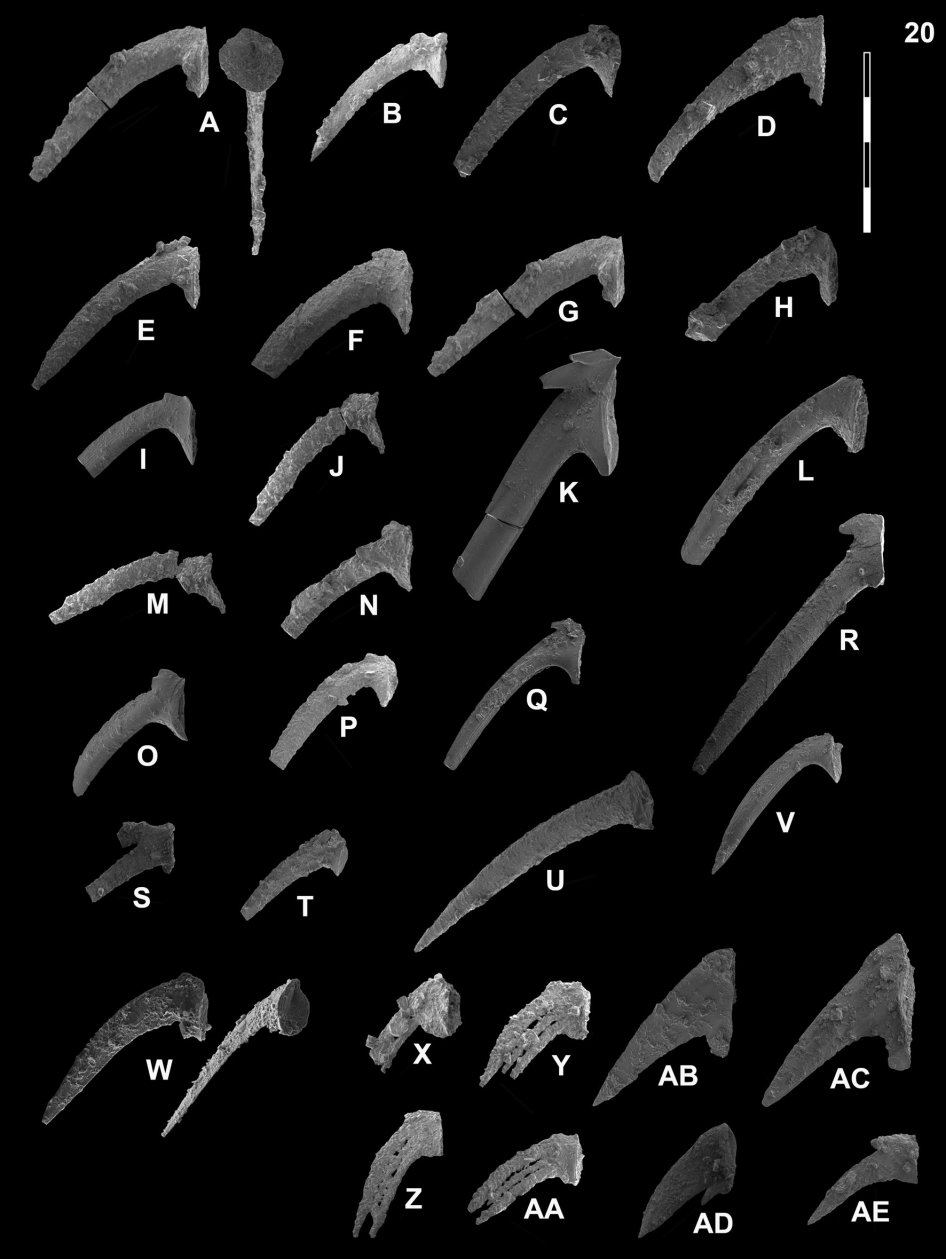
Cornudininae from Qiakong, Laren and Shanggang. Magnification is × 80. The scale bar is 400 μm. All elements are considered to be P_1_ elements if not specifically identified otherwise. **A**–**W** *Urdyella unicorna* n. gen. n. sp.; **A** LAR225, PIMUZ 39345; **B** LAR225, PIMUZ 39346; **C** LAR229, PIMUZ 39347; **D** LAR227, PIMUZ 39348; **E** LAR229, PIMUZ 39349; **F** LAR229, PIMUZ 39350; **G** LAR225, PIMUZ 39351; **H** LAR229, PIMUZ 39352; **I** QIA123, PIMUZ 39353; **J** LAR227, PIMUZ 39354; **K** QIA123, PIMUZ 39355; **L** QIA123, PIMUZ 39356; **M** LAR227, PIMUZ 39357; **N** LAR225, PIMUZ 39358; **O** QIA120, PIMUZ 39359; **P** LAR225, PIMUZ 39360; **Q** QIA121, PIMUZ 39361; **R** LAR232, PIMUZ 39362; **S** LAR232, PIMUZ 39363; **T** QIA124, PIMUZ 39364; **U** LAR232, PIMUZ 39365; **V** QIA123, PIMUZ 39366; **W** SHA341, PIMUZ 39367. **X-AA** *Urdyella tridenta* n. gen. n. sp.; **X** LAR227, PIMUZ 39341; **Y** LAR227, PIMUZ 39342; **Z** LAR227, PIMUZ 39343; **AA** LAR227, PIMUZ 39344. **AB**, **AC** *Discretella*? n. sp. D; **AB** QIA124, PIMUZ 39118; **AC** QIA123, PIMUZ 39119. **AD** *Neostrachanognathus* n. sp. A; LAR232, PIMUZ 39183. **AE** *Urdyella* n. sp. A; LAR232, PIMUZ 39340

2014 Genus gen. indet. D; Maekawa & Igo in Shigeta et al., p. 269, figs. 193.8–9.

*Holotype.* Specimen illustrated in Fig. 26AB.

Number of specimens: >20

*Diagnosis.* Same P_1_ element as in *Discretella*? n. sp. C except it bears no anterior process.

*Remarks.* As for *Discretella*? n. sp. B and C (see above) the generic assignment of these forms is uncertain and may deserve a new genus name, especially given that the present taxa bear neither posterior nor anterior denticles.

*Occurrence*. Laren road cut, Luolou Formation, Smithian age, Nanpanjiang basin, South China (this paper); North-eastern Vietnam, *Novispathodu*s ex gr. *waageni* Zone between *Flemingites rursiradiatus* beds and Urdyceras tulongensis beds and within the *Owenites koeneni* beds, Bac Thuy Formation (Shigeta et al., 2014).

Genus GUANGXIDELLA Zhang & Yang, 1991

*Type species*. *Neoprioniodus bransoni* Müller 1956, p. 829, pl. 95, figs. 19–21.

*Type stratum and locality*. Smithian ammonoid bed, Crittenden Springs, Elko County, Nevada.

*Guangxidella bransoni* (Müller, 1956)

Figs. 15E; 16E.

*1956 *Neoprioniodus bransoni* n. sp.; Müller, p. 829, pl. 95, figs. 19–21.

1956 *Neoprioniodus bicuspidatus* n. sp.; Müller, p. 828, pl. 95, figs. 16, 17.

1979 *Neospathodus bicuspidatus* (Müller); Solien, p. 302, pl. 3, figs. 2, 3.

1989 *Ozarkodina gigantea* n. sp.; Thang, p. 409, pl. 31, figs. 10, 14.

1991 *Guangxidella typica* n. sp.; Zhang & Yang, p. 33, pl. 1, figs. 1a, b, 2a, b.

2009 *Guangxidella bransoni* (Müller); Orchard & Zonneveld, p. 780, fig. 15, parts 26–28.

2014 *Guangxidella bransoni* (Müller); Maekawa & Igo in Shigeta et al., p. 211, figs. 152.16–152.18, 153–159, 160.1–160.4

2018 *Guangxidella bransoni* (Müller); Maekawa in Maekawa et al., p. 24, figs. 14.7–14.10.

Number of specimens: >30

*Description.* The segminate P1 element has variably discrete, laterally compressed denticles whose height decreases to the anterior and a very large, variably reclined, terminal cusp. The basal margin is up-arched and the basal cavity is flat, cordiform and asymmetrical.

*Remarks. Gu. bicuspidatus* was differentiated from *Gu. bransoni* on the basis of its closer spaced denticles, the one in front of the cusp being almost as high as the latter (Müller, 1956). Yet, given the broad range of intraspecific variation present in large collections such as those illustrated by Shigeta et al. (2014), it is in our opinion no longer tenable to keep both species separated. Although both taxa are defined in the same paper by Müller (1956) and the definition of *Gu. bicuspidatus* appears before that of *Gu. bransoni*, we have decided to name them *bransoni* because the etymology of *bicuspidatus* refers to a 2-cusp element, which is not typical of the revised species. Furthermore, Müller’s holotype of *Gu. bicuspidatus* is broken, whereas his holotype of *Gu. bransoni* is better preserved (not broken), better illustrated (also in aboral view) and corresponds better to the revised type.

*Occurrence*. USA: Dinner Springs Canyon, Lower Triassic *Meekoceras* beds, Crittenden Ranch, Elko County, Nevada (Müller, 1956); Thaynes Formation (*Parachirognathus* Zone, Utah, (Solien 1979). British Columbia, Canada (Orchard & Zonneveld, 2009). Taho Formation, *Novispathodus* ex. gr. *waageni* Zone) Southwest Japan (Maekawa et al., 2018). Nanpanjiang basin: Bac Thuy Formation, *Owenites* beds within the *Novispathodus* ex. gr. *waageni* Zone, North-eastern Vietnam (Shigeta et al., 2014). Luolou Formation, *Neospathodus waageni* Zone, Guangxi, China (Zhang & Yang, 1991).

Subfamily CORNUDININAE Orchard 2005

Genus SPATHICUSPUS Orchard 2005

*Type species. Neospathodus spathi* Sweet, 1970, pp. 257–258, pl. 1, fig. 5.

*Type stratum and locality.* Mittiwali Member, Mianwali Formation, Narmia, Pakistan.

*Remarks.* Based on his reconstruction of the multi-element apparatus of ‘Neospathodus’ spathi Orchard (2005) considered these taxa deserved assignment not only to a new genus, but also to a new subfamily.

*Spathicuspus* n. sp. A

Fig. 13M, P

2005 *Neospathodus spathi* Sweet; Gaetani et al., p. 288, pl. 1, fig. 2

2015 *Spathicuspus spathi* (Sweet); Chen et al., p. 112, fig. 8.16–8.17.

2015 *Spathicuspus spathi* (Sweet); Lehrmann et al., p. 123, fig. 5.28.

2016 *Spathicuspus spathi* (Sweet); Liang et al., p. 385, fig. 4.10.

Number of specimens: >10

*Description.* Short segminate P1 element with large, terminal, reclined or recurved cusp. Only two anterior denticles. Basal cavity is drop-shaped.

*Remarks.* Very similar to *Spathicuspus*? n. sp. B (see below), but much shorter and with a larger basal cavity. Similar elements have been illustrated from other Chinese sections by Chen et al. (2015) and Liang et al. (2016) and hence this species may be useful as an index fossil for intrabasinal correlations.

*Occurrence*. South China: Luolou Formation, Spathian, Nanpanjiang basin, Mingtang section, (Liang et al., 2016) Upper Guandao section, (Lehrmann et al., 2015), *Triassospathodus homeri* zone, Jiarong (Chen et al., 2015).

*Spathicuspus*? n. sp. B

Fig. 13J–L, O (N, R juvenile forms?)

2005 *Spathicuspus* sp. A; Orchard, p.77, fig. 2, Nr. A.

2016 *Spathicuspus spathi* (Sweet); Liang et al., p. 385, fig. 4.9.

2019 *Spathicuspus spathi* (Sweet); Chen et al., fig. 7, nr. 6–8.

2021 *Spathicuspus spathi* (Sweet); Chen et al., fig. 7.8 (only).

Number of specimens: >30

*Description.* Slender segminate P_1_ element with a terminal, reclined or recurved cusp that is usually broader than adjacent denticles, a shallow, posteriorly rounded basal cavity and three to seven anterior denticles.

*Remarks.* Despite their overall resemblance with *Spathicuspus spathi* these elements are either too long and bear too many denticles or their cusp is not broad and blunt enough to be assigned to that species. Some of them may not belong to *Spathicuspus* at all. Similar and apparently coeval elements were illustrated by Liang et al. (2016) and Chen et al. (2019) from other sections in China and Oman. The element illustrated by Orchard (2005) has similar aspect ratio and denticulation as for instance the specimen we illustrated in Fig. 13O, but it is also much younger and may be only superficially similar.

*Occurrence*. South China: Luolou Formation, Spathian, Mingtang section, Nanpanjiang basin (Liang et al., 2016), Upper Guandao section (Orchard, 2005). Oman: Wadi Bani Khalid section, Spathian UAZ_7_ (Chen et al., 2019).

F13

*Spathicuspus*? n. sp. C

Fig. 13Q

2016 *Spathicuspus spathi* (Sweet); Liang et al., p. 385, fig. 4.11.

2021 *Spathicuspus spathi* (Sweet); Chen et al., fig. 7.5 (only).

Number of specimens: >5

*Description.* Small P1 element with a rounded basal cavity, a large, reclined and broad cusp, a small posterior denticle and a couple of declining denticles to the anterior.

*Remarks.* Although the relationship of this species to *Spathicuspus spathi* and *Spathicuspus* n. sp. A is unclear, its shape is intermediate. It differs in the presence of a posterior denticle.

*Occurrence*. South China: Luolou Formation, Spathian, Mingtang section, Nanpanjiang basin (Liang et al., 2016).

Genus URDYELLA gen. nov.

*Type species. Urdyella unicorna* n. sp.

*Type stratum and locality.* Luolou Formation, Laren road cut, Guangxi Province, China.

*Etymology*: Named after Severine Urdy.

*Diagnosis*. Short coniform-to-segminiscaphate P_1_ element with a long cusp, usually at least twice the length of the adjacent denticle, a very short anterior process, and a broadly excavated basal cavity.

*Remarks*. The relationship of this genus to *Cornudina* is uncertain (Orchard, 2005, 2007). P_1_ elements similar to the P_1_ element of *Urdyella* n. gen. are often considered as belonging to *Cornudina.* Yet, as explained by Orchard (2005), the holotype of *Cornudina* (*O. breviramulus*) appears to be a P_2_ element. Both Kozur and Mostler (1971, p. 11) and Sweet (in Clark et al., 1981, p. W155) placed *Cornudina* with *Chirodella* as a multi-element *Chirodella*. The holotype of *Chirodella* (*Metalonchodina triquetra*) is an S_2_ element. The holotypes of both *Cornudina* and *Chirodella* are from Muschelkalk (Middle Triassic) collections made by Tatge (1956), who described no elements like those described by Orchard (2005) as P_1_. ‘*Chirodella’* sensu formo does not occur in Orchard’s nor in Koike’s Spathian collections of *Cornudina*. For this reason, Koike (1996) and Orchard (2005) regarded the two genera as unrelated: Koike reconstructed *Cornudina* as an apparatus consisting of P_1_ and P_2_ elements only (*Cornudina breviramulis*) or of P_1_ elements only (*Cornudina igoi*), whereas Orchard reconstructed *Cornudina*? as having an octomembrate apparatus whose P_2_ element vaguely resembles the holotype of *Cornudina*, but he questioned the very validity of that name for Spathian forms that might be unrelated to the ‘true’ Middle Triassic *Cornudina*.

‘*Chirodella’* sensu formo does not occur in our Smithian collections of *Urdyella* n. gen. and neither does ‘*Cornudina’* sensu formo. Hence we suggest that *Cornudina*-like P_1_ elements found in the Smithian, and possibly those found in the Spathian, belong to *Urdyella* n. gen. not to *Cornudina.*

Based on multi-element considerations, Orchard grouped both ‘*Cornudina’* and *Spathicuspus* within the same subfamily Cornudininae. Because *Spathicuspus* seemed to occur first, he hypothesized that ‘*Cornudina’* evolved from *Spathicuspus* near the Lower/Middle Triassic boundary through overall shortening of the P_1_ element. Since *Urdyella* occurs already in the Smithian, we suggest that *Spathicuspus* may have evolved from *Urdyella* and the Spathian forms of ‘*Cornudina’* either belong to *Urdyella,* derived directly from *Urdyella,* or as assumed by Orchard, evolved from *Spathicuspus*, although the latter hypothesis seems less likely. *Urdyella* itself may have evolved from *Discretella* via forms like *Discretella*? n. sp. B. *Discretella* has a much shorter cusp and a larger anterior process. Furthermore, the basal cavity in *Urdyella* is flatter and not as broadly excavated as in *Cornudina*.

*Urdyella unicorna* n. sp.

Fig. 26A–W, AE?

1970 *Neospathodus peculiaris* Sweet?; Hasenmüller, p. 62, pl. 2, fig. 10

2005 *Aduncodina unicosta* Ding; Zhao, pl. 6, nr. 9–10.

*Etymology*: Named after the unicorn, a mythical creature with a single horn on its forehead, for the huge cusp of the P_1_ element of this species.

*Holotype:* specimen illustrated in Fig. 26A.

*Paratypes:* specimens illustrated in Fig. 26K, Q, W.

*Type locality:* Laren road cut, Luolou formation, Guangxi Province, China.

*Type level*: Luolou Formation, upper *Owenites* to lower *Anasibirites* beds, middle Smithian to early late Smithian, Olenekian, Early Triassic_._

*Number of specimens*. > 40.

*Diagnosis.* P_1_: Short coniform-to-segminiscaphate element with a very long, needle-like, recurved cusp, sometimes one (or two in large specimens) tiny, anterior denticle, and a broadly excavated basal cavity.

*Description*. P_1_: The cusp is about 3–4 times longer than the basal cavity is broad, is usually slightly recurved, sometimes only reclined, and it makes an angle of about 40°–60° with the baseline of the lower margin in lateral view. Many specimens are coniform and may bear a tiny anterior process, some (usually larger) specimens have a very short anterior process bearing up to two anterior denticles and may be better described as segminiscaphate. The deep basal cavity is oval to drop-shaped in aboral view. In lateral view, the base of the cusp occupies the anterior half of the basal cup.

*Remarks.* Specimens of this species were found by Zhao et al., (2007, without illustrations; but two specimens are illustrated in Zhao’s Ph.D thesis, 2005) in coeval strata of the Chaohu section, Anhui Province but reported as *Aduncodina unicosta*, a possibly related form that in all our collections from China is otherwise restricted strictly to the Spathian interval.

*Occurrence.* India: Mud, Mikin Formation, maximal horizon 1 (Goudemand, 2011); Western USA, *Parachirognathus-Furnishius* zone, Thaynes Formations, Utah (Hasenmüller, 1970). South China: Chaohu section, Anhui Province (Zhao, 2005; Zhao et al., 2007); Pingtang Syncline, uppermost Daye Formation and Luolou Formation, Smithian (this paper). Upper Smithian, Khunamuh Formation, Guryul Ravine, Northern Indian Margin (Leu et al. in prep.), Oman (Leu et al. submitted).

*Urdyella tridenta* n. sp.

Fig. 26X-AA

*Etymology*: The species is named after the trident, Poseidon’s weapon in the Greek mythology.

*Holotype.* Specimen illustrated in Fig. 26AA.

*Paratype.* Specimens illustrated in Fig. 26Z.

*Number of specimens*. 5

*Diagnosis.* Coniform P_1_ element with three subequally long and slightly recurved denticles all attached to the basal cup. Deep, teardrop shaped basal cavity. No clear anterior process.

*Remarks.* The P_1_ element of this species is very similar to the one of *Urdyella unicorna* n. sp. except for the lack of an anterior process and the fact that the single large cusp of *Urdyella unicorna* n. sp. is replaced by three subequally long denticles in *Urdyella tridenta* n. sp.

*Occurrence*. Laren road cut, Luolou Formation, Smithian age, Nanpanjiang basin, South China (this paper).

*Urdyella* n. sp. A

Fig. 26AE

*Number of specimens*. 1

*Remarks*. This one specimen does look like it may belong to *Urdyella* and it was found within the Smithian range of *Urdyella,* but it differs in its posterior configuration and in having a smaller cusp.

*Occurrence.* Laren road cut, Luolou Formation, Smithian age, Nanpanjiang basin, South China (this paper).

Genus NEOSTRACHANOGNATHUS Koike 1998

*Type species. Neostrachanognathus tahoensis* Koike 1998,

*Type stratum and locality.* The Taho Limestone, Ehime, Japan.

*Neostrachanognathus* n. sp. A

Fig. 26AD?

Number of specimens. 1

*Remarks*. That one specimen looks like it could be a P element of *Neostrachanognathus* as reconstructed by Agematsu et al. (2008). Since it possesses a posterior denticle, it does not belong to *Neostrachanognathus tahoensis* and may instead belong to *Neostrachanognathus* sp. A (Agematsu et al., 2008) or to a different species. This element was found in middle Smithian strata together with representatives of *Urdyella* spp. If it does indeed belong to *Neostrachanognathus* it is its oldest representative.

*Occurrence*. Laren road cut, Luolou Formation, Smithian age, Nanpanjiang basin, South China.

Genus ADUNCODINA Ding, 1983

*Type species. Aduncodina unicosta* Ding, 1983.

*Type stratum and locality.* Biandanshan formation, Mountain Majiashan of Chaoxian, Anhui province, China.

*Aduncodina unicosta* Ding, 1983

Fig. 24R–T

*1983 Aduncodina unicosta n. sp.; Ding, p.41, pl. 6, figs. 10–14, 20(?)-21.

1983 *Cornudina* cf. *oezdemirae* Gedik; Ding, p. 42, pl. 7, figs. 25–26

1998 *Aduncodina unicosta* Ding; Koike, p. 126, fig. 8.4–8.26.

2007 *Aduncodina unicosta* Ding; Orchard, p. 96, fig. 2

2019 *Aduncodina unicosta* Ding; Chen et al., figs. 5.7, 5.9, 6.6.

*Number of specimens. *> 30.

*Description*. *Aduncodina unicosta* has been reconstructed by Koike (1998) as a quadrimembrate skeletal apparatus encompassing only S_a–c_ (S_1–3_) and M nongeniculate coniform elements. The adenticulated, subsymmetrical element is regarded by Koike as the M element, whereas the denticulated elements are interpreted as pertaining to the S series, the subsymmetrical one that bears one denticle being the S_a_ (S_0_), the asymmetric one that bears up to three antero-lateral, hook-like denticles (in Zhao’s collections, those elements may bear up to five denticles, see his Ph.D thesis, 2005) and whose basal cavity is triangular in cross-section being the S_b_ (S_1_) and the asymmetric one with a lenticular basal cavity being the S_c_ (S_2_). All elements share common characteristics such as a thin wall, a relatively large and long and laterally flattened basal cavity. The slender cusp is suberect and subcircular in cross-section. Basal margin weakly to strongly convex anteriorly.

*Remarks*. We agree with Orchard (2007) that the reconstruction by Koike (1998) might be incomplete. *Neostrachanognathus* was also reconstructed by Koike (*ibid*) as including only coniform elements but later revised by Agematsu et al. (2008) with convincing evidence from natural assemblages as including also complex multidenticulate (bipennate) elements in the S positions. It is to be expected that *Aduncodina* too may possess complex multidenticulate elements in those positions. The morphology of the illustrated elements of *Aduncodina unicosta* resembles that of Ordovician forms such as *Strachanognathus*. In common with *Neostrachanognathus*, *Aduncodina* has a suite of denticulate elements, identified as S_b_ and S_c_ by Koike (1998), with an antero-lateral denticle or process that is directed posteriorward at its base, which is a feature of the Cornudininae (Orchard, 2007).

*Occurrence*. Japan: Taho Formation, co-occurrence with *Ic*. *collinsoni* in the Spathian substage (Koike, 1998); China: Biandanshan formation, co-occurrence with *Ic*. *collinsoni* and *Tr*. *homeri*, Mountain Majiashan of Chaoxian, Anhui province (Ding, 1983). Oman: Wadi Bani Khalid section, Spathian between UA_10_ and UA_11_ (Chen et al., 2019).

Subfamily UNCERTAIN

Genus EURYGNATHODUS Staesche 1964.

*Type species. Eurygnathodus costatus* Staesche, 1964

*Type stratum and locality.* Campiller member, Skyth, South Tirol, Italy.

*Description*. Scaphate P_1_ element bearing a variably broad, oval-shaped platform with or without transverse ribs. So far, the multi-element apparatus of *Eurygnathodus* is unknown.

*Remarks*. Recent discussions for the definition of the Global Stratotype Section and Point (GSSP) for the Induan–Olenekian Boundary (IOB) emphasize that this easily identifiable and cosmopolitan genus may be a useful index for the base of the Olenekian. Yet, its phylogenetic origin is still unclear, leaving opened the question of whether its seemingly sudden appearance in equatorial and tropical localities close to the IOB may reflect an ecological signal.

*Eurygnathodus costatus* Staesche, 1964

Fig. 14A

*1964 *Eurygnathodus costatus* n. sp.; Staesche, p. 269, pl. 28, figs. 1–6.

1977 *Platyvillosus costatus* (Staesche); Goel, p. 1098, pl. 2, figs. 15–21.

1981 *Platyvillosus costatus* (Staesche); Wang & Cao, p. 371, pl. 2, figs. 1–4, 28–30, 33.

1981 *Platyvillosus paracostatus* n. sp.; Wang & Cao, p. 371, pl. 2, figs. 9,10.

1984 *Platyvillosus costatus* (Staesche); Matsuda, p. 128, pl. 6, figs. 6–10.

1988 *Platyvillosus costatus* (Staesche); Koike, p. 65, pl. 1, figs. 1–57, pl. 2, figs. 1–37.

1991 *Platyvillosus costatus* (Staesche); Beyers & Orchard, pl. 5, fig. 10.

2009 *Eurygnathodus costatus* Staesche; Igo in Shigeta et al., p. 183, figs. 152.23–152.24.

2010 *Eurygnathodus costatus* Staesche; Orchard, p. 145, fig. 5.9–5.10.

2013 *Platyvillosus hamadai* Koike; Zhao et al., p.535, figs. 10K, L.

2013 *Platyvillosus costatus* (Staesche); Zhao et al., p.535, figs. 10M, 10N, 10O.

2014 *Eurygnathodus costatus* Staesche; Maekawa & Igo in Shigeta et al., p. 220, figs. 161.4–161.6

2015 *Eurygnathodus hamadai* Staesche; Maekawa in Maekawa et al., p. 316, fig. 5.1.

2016 *Eurygnathodus costatus* Staesche; Chen et al., fig. 11.3, 11.6–11.7.

**Fig. 27:**
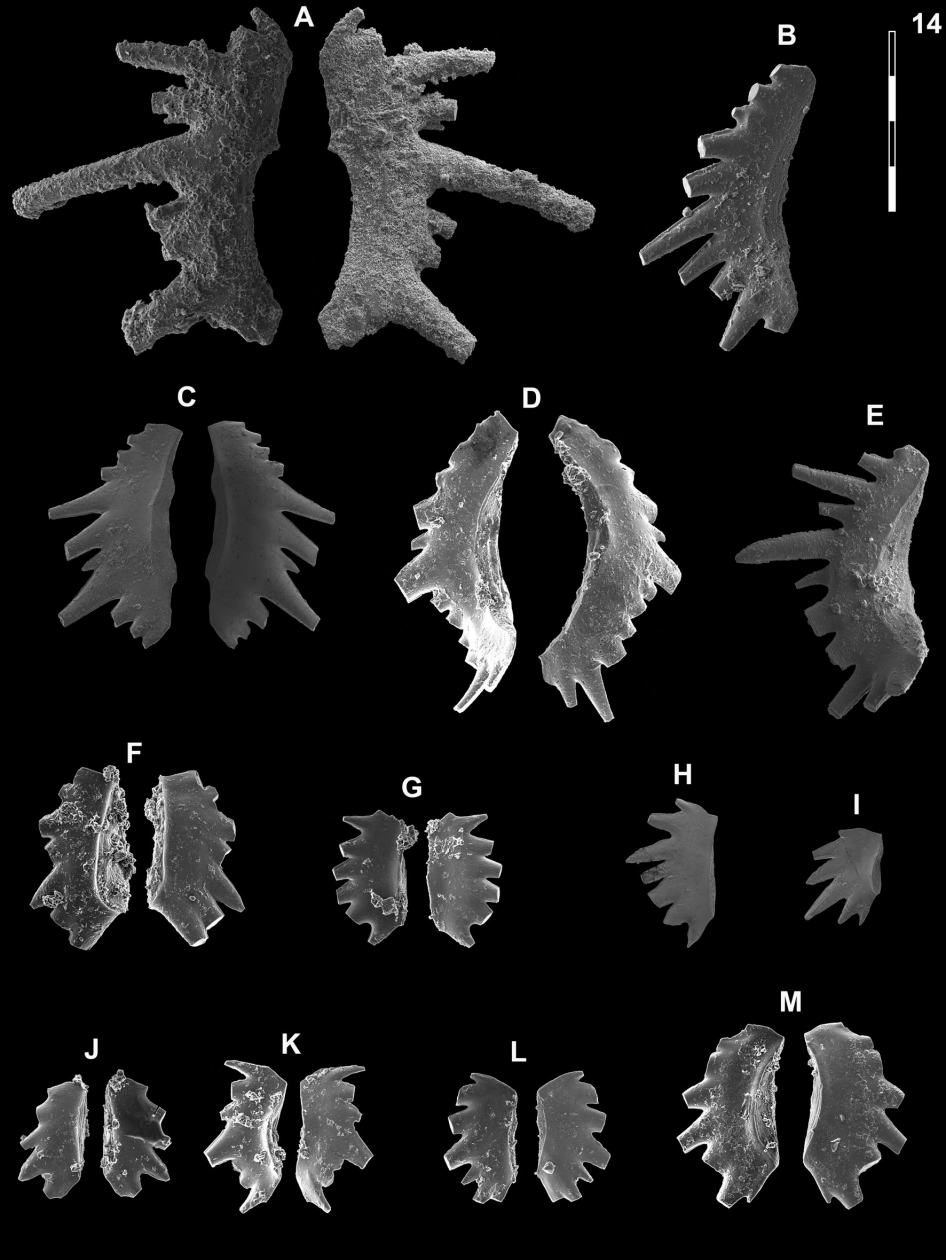
Hadrodontinae from Qiakong, Laren, Shanggang and Lilong. Magnification is × 80. The scale bar is 400 μm. All elements are considered to be P_1_ elements if not specifically identified otherwise. **A**, *Hadrodontina aequabilis* (S_2_ element) (Staesche); SHA344, PIMUZ 39141. **B**–**E**, *Hadrodontina aequabilis* (P_2_ element) (Staesche); **B** LAR214, PIMUZ 39142; **C** QIA13, PIMUZ 39143; **D** LIL500, PIMUZ 39144, **E** SHA344, PIMUZ 39145. **F**–**M** *Hadrodontina aequabilis* (Staesche); **F** LIL502, PIMUZ 39146; **G** LIL503, PIMUZ 39147; **H** QIA134, PIMUZ 39148; **I** QIA134, PIMUZ 39149; **J** QIA 133, PIMUZ 39150; **K** LIL502, PIMUZ 39151; **L** LIL501, PIMUZ 39152; **M** LIL501, PIMUZ 39153

2018 *Eurygnathodus costatus* Staesche; Maekawa in Maekawa et al., pp. 45–50, figs. 25–27.

2019 *Eurygnathodus costatus* Staesche; Li et al., p. 6, fig. 4.22–4.36.

2019 *Eurygnathodus costatus* Staesche; Lyu et al., fig. 7.12.

2019a *Eurygnathodus costatus* Staesche; Wu et al., fig. 4.20.

Material. > 30 specimens.

*Remarks*. Goel (1977), Matsuda (1984), Koike (1988) and Maekawa et al. (2018) documented a large intraspecific variation of the platform morphology and its oral ornamentation. It follows that *Platyvillosus paracostatus* Wang and Cao (1981) likely corresponds to a variant of *Eu. costatus* (Form A in Koike 1988). Maekawa et al., (2018, figs. 25.1d) documented also specimens covered with microgranules.

*Occurrence.* This species has been reported worldwide from the early Smithian *Flemingites* zone. Japan: Tahogawa member within the *Novispathodus* ex gr. *waageni* Zone (Koike, 1988; Maekawa et al., 2018), Europe: South Tyrol, Slovenia, Croatia and Bosnia and Herzegovina (Aljinović et al., 2006; Chen et al., 2016; Kolar-Jurkovšek et al., 2021; Staesche, 1964), India: Spiti and Kashmir (Goel, 1977; Matsuda, 1984; Orchard, 2010; Orchard & Krystyn, 2007), Russia; South Primorye (Shigeta, 2009), Canada: British Columbia (Beyer & Orchard, 1991), South China and North-eastern Vietnam (Chen et al., 2015; Shigeta et al., 2014).

*Eurygnathodus hamadai* (Koike, 1982)

Fig. 14B

1981 *Platyvillosus costatus* (Staesche); Wang and Cao, p. 371, pl. 2, figs. 31–32.

*1982 *Platyvillosus hamadai* n. sp.; Koike, p.45, pl. 5, figs. 10–36.

1988 *Platyvillosus hamadai* Koike; Koike, p. 71, pl. 2, figs. 38–45.

2010 *Eurygnathodus hamadai* (Koike); Orchard, p. 145, fig. 5.11.

2015 *Eurygnathodus hamadai* (Koike); Maekawa in Maekawa et al., p. 317, fig. 5.2.

2018 *Eurygnathodus hamadai* (Koike); Maekawa in Maekawa et al., p. 50, figs. 28.1–28.4.

2019 *Eurygnathodus hamadai* (Koike); Li et al., p. 6, figs. 4.37–4.45.

2019a *Eurygnathodus hamadai* (Koike); Wu et al., fig. 4.22.

Material. > 30 specimens.

*Remarks. Eurygnathodus hamadai* is easily distinguished from *Eurygnathodus costatus* by its smooth, flat upper surface lacking ornamentation. Koike (1988) reported transitional forms between *E*. *costatus* and *E*. *hamadai* (Morphotype δ), raising the question whether both forms may be conspecific, *E*. *hamadai* representing an extreme variant of *E*. *costatus.* In our material and in that of other authors, *Eu. costatus* and *Eu hamadai* co-occur in several samples. Their relative abundance, however, does change. *Eu. costatus* is more common in older strata, whereas *Eu. hamadai* is more abundant than *Eu. costatus* in younger strata. Although the FO of *Eu. costatus* appears to predate that of *Eu. hamadai* in several sections around the world, it is thus still unclear whether they share the same temporal range or not.

*Occurrence.* early Smithian in China (Wang & Cao, 1981, this study), Malaysia (Koike, 1982), Japan (Koike, 1988; Maekawa et al., 2018, and India, Spiti (Orchard, 2010).

Suborder PRIONIODININA Donoghue et al., 2008

Family ELLISONIDAE Clark, 1972

Subfamily HADRODONTINAE Koike, 2016

Genus HADRODONTINA Staesche, 1964

*Type species. Hadrodontina anceps* Staesche, 1964.

*Type stratum and locality.* Campiller member, Skyth, South Tirol, Italy.

*Remarks*. There is still an ongoing debate about the phylogenetic relationships of *Pachycladina*, *Parapachycladina* and *Hadrodontina*. Sweet (1988) in his prioniodinid phylogeny considered *Pachycladina* and *Hadrodontina* as sister taxa. Some species of *Pachycladina* were assigned to a new genus *Parapachycladina* by Shunxin et al. (1997), but this view is not widely accepted. Orchard (2007) observed that *Hadrodontina anceps*, *Ellisonia* aff. *triassica* and *Pachycladina peculiaris* appear to constitute a natural group, although they are currently assigned to different genera. Based on their cladistics analysis, Donoghue et al. (2008) concluded that *Pachycladina* is either a sister taxon to *Ellisonia* or stays unresolved in a polytomy with *Ellisonia, Hadrodontina* and *Furnishius*. Based on his multi-element apparatus reconstructions, Koike (2016) included *Hadrodontina* and *Pachycladina* within the subfamily Hadrodontinae (Koike, 2016), supporting the original view of Sweet (1988). We follow here this suprageneric classification and include *Hadrodontina* and *Pachycladina* within the subfamily Hadrodontinae. More recently, Sun et al. (2020) published 3 natural assemblages of *Hadrodontina aequabilis* and confirmed the suprageneric classification of Koike (2016).

In P_1_ elements, what most distinguishes *Pachycladina* from *Hadrodontina* is the basal configuration: the inverted basal ‘attachment’ surface of *Pachycladina* occupies the entire lower side plus one lateral side of the carina, whereas in *Hadrodontina*, a basal cavity with a deep basal groove is usually formed on the lower side and the attachment surface rarely extends over the mid-part of the keel, if at all. The denticles of *Pachycladina* are also less numerous but much larger in relative size than those of *Hadrodontina*. Based on multi-element reconstructions however, Koike (2016) showed that *Pachycladina peculiaris*, as well as *Ellisonia* aff. *triassica* (Koike et al., 2004), should be synonymized with *Hadrodontina aequabilis.*

*Hadrodontina aequabilis* Staesche, 1964

**Fig. 28:**
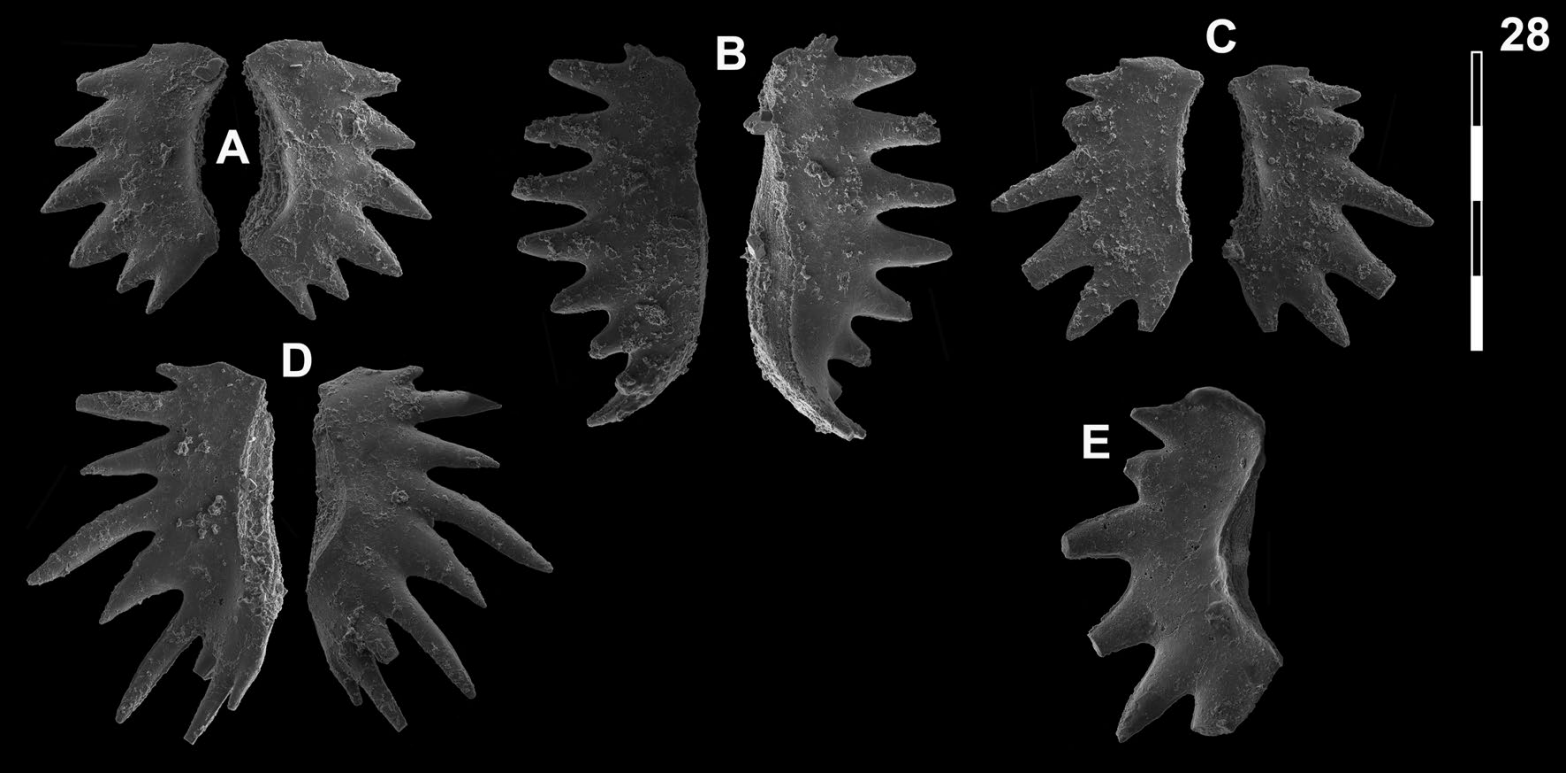
Hadrodontinae from Shanggang. Magnification is × 80. The scale bar is 400 μm. All elements are considered to be P_1_ elements if not specifically identified otherwise. **A**, **C**, **E** *Hadrodontina aequabilis* (Staesche); **A** SHA 345C, PIMUZ 39154; **C** SHA345C, PIMUZ 39155; **E** SHA344C, PIMUZ 39156. **B**, **D** *Hadrodontina aequabilis* (P_2_ element) (Staesche); **B** SHA345C, PIMUZ 39157; **D** SHA345C, PIMUZ 39158

P_1_ elements: Figs. 27F–M; 28A, C, E

P_2_ elements: Figs. 27B–E; 28B, D

S_2_ elements: Fig. 27A

P_1_ element:

*1964 Hadrodontina aequabilis n. sp.; Staesche, p. 275, figs. 43, 44.

1984 *Sweetocristatus unicus* n. sp.; Dagis, pp. 37–38, pl. X, figs. 6–9.

1990 *Pachycladina peculiaris* n. sp.; Shunxin (Zhang), pl. 2, fig. 4

1991 *Pachycladina peculiaris* n. sp.; Zhang in Zhang & Yang, p. 40, pl. 3, figs. 1, 2.

1997 *Parapachycladina peculiaris* Zhang; Shunxin et al., pp. 65–69, pl. 1, figs. 1–2, pl. 2, figs. 1–2, pl. 3 figs. 1–2.

2004 *Ellisonia* sp. aff. *E. triassica* Müller, 1956; Koike et al., figs. 8.7, 8.8.

2009 *Ellisonia*? cf. *peculiaris* Sweet; Igo in Shigeta et al., p. 182, fig. 152.22.

2013 *Parachirognathus geiseri* Clark; Yan et al., p. 516, fig. 6 FF.

2015 *Parachirognathus peculiaris* Zhang & Guo; Chen et al., figs. 8.20–21, 24.

2015 *Sweetocristatus unicus* Dagis; Chen et al., fig. 8.23.

2016 *Hadrodontina aequabilis* Staesche; Koike, pp. 164–167, fig. 2, nr. 1–3.

2018 *Hadrodontina aequabilis* Staesche; Maekawa in Maekawa et al., p. 18, figs. 13.3?–13.4 (only).

2020 *Hadrodontina aequabilis* Staesche; Sun et al., figs. 1–7 (natural assemblage of the *Hadrodontina* apparatus).

P_2_ element:

*1964 *Hadrodontina aequabilis* n. sp.; Staesche, p. 275, fig. 44.

2004 *Ellisonia* sp. aff. *E. triassica* Müller; Koike et al., p. 247, fig. 8.6

2016 *Hadrodontina aequabilis* Staesche; Koike, p. 165, figs. 2.4 (P2 element).

2020 *Hadrodontina aequabilis* Staesche; Sun et al., figs. 1–7 (natural assemblage of the *Hadrodontina* apparatus).

S_2_ element.

2004 *Ellisonia* sp. aff. *E. triassica* Müller; Koike et al., p. 247, fig. 8.4

2016 *Hadrodontina aequabilis* Staesche; Koike, p. 165, figs. 2.9–11.

2020 *Hadrodontina aequabilis* Staesche; Sun et al., figs. 1–7 (natural assemblage of the *Hadrodontina* apparatus).

*Material.* P_1_, more than 40; P_2_, more than 40; S_2_ more than 10.

*Revised diagnosis.* Robust to slender angulate P_1_ element. The 6–8 (usually 7) denticles are rather short, radiating, conical, with subtriangular free ends and they are increasingly reclined towards the posterior. The concave lower side is occupied by a large and wide groove.

*Remarks*. The observed variation in the denticulation of the P_1_ and P_2_ elements suggests there may be scope for further specific differentiation: in some elements the height of the denticles changes smoothly along the unit (Fig. 28B), whereas in others the denticles are alternatively high and low (Fig. 28D). Dagis (1984) assigned some specimens (here considered as junior synonyms of *Ha. aequabilis*) to *Sweetocristatus unicus*. Yet, the genus *Sweetocristatus* Szaniawski (in Szaniawski & Malkowski, 1979) was established for Upper Artinskian to Lower Guadalupian (Permian) P_1_ elements that, although superficially similar to homologous elements of *Hadrodontina* and *Pachycladina,* bear a more developed, higher cusp, a higher carina and have a more elongated process. The cusp in *Ellisonia* P_1_ elements is more conspicuous than in those of *Hadrodontina* or *Pachycladina*. Chen et al. (2015) illustrated coeval specimens from Jiarong they assigned to either *Sweetocristatus unicus* or *Parachirognathus peculiaris* (see their figs. 8.20 and 8.23) but we fail to distinguish them from *Hadrodontina aequabilis*. With three natural assemblages of *Hadrodontina aequabilis* from the late Smithian Helongshan Formation, South China, Sun et al. (2020) observations fits with our findings; the angulate shape and no distinct cusp in the P_1_ element and the distally twisting shape of the P_2_ element.

*Occurrence*. Russia; Smithian Hedenstroemia and tardus zone, Siberia (Dagis 1984), Zhitkov Formation, Smithian age, Abrek Bay area, South Primorye (Shigeta, 2009). China; Beisi Formation, Taiping, Pingguo Western Guangxi Province (Shunxin et al., 1997), Luolou Formation, Smithian age, Jiarong and Bianyang, Nanpanjiang Basin, southern Guizhou Province (Chen et al., 2015; Yan et al., 2013), Late Smithian Helongshan Formation, Chaohu, Anhui Province (Sun et al., 2020). Japan: Taho Formation, Shirokawa-cho, Higashiuwa-gun, Ehime Prefecture (below Smithian–Spathian boundary) (Koike 2016; Koike et al., 2004). Europe: Campiller member, Werfen Formation, Skyth, South Tirol, Italy (Staesche, 1964), Slovenia (Kolar-Jurkovšek & Jurkovšek, 2019).

## Supplementary Information

**Additional file 1: Fig. S1. **NMBY: Northern marginal basin of Yangtze, 1: Qiakong, 2: Laren, 3: Shanggang, 4: Lilong, 5: Youping Cascade, JR: Jiarong, MTL: Motianling, MT: Mingtang, GD: Guandao, BY: Bianyang,QY: Qingyan, SDZ: Sidazhai. GHQ: Ganheqiao, YWG: Yiwagou, DXK: Daxiakou, GX: Ganxi, QS: Qinshan, LT: Longtan, PDS (N&W): North and West Pingdingshan, JS: Jianshi. Yellow circles: studied sections by ourself. Red circles: studied sections from the literature. White circles with pink outline: sections from the literature which were excluded in the Unitary Association analysis.

**Additional file 2.** Exel file with the Unitary Associaton matrix. The raw data, the 1 run, the 8 run without *Pg. peculiaris* and the final 9 run are provided.

**Additional file 3.** Solutions for the Unitary Assosications analysis. After every run, the corresponding actions to reduce the contradictions are explained in detail.

**Additional file 4.**  Carbon isotope data from Youping Cascade.

**Additional file 5.** Unitary Association zone durations (from Widmann et al. 2020).

## Data Availability

The datasets (carbon isotope samples and conodont specimens) analysed during the current study are available from the corresponding author on reasonable request and stored at the Paleontological Institute and Museum, University of Zurich (Karl-Schmid-Strasse 4, 8006 Zürich, Switzerland). The data that support the findings of this study (for the UAM) are available through the public available journals referred herein.

## Abbreviations

BAN: Banhaipo (Laren)
CAI: Conodont Alteration Index
CIE: Carbon isotope excursion
FO: First occurrence
GDB: Griesbachian–Dienerian boundary
IOB: Induan–Olenekian boundary
LAR: Laren
LIL: Lilong
PTBME: Permian–Triassic boundary mass extinction
QIA: Qiakong
SHA: Shanggang
SSB: Smithian–Spathian boundary
UA: Unitary Association
UAM: Unitary Association method
UAZ: Unitary Association Zones
YC: Youping cascade
