# Supplementary material for: A Unitary Association-based conodont biozonation of the Smithian–Spathian boundary (Early Triassic) and associated biotic crisis from South China
Source: Swiss J Palaeontol. 2022 Nov 22;141(1):19. doi: 10.1186/s13358-022-00259-x (PMC9681704; doi:10.1186/s13358-022-00259-x)
Supplement: Supplementary file 1 — Additional file 1: Fig. S1. NMBY: Northern marginal basin of Yangtze, 1: Qiakong, 2: Laren, 3: Shanggang, 4: Lilong, 5: Youping Cascade, JR: Jiarong, MTL: Motianling, MT: Mingtang, GD: Guandao, BY: Bianyang,QY: Qingyan, SDZ: Sidazhai. GHQ: Ganheqiao, YWG: Yiwagou, DXK: Daxiakou, GX: Ganxi, QS: Qinshan, LT: Longtan, PDS (N&W): North and West Pingdingshan, JS: Jianshi. Yellow circles: studied sections by ourself. Red circles: studied sections from the literature. White circles with pink outline: sections from the literature which were excluded in the Unitary Association analysis. [file 13358_2022_259_MOESM1_ESM.pdf]

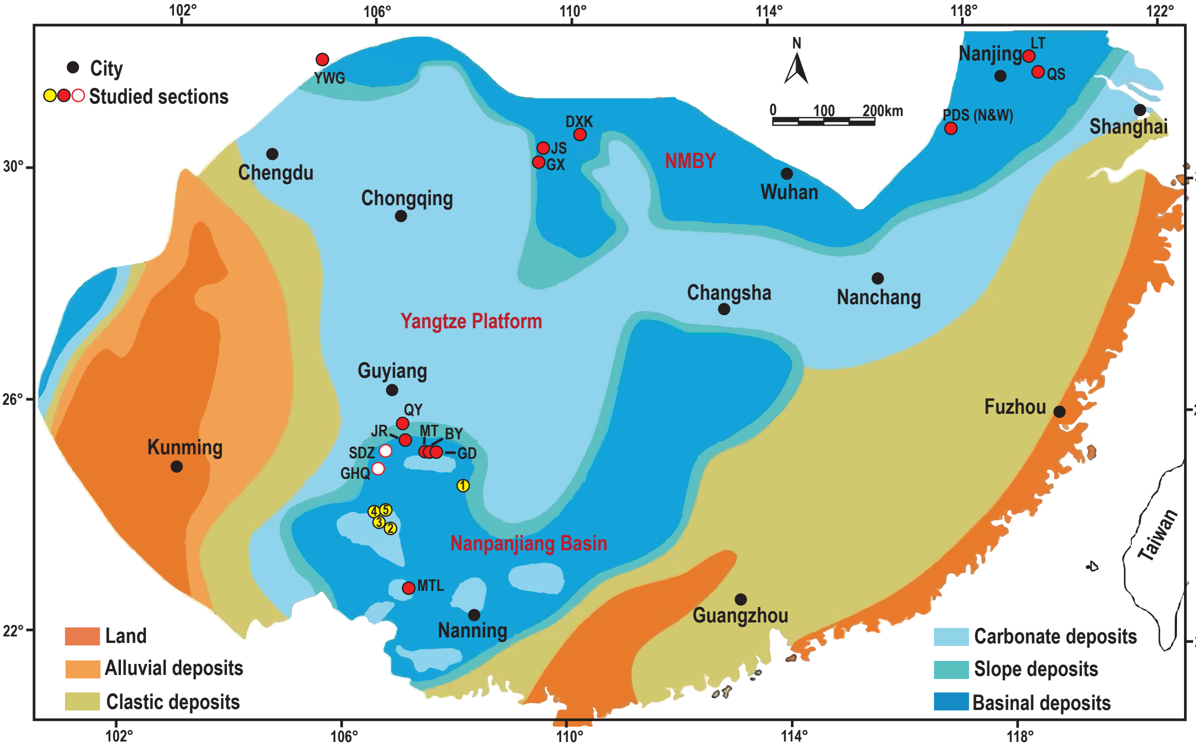

Additional file 1: Fig. S1; NMBY: Northern marginal basin of Yangtze, 1: Qiakong, 2: Laren, 3: Shanggang, 4: Lilong, 5: Youping Cascade, JR: Jiarong, MTL: Motianling, MT: Mingtang, GD: Guandao, BY: Bianyang, QY: Qingyan, SDZ: Sidazhai. GHQ: Ganheqiao, YWG: Yiwagou, DXK: Daxiakou, GX: Ganxi, QS: Qinshan, LT: Longtan, PDS(N&W): North and West Pingdingshan, JS: Jianshi. Circles, yellow: own studied sections. red: sections included from the literature. white: sections from the literature which were removed from the Unitary association analysis.
