# Supplementary material for: A Unitary Association-based conodont biozonation of the Smithian–Spathian boundary (Early Triassic) and associated biotic crisis from South China
Source: Swiss J Palaeontol. 2022 Nov 22;141(1):19. doi: 10.1186/s13358-022-00259-x (PMC9681704; doi:10.1186/s13358-022-00259-x)
Supplement: Supplementary file 3 — Additional file 3. Solutions for the Unitary Assosications analysis. After every run, the corresponding actions to reduce the contradictions are explained in detail. [file 13358_2022_259_MOESM3_ESM.docx]

**UA files solutions**

**Initial taxonomical cleaning**

Taxonomical identification was standardized to produce reliable raw data. See taxon matrix in supplementary material and systematic taxonomy part in main article. The initial taxonomical cleaning includes among others the following:

🡪Synonymize *Nv. spitiensis* and *Ns. spitiensis* to *Ns. spitiensis* (deleting *Nv. spitiensis* in Mot5 and Wping6, adding *Ns. spitiensis* in Mot5 and Wping6).

🡪Include *Sc. milleri* parva within the intraspecific variation of *Sc. milleri* in Longtan and Qingshan (Long37 and Qisha38. This subspecies will be try to be separated again at the end of the “contradiction solving” if no additional contradictions occurs.

🡪Keeping *Gl. laii* morphotype A (initially determined as *Gl*. n. sp. A) in Long77. Although the illustrated specimen from Long75 were re-assigned to *Gl. laii* morphotype B, it can not be excluded that the specimes found in Long77 (not illustrated) belong indeed to morphotype A (see systematic paleontology part in main text).

**Run 1 (all sections)**

- Residual horizons: 95
- Max cliques 63
- Unitary associations: 28
- Contradictions: 453
- Cliques in cycles: 31
- Residual virtual edges: 8
- Z_4_ cycles: 313

🡪solution: deleting the sections Sidazhai and Ganheqiao from study because they are creating too many contradictions. Probably mixed faunas due to sedimentology. See main text.

**Run 1 (all remaining sections)**

- Residual horizons: 78
- Max cliques 49
- Unitary associations: 30
- Contradictions: 164
- Cliques in cycles: 0
- Residual virtual edges: 4
- Z_4_ cycles: 100

**Deleting rare, badly defined and/or long ranging species**

🡪delete *Nv*. n. sp. A because it creates many Z_4_-cycles (9 in total) and contradicitons. Furthermore this is a new species in open nomenclature (all contradictions of own studied sections including the Jiarong section are solved).

🡪Deleting *Conservatella* spp. (= *Cn. conservativa*) because this species is involved in many Z_4_-cycles (5) and is furthermore not yet a very well-established taxon on the species level (sometimes it stays in open nomenclature). Although this taxon is common in other regions (e.g. USA), it is a genus rarely found in South China.

- 140 contradicitons and 81 Z_4_-cycles left after deleting *Conservatella* spp. and *Nv*. n. sp. A from the data set.

🡪Deleting *Ns. pakistanensis* and *Ns. novaehollandiae* from list because they create Z_4_-cycles (5 for *Ns. novaehollandiae* and 12 for *Ns. pakistanensis* ) and 15 contradictions. These species are common and well documented from other regions (e.g. Western USA and Primorye) but rare in South China and therefore creating many contradicitions. Also synomymize these two species r into *Ns*. ex gr. *pakistanensis* as some authors (e.g. Matsuda 1983) suggest does not resolve the remaining contradictions as the two species occur mostly in association to each other.

- 125 contradicitons and 64 Z_4_-cycles left after deleting *Ns. pakistanensis* and *Ns. novaehollandiae* from data set.

🡪Delete *Nv. eotriangularis*: this is a very rare species (found only in 2 sections) but apparently long ranging, creating 3 contradictions. Delete from Nping and from Qisha. In the authors view, this species is furthermore insufficient reported and needs further investigation.

**Run 2 (all remaining sections)**

- Residual horizons: 70
- Max cliques 42
- Unitary associations: 24
- Contradictions: 122
- Cliques in cycles: 0
- Residual virtual edges: 0
- Z_4_ cycles: 64

**Deleting certain ranges of species based on lack of illustrations or absence of co-occurences in other all sections**

🡪 Delete *Ns. bevelledi* n. sp. (=*Ns*. n. sp. V) from Jia2 because only a single occurrence is shown without any illustrations and in other sections (with illustrations) this species is always found in younger faunas and never together with *Ns.* ex gr. *cristagalli* and *Eurygnathodus costatus*. Furthermore, this new species was not properly determined and described yet in Lyu et al. (2019). Only a small illustration from Orchard (2007) could be used to determine the specimen as *Ns*. n. sp. V which could be in a too broad sense.

🡪120 contradicitons and 62 Z_4_-cycles left.

🡪 Problem: in many Z_4_-cycles and contradictions are *Ic. crassatus* and *Ic*. aff. *crassatus* involved. Solution: synonymize them together to «*Ic*. ex. gr. *crassatus*» . Most specimens of *Ic*. aff. *crassatus* were determined by the here 5 new studied sections. This may have been a result of too much “splitting”. However, many illustrated specimens of other studies which were included in the UA method are morphologically more similar to *Ic*. aff. *crassatus* (e.g. Li et al. 2011, Liu et al. 2019) than to *Ic. crassatus* sensu stricto (see systematic taxonomy in main text).The authors recognize the morphological affinity of the described *Ic*. aff. *crassatus* (see systematic taxonomy) and the solutions to many contradictions by using the UA method, however when compared to the holotype material from Oman (Orchard 1995), the morphological differences are clearly recognizable by a lower carina and a less developed asymmetrical basal cavity.

🡪67 contradicitons and 43 Z_4_-cycles left.

**Run 3 (all remaining sections)**

- Residual horizons: 69
- Max cliques 38
- Unitary associations: 25
- Contradictions: 67
- Cliques in cycles: 0
- Residual virtual edges: 1
- Z_4_ cycles: 19

🡪Re-determine and rename *Ic*. ex gr. *crassatus* from Nping4 (determined as *Ic*. aff. *crassatus*). The illustrated specimen from Zhao 2005 from bed 61 (Pl. 12 fig. 11) is morphologically more similar to *Nv. expansus* than to *Ic*. aff. *crassatus* (Leu & Goudemand, this study). Therefore this specimen is considered as *Nv. expansus* (taxonomical standardization).

🡪61 contradicitons and 43 Z_4_-cycles left.

🡪 delete *Nv. pingdingshanensis* from Qin 10, Qin9, Qin8, Qin7, and Qin6 and Qin 5 because this species was nowhere else found together with *Sp. spathi*, *Nv. brochus* and/or *Tr. homeri* and the illustrated specimens are not *Nv. pindingshanensis* sensu stricto, but rather juvenile forms of *Triassospathodus* sp. In Qin10, Qin9, Qin8 and Qin6, this taxon is only virtually present. Furthermore, the recovering of this species from limestone breccia must be evaluated with caution as it might be a result of reworking. With deleting Nv. pingdingshanensis from the above mentioned LMHs, 6 contradictions and 11 Z_4_-cycles are solved.

🡪55 contradicitons and 32 Z_4_-cycles left.

🡪Delete *Nv.* ex gr. *waageni* from Wping 9 and Wping10 because it was never found elsewhere toghether with *Nv. brevissimus* and illustrations in Liang et al., 2011 can not be clearly determined as *Nv. waageni* sensu lato. Fig. 3 nr. 7 (in Liang et al. 2011), the illustrated specimen shows a reclined denticles in the posterior three-quarter part. This resembles more the *Nv. pingdingshanensis* group. The later species is already in the data set in Wping9 and Wping10 and does not have to be included anymore. The illustrated specimen in fig. 3 nr. 9 is very badly preserved (re-crystalized) and broken in the posterior and anterior part. A clear determination is not possible for this specimen. Furthermore *Nv.* ex gr. *waageni* could not be found in beds above Bed 52 in Zhao (2005) and above bed 53 in Zhao et al. (2008).

🡪60 contradicitons and 36 Z_4_-cycles left.

🡪Delete *Nv.* ex gr. *waageni* from Min3 because it was never found together with *Spathicuspus spathi, Novispathodus triangularis, Triassospathodus homeri*, *Spathicuspus*? n. sp. C and other typical Spathian fauna conodonts elsewhere. Furthermore is the youngest occurrence of this species found in a brecciaed dolomite and no illustration of Novispathodus waageni is provided for bed 17 in Mingtang (Liang et al. 2016). This solution solves 10 contradicitons and 5 Z_4_-cycles.

🡪45 contradicitons and 27 Z_4_-cycles left.

🡪Extend *Nv.* ex gr. *abruptus* in Shanggang to SHA 11 (9, 10, 11) because it has a co-occurence with *Tr. homeri* in many other sections (solving 13 contradictions and 5 Z_4_-cycles).

🡪32 contradicitons and 22 Z_4_-cycles left.

**Run 4 (all remaining sections)**

- Residual horizons: 68
- Max cliques 33
- Unitary associations: 24
- Contradictions: 32
- Cliques in cycles: 0
- Residual virtual edges: 1
- Z_4_ cycles: 22

🡪 Extend *Nv. clinatus* in Laren to LAR11 (because it is a rare species) (solving 6 contradictions, it is therefore the most parsimonious way to solve maximum amount of contradictions with minimal extension in a species range). *Nv. clinatus* is involved in easily to determine contradicitons where the association like in Lil6 could be found 13 times more than the association as in Lar 10 where *Nv. clinatus* was found below Ng. n. sp. B*, Tr.* aff. *symmetricus*, *Ic. zaksi* and *Ic. crassatus*.

🡪26 contradicitons and 22 Z_4_-cycles left.

🡪Extend occurrence of *Ns. posterolongatus* In Jianshi to Jia2 because it occurs also younger than *Eurygnathodus* beds in other sections (e.g. Motianling). This solves all residual virtual edges and 3 contradicitons.

🡪23 contradicitons and 22 Z_4_-cycles left.

🡪Delete *Nv. waageni* from Qisha60 and Qisha70 beause there are no illustrations and it solves 11 Z_4_ cycles. The only illustration from Qisha70 (Liu et al., Pl. 2 fig. 7) cannot be assigned to *Nv. waageni* sensu lato. The specimen has a typical sinusoid-shaped lower margin as in *Nv. waageni*. However, this is not a determined characteristic for *Nv. waageni*. And the overall shape resembles more Spathian species like *Novispathodus radialis* or the more short and robust form *Novispathodus cyclodontus* with erect denticles (Zhao et al. 2008). This specimen is here determined as *Nv.* sp indet. because the illustrated specimen is broken at the posterior end and the anterior upper end. The associated species in Qisha60 and Qisha70 are typical Spathian species such as *Ic. crassatus, Nv. brevissimus, Nv. eotriangularis, Nv. pingdingshanensis* and *Tr. symmetricus*.

🡪23 contradicitons and 11 Z_4_-cycles left.

**Run 5 (all remaining sections)**

- Residual horizons: 66
- Max cliques 32
- Unitary associations: 24
- Contradictions: 23
- Cliques in cycles: 0
- Residual virtual edges: 0
- Z_4_ cycles: 11

🡪 delete *Nv*. ex gr. *abruptus* from Long 57 because It was initially determined as *Nv*. aff. *abruptus*. The only illustrated specimen of *Nv.* aff. *abruptus* is from Long57 (Pl. 3 fig. 11, Liu et al. 2019). The illustrated specimen has a more elongated basal cavity compared to the holotype. Furthermore the upper arcuated profile with the increasing height of denticles towards the posterior (with the exception of the 2 posteriormost denticles) looks superficially as most of the denticles are broken. Especially the anterior part is badly persevered. In the posterior part. The basal cavity is abruptly trunctuated which shows more similarities with specimens of the genus *Triassospathodus*.

🡪23 contradicitons and 11 Z_4_-cycles left.

🡪 delete *Tr*. *homeri* from Long 70 because no clear determination can be made and this occurrence is involved in many contradictions and Z_4_-cycles. This species was initially determined as *Tr*. aff. *homer*i. The illustrated specimen (Pl. 4, fig. 1-2, Liu et al. 2019) are only shown in aboral and oral view. The determination is therefore difficult. The specimen in Pl. 4, fig. 2 (Liu et al. 2019) looks symmetrical in both (aboral and oral) view which indicates more affinity to *Tr. symmetricus.* Also on the illustrated figure 1, in the aboral view, The basal cavity is not taperint in both anterior and posterior directions. The characteristic diagnosis with an inturned posterior process can not be resolved. The co-occurrence of Tr. homeri in Long70 with *Nv*. aff. *clinatus*, *Nv*. *pingdingshanensis*, *Tr. symmetricus*, *Nv. abruptus* and *Tr. triangularis* can be questioned as in other sections Tr. homeri never co-occurred with *Nv. pingdingshaneinsis*. With the deletion of *Tr. homeri* from Long70, 5 contradictions and 6 Z_4_-cycles can be solved.

🡪18 contradicitons and 5 Z_4_-cycles left.

🡪Delete *Nv. waageni* from Nping5 because it was never found elsewhere together with *Sp. spathi* (neither on the same section by Zhao 2005). This solves 8 contradictions. The species was found in the Columbites-Tirolites ammonoid zone in the (Spathian) Helongshan Formation. Furthermore was this taxon in Nping5 initially determined as *Neospathodus* aff. *waageni eowaageni* and synonymized with *Nv. waageni* by the process of taxonomic standardization. Although there is no illustration of this specimen, a later taxonomical revision by Zhao et al. (2008) indicates that this specimen could might have been re-named and determined as the new species *Nv. posteowaageni*.

🡪10 contradicitons and 5 Z_4_-cycles left.

🡪 extend *Sc. milleri* and *Sc. mosheri* upwards to Yiw4 and *Sc. milleri* upwards to Long57 and Qisha 42 because of their co-occurences with *Nv. pingdinshanensis* and *Bo. buurensis* in many other sections (e.g. Orchard 2008).

🡪11 contradicitons and 5 Z_4_-cycles left.

🡪Delete *Nv.* ex gr. *pingdingshanensis* from Nping5 and Nping6 because also it was never found together with *Sp. spathi* in other sections and no illustrations are provided. By deleting *Nv.* ex gr. *pingdingshanensis* from Nping5 and Nping6, it will solve the following contradiction: In the dataset are 2 cliques where *Ic. zaksi* is below *Sp. spathi* and 5 cliques where *Ic. zaksi* is below *Tr. homeri*. There are also 7 cliques with *Nv.* ex gr. *pingdingshanensis* below Ng. n. sp. B. However, in Lil6, there is a co-occurrence of Ic. zaksi with Ng. n. sp. B and in Nping6 there is a co-occurrence of *Nv*. ex gr. *pingdingshanensis* with *Sp. spathi* and *Tr. homeri.* Although the amount of cliques are the same in both directions, we think the probability of the co-occurrence of *Nv*. ex gr. *pingdingshanensis* with *Sp. spathi* and *Tr. homeri* is less likely.

**Run 5 (all remaining sections)**

- Residual horizons: 63
- Max cliques 31
- Unitary associations: 24
- Contradictions: 10
- Cliques in cycles: 0
- Residual virtual edges: 0
- Z_4_ cycles: 3

🡪Extend *Sp*.? n. sp. C downwards in SHA11 and SHA10 because of co-occurrence with *Ic. collinsoni* in Laren. (solves 1 contradiction). See figure above.

**Run 6 (all remaining sections)**

- Residual horizons: 63
- Max cliques 31
- Unitary associations: 24
- Contradictions: 9
- Cliques in cycles: 0
- Residual virtual edges: 0
- Z_4_ cycles: 3

In most remaining contradictions is the section West Pingdingshan involved. The West Pindingshan section was studied several times (Liang et al., (2011), Zhao et al. (2008) and Zhao (2005)) with different conodont range charts in all these studies. The conodont range chart used for this study wa taken from Liang et al. (2011). The following adjustments were made to find the most parcimonious way to solve most contradictions and being in agreement with all 3 published studies.

🡪 Delete *Ic.* ex gr. *crassatus* from Wping10 because it was not found in Zhao 2005 and Zhao et al. 2008 in older beds before the co-occurence with *Tr. homeri.* Furthermore are in Liang et al. (2011) no illustrations provided for *Ic.* ex gr. *crassatus* whereas in the study from Zhao 2005, the determination of *Ic.* ex gr. *crassatus* can be confirmed including illustrations.

- Solving 1 contradiction

🡪*Nv. brevissimus* was upwardly extended to Wping10 as it was found and illustrated in Zhao 2005. Furthermore was *Nv. brevissimus* deleted from Wping 9 as neither Zhao et al. 2008 nor Zhao 2005 could recover this specimen from bed 54. Furthermore is the recovery by Liang et al. 2011 not conclusive as no illustrations are provided.

*Nv. expansus* was deleted from Wping10 as the uppermost occurrence was never illustrated (The holotype is from Wping9). Furthermore was Nv. expansus downwardly extended to Wping8 as found as *Ns.* n. sp. R by Zhao 2005. This is the most parcimonious way, resolves most contradicitons and is in agreement with all different conodont range occurrences published form from the West Pingdingshan section (in Liang et al., (2011), Zhao et al. (2008) and Zhao (2005)).

- 4 contradictions and 1 Z4 cycle left.

**Run 7**

- Residual horizons: 61
- Max cliques 30
- Unitary associations: 23
- Contradictions: 4
- Cliques in cycles: 0
- Residual virtual edges: 0
- Z_4_ cycles: 1

In most remaining contradictions is *Pg. peculiaris* involved.

🡪Deleting *Pg. peculiaris* from data set to solve all contradicitons

**Run 8**

- Residual horizons: 60
- Max cliques 29
- Unitary associations: 23
- Contradictions: 0
- Cliques in cycles: 0
- Residual virtual edges: 0
- Z4 cycles: 1

🡪All contradictions are solved except for 1 Z_4_-cycles which is difficult to resolve.

**Steps used to re-introduce *Pg. peculiaris***

The following steps are used to re-introduce *Pg. peculiaris* in the UAs.

🡪Upwardly extend occurrence of *Ns. chaohuensis* to Wping3 and Wping4 because of co-occurence with *E. costatus* in Jiache.

🡪Upwardly extend *Ns*. ex. gr. *cristagalli* to Wping5 because of co-occurrence with *E.hamadai* in 3 other sections and co-occurrence in West Pingdingshan with *Ns. spitiensis* in Zhao 2005.

🡪 extend *Pg. peculiaris* downwards in Bia3 and Bia2 to co-occurrence with *Eu. costatus* because it is found in Motianling together with *Eu. costatus.*

🡪 downwardly extend *Di. discreta* in YC2 and YC1 because of co-occurrence with *Di*. n. sp. B in Shanggang (no contradictions within my 5 sections).

🡪Extend upwardly *Nv*. ex gr. *waageni* in Laren to Lar 7 because of co-occurrence of *Nv*. ex gr. *waageni* wit *Nv*. ex gr. *abruptus* and *Nv*. ex gr. *pingdingshanensis* in Jiarong and Ganxi.

🡪Introduce *Nv.* ex gr. *waageni* from Qia2 to Qia4. This taxon was not included from Qia2-Qia4 in the first run because the found elements were broken (*Nv*. cf. *waageni*) and therefore not considered as taxonomical stable enough to include here.

🡪Extend *Pg. peculiaris* upwards to Mot5 (most parcimonious way).

**Run 9**

- Residual horizons: 59
- Max cliques 32
- Unitary associations: 24
- Contradictions: 0
- Cliques in cycles: 0
- Residual virtual edges: 0
- Z4 cycles: 5

🡪All contradictions are solved except for 5 Z_4_-cycles which are difficult to resolve.

Most included species in the Z_4_-cycles are *Ns. spitiensis* and *Pc. peculiaris*
