# Supplementary material for: A Unitary Association-based conodont biozonation of the Smithian–Spathian boundary (Early Triassic) and associated biotic crisis from South China
Source: Swiss J Palaeontol. 2022 Nov 22;141(1):19. doi: 10.1186/s13358-022-00259-x (PMC9681704; doi:10.1186/s13358-022-00259-x)
Supplement: Supplementary file 4 — Additional file 4. Carbon isotope data from Youping Cascade. [file 13358_2022_259_MOESM4_ESM.pdf]

Sample	Weight (µg, mg)	Ampl 44	Area All	Avg. d13C raw	std.dev.	Avg. d18O raw
YC40	245	3762	26.063	1.14	0.04	33.09
YC44	257	4425	30.454	3.37	0.04	32.79
YC13d	267	2537	17.413	-0.92	0.07	32.10
YC23	265	3376	23.326	-1.02	0.06	32.38
YC10	220	938	6.273	-0.58	0.10	31.76
YC39	239	3070	21.12	2.23	0.02	32.34
YC24	268	4566	31.055	-1.18	0.04	33.03
YC25	262	3940	27.39	-1.07	0.04	32.88
YC16	238	3073	21.202	-1.11	0.05	32.87
YC18	257	3422	23.557	-1.33	0.04	32.11
YC47	248	3076	21.269	2.26	0.03	31.96
YC41	258	3743	25.888	1.12	0.04	32.55
YC35b	269	3292	23.064	-0.08	0.02	32.29
YC35a	254	2510	17.175	-0.15	0.07	32.93
YC12	229	3603	24.855	-0.96	0.03	32.82
YC46	240	3264	22.445	2.80	0.05	33.07
YC43	239	3807	26.221	2.68	0.07	32.77
YC42	283	5049	35.244	3.23	0.05	32.71
YC28	284	3087	21.547	-0.91	0.05	32.33
YC27	215	2261	15.745	-1.33	0.03	32.08

Sample	std.dev.	$\delta^{13}C$ VPDB	$\delta^{18}O$ VPDB Calcite	$\delta^{18}O$ VSMOW Calcite	Yield % (as CaCO3, CO3)
YC40	0.09	1.10	-5.95	24.78	70
YC44	0.15	3.33	-6.25	24.47	78
YC13d	0.09	-0.96	-6.91	23.79	43
YC23	0.08	-1.06	-6.64	24.07	58
YC10	0.12	-0.62	-7.23	23.46	19
YC39	0.06	2.19	-6.67	24.03	58
YC24	0.19	-1.23	-6.01	24.71	77
YC25	0.11	-1.11	-6.16	24.56	69
YC16	0.18	-1.15	-6.17	24.55	59
YC18	0.07	-1.37	-6.90	23.80	61
YC47	0.06	2.22	-7.04	23.65	57
YC41	0.09	1.07	-6.47	24.24	66
YC35b	0.05	-0.12	-6.72	23.98	57
YC35a	0.19	-0.19	-6.11	24.61	45
YC12	0.14	-1.01	-6.21	24.51	72
YC46	0.18	2.75	-5.98	24.75	62
YC43	0.16	2.64	-6.26	24.45	72
YC42	0.08	3.19	-6.32	24.40	82
YC28	0.06	-0.95	-6.69	24.02	50
YC27	0.10	-1.38	-6.93	23.77	48
