# Supplementary material for: A Unitary Association-based conodont biozonation of the Smithian–Spathian boundary (Early Triassic) and associated biotic crisis from South China
Source: Swiss J Palaeontol. 2022 Nov 22;141(1):19. doi: 10.1186/s13358-022-00259-x (PMC9681704; doi:10.1186/s13358-022-00259-x)
Supplement: Supplementary file 5 — Additional file 5. Unitary Association zone durations (from Widmann et al. 2020). [file 13358_2022_259_MOESM5_ESM.pdf]

| | | | | | | | | | | | |
|---|---|---|---|---|---|---|---|---|---|---|---|
| 1m = | 2.322 | cm | | | m | | | | | | mo |
| Qiakong | min | max | | min | max | | | min | max | median | mean |
| UAZ9 | 2.7 | 30.7 | | 1.16 | 13.22 | | 116 | 248757 | 248888 | 248823 | 248823 |
| UAZ8 | 31.2 | 31.8 | | 13.44 | 13.70 | | 1344 | 249057 | 249178 | 249119 | 249118 |
| UAZ7 | 33.2 | 35.92 | | 14.30 | 15.47 | | 1430 | 249136 | 249298 | 249211 | 249213 |
| UAZ6 | 37.2 | 40.53 | | 16.02 | 17.45 | | 1602 | 249268 | 249431 | 249347 | 249348 |
| UAZ5 | 43.7 | 50.949 | | 18.82 | 21.94 | | 1822 | 249442 | 249713 | 249566 | 249568 |
| UAZ4 | 52.8 | 54.1 | | 22.74 | 23.30 | | 2274 | 249759 | 250029 | 249888 | 249889 |
| UAZ3 | | | | | | | | | | | |
| UAZ2 | | | | | | | | | | | |
| UAZ1 | | | | | | | | | | | |
| | | | | | | | | | | | |
| Laren | min | max | | min | max | | | | | | |
| UAZ9 | 0 | 17 | | 0.00 | 7.32 | | | 248778 | 248922 | 248851 | 248850 |
| UAZ8 | | | | 0.00 | 0.00 | | | | | | |
| UAZ7 | 18.2 | 20.5 | | 7.84 | 8.83 | | 784 | 249216 | 249435 | 249324 | 249324 |
| UAZ6 | | | | 0.00 | 0.00 | | | | | | |
| UAZ5 | 45.9 | 48.5 | | 19.77 | 20.89 | | 896 | 249330 | 249548 | 249437 | 249438 |
| UAZ4 | 29 | 30.5 | | 12.49 | 13.14 | | 1249 | 249682 | 249915 | 249799 | 249799 |
| UAZ3 | | | | | | | | | | | |
| UAZ2 | | | | | | | | | | | |
| UAZ1 | | | | | | | | | | | |
| | | | | | | | | | | | |
| Shanggang | min | max | | min | max | | | | | | |
| UAZ9 | 1 | 15.2 | | 0.43 | 6.55 | | 43 | 248797 | 248950 | 248874 | 248874 |
| UAZ8 | | | | 0.00 | 0.00 | | | | | | |
| UAZ7 | | | | 0.00 | 0.00 | | | | | | |
| UAZ6 | 19.8 | 20.6 | | 8.53 | 8.87 | | 853 | 249276 | 249439 | 249356 | 249357 |
| UAZ5 | 22.8 | 25.33 | | 9.82 | 10.91 | | 982 | 249371 | 249554 | 249462 | 249463 |
| UAZ4 | | | | 0.00 | 0.00 | | | | | | |
| UAZ3 | 27.8 | 39.6 | | 11.97 | 17.05 | | 1197 | 249546 | 249728 | 249639 | 249638 |
| UAZ2 | | | | | | | | | | | |
| UAZ1 | | | | | | | | | | | |

| | | | | | | | | | | | |
|---|---|---|---|---|---|---|---|---|---|---|---|
| 1m = | odel parameter | | | | | | | dage kyrs | | | |
| Qiakong | | | min | max | median | mean | Qiakong | | 2s | 2s | error |
| UAZ9 | | 1322 | 249052 | 249173 | 249114 | 249113 | UAZ9 | 290 | 66 | 61 | 89 |
| UAZ8 | | 1370 | 249063 | 249184 | 249125 | 249125 | UAZ8 | 7 | 61 | 61 | 86 |
| UAZ7 | | 1547 | 249228 | 249382 | 249302 | 249304 | UAZ7 | 91 | 81 | 77 | 112 |
| UAZ6 | | 1745 | 249381 | 249623 | 249492 | 249495 | UAZ6 | 147 | 82 | 121 | 146 |
| UAZ5 | | 2175 | 249686 | 249941 | 249808 | 249809 | UAZ5 | 241 | 136 | 128 | 186 |
| UAZ4 | | 2330 | 249799 | 250075 | 249932 | 249934 | UAZ4 | 45 | 135 | 138 | 193 |
| UAZ3 | | | | | | | UAZ3 | | | | |
| UAZ2 | | | | | | | UAZ2 | | | | |
| UAZ1 | | | | | | | UAZ1 | | | | |
| | | | | | | | | | | | |
| Laren | | | | | | | Laren | | | | |
| UAZ9 | | 732 | 249063 | 249233 | 249148 | 249148 | UAZ9 | 298 | 72 | 85 | 111 |
| UAZ8 | | | | | | | UAZ8 | 0 | 0 | 0 | 0 |
| UAZ7 | | 883 | 249318 | 249534 | 249424 | 249425 | UAZ7 | 101 | 110 | 108 | 154 |
| UAZ6 | | | | | | | UAZ6 | 0 | 0 | 0 | 0 |
| UAZ5 | | 1111 | 249544 | 249774 | 249657 | 249658 | UAZ5 | 220 | 109 | 115 | 158 |
| UAZ4 | | 1314 | 249747 | 249980 | 249866 | 249865 | UAZ4 | 66 | 117 | 117 | 165 |
| UAZ3 | | | | | | | UAZ3 | | | | |
| UAZ2 | | | | | | | UAZ2 | | | | |
| UAZ1 | | | | | | | UAZ1 | | | | |
| | | | | | | | | | | | |
| Shanggang | | | | | | | Shanggang | | | | |
| UAZ9 | | 655 | 249044 | 249199 | 249122 | 249121 | UAZ9 | 247 | 77 | 78 | 109 |
| UAZ8 | | | | | | | UAZ8 | | | | |
| UAZ7 | | | | | | | UAZ7 | | | | |
| UAZ6 | | 887 | 249301 | 249470 | 249384 | 249385 | UAZ6 | 28 | 82 | 85 | 117 |
| UAZ5 | | 1091 | 249459 | 249645 | 249551 | 249552 | UAZ5 | 89 | 92 | 93 | 130 |
| UAZ4 | | | | | | | UAZ4 | | | | |
| UAZ3 | | 1705 | 250011 | 250161 | 250086 | 250086 | UAZ3 | 448 | 91 | 75 | 118 |
| UAZ2 | | | | | | | UAZ2 | | | | |
| UAZ1 | | | | | | | UAZ1 | | | | |

| | | | | | | | | | | |
|---|---|---|---|---|---|---|---|---|---|---|
| | | | | | | | | | | |
| Qiakong | | | | | | | | | | |
| top UAZ 6 | 38.443 | | | 16.56 | | 1656 | 249309 | 249480 | 249390 | 249391 |
| base of UAZ5 | 50.949 | | | 21.94 | | 2194 | 249700 | 249957 | 249823 | 249824 |
| | | | | | | | | | | |
| Laren | | | | | | | | | | |
| separation interval between the Anasi | 20.902 | | | 9.00 | | 900 | 249334 | 249553 | 249441 | 249442 |
| base of the onset of the positive CIE | 24.772 | | | 10.67 | | 1067 | 249498 | 249729 | 249612 | 249613 |
| | | | | | | | | | | |
| Shanggang | | | | | | | | | | |
| separation interval between the Ar | 19.776 | | | 8.52 | | 852 | 249286 | 249501 | 249393 | 249393 |
| base of UAZ5 | 25.137 | | | 10.83 | | 1083 | 249514 | 249745 | 249629 | 249629 |

| | | | | | | | | | | | |
|---|---|---|---|---|---|---|---|---|---|---|---|
| | | | | | | | | | | | |
| Qiakong | | | | | | | | | | | |
| top UAZ 6 | | | | | | | | 433 | 86 | 129 | 154 |
| base of UAZ5 | | | | | | | | | | | |
| | | | | | | | | | | | |
| Laren | | | | | | | | | | | |
| separation interval between the Anasi | | | | | | | | 171 | 110 | 116 | 159 |
| base of the onset of the positive CIE | | | | | | | | | | | |
| | | | | | | | | | | | |
| Shanggang | | | | | | | | | | | |
| separation interval between the Ar | | | | | | | | 236 | 108 | 116 | 158 |
| base of UAZ5 | | | | | | | | | | | |
